# A ferroptosis‐related LncRNAs signature for predicting prognoses and screening potential therapeutic drugs in patients with lung adenocarcinoma: A retrospective study

**DOI:** 10.1002/cnr2.1925

**Published:** 2023-12-03

**Authors:** Jiaxin Dong, Tao Tao, Jiaao Yu, Huisi Shan, Ziyu Liu, Guangzhao Zheng, Zhihong Li, Wanyi Situ, Xiao Zhu, Zesong Li

**Affiliations:** ^1^ Computational Systems Biology Lab (CSBL), The Marine Biomedical Research Institute Guangdong Medical University Zhanjiang China; ^2^ Medical Research Center, Department of Gastroenterology Zibo Central Hospital Zibo China; ^3^ Guangdong Provincial Key Laboratory of Systems Biology and Synthetic Biology for Urogenital Tumors, Shenzhen Key Laboratory of Genitourinary Tumor, Department of Urology The First Affiliated Hospital of Shenzhen University, Shenzhen Second People's Hospital (Shenzhen Institute of Translational Medicine) Shenzhen China

**Keywords:** ferroptosis, immunotherapy, lung adenocarcinoma, pRRophetic, tumor microenvironment

## Abstract

**Background:**

Lung adenocarcinoma (LUAD) has a high mortality rate. Ferroptosis is linked to tumor initiation and progression.

**Aims:**

This study aims to develop prognostic models of ferroptosis‐related lncRNAs, evaluate the correlation between differentially expressed genes and tumor microenvironment, and identify prospective drugs for managing LUAD.

**Methods and Results:**

In this study, transcriptomic and clinical data were downloaded from the TCGA database, and ferroptosis‐related genes were obtained from the FerrDb database. Through correlation analysis, Cox analysis, and the LASSO algorithm for constructing a prognostic model, we found that ferroptosis‐related lncRNA‐based gene signatures (FLncSig) had a strong prognostic predicting ability in the LUAD patients. Gene Ontology (GO) and Kyoto Encyclopedia of Genes and Genomes (KEGG) enrichments reconfirmed that ferroptosis is related to receptor‐ligand activity, enzyme inhibitor activity, and the IL‐17 signaling pathway. Next, tumor mutation burden (TMB), tumor immune dysfunction and exclusion (TIDE) algorithms, and pRRophetic were used to predict immunotherapy response and chemotherapy sensitivity. The IMvigor210 cohort was also used to validate the prognostic model. In the tumor microenvironment, Type_II_IFN_Response and HLA were found to be a group of low‐risk pathways, while MHC_class_I was a group of high‐risk pathways. Patients in the high‐risk subgroup had lower TIDE scores. Exclusion, MDSC, CAF, and TAMM2 were significantly and positively correlated with risk scores. In addition, we found 15 potential therapeutic drugs for LUAD. Finally, differential analysis of stemness index based on mRNA expression (mRNAsi) indicated that mRNAsi was correlated with gender, primary tumor (T), distant metastasis (M), and the tumor, node, and metastasis (TNM) stage in LUAD patients.

**Conclusions:**

In conclusion, the prognostic model based on FLncSig can alleviate the difficulty in predicting the prognosis and immunotherapy of LUAD patients. The identified FLncSig and the screened drugs exhibit potential for clinical application and provide references for the treatment of LUAD.

## 1 INTRODUCTION

Lung cancer ranks among the top five leading causes of cancer‐related fatalities worldwide, and it is also the most prevalent type of cancer and the primary cause of cancer fatalities in China.^1^ Lung cancer is typically classified into small‐cell lung cancer and non‐small cell lung cancer (NSCLC) based on cytological characteristics. NSCLC is the predominant type, accounting for approximately 85%–90% of lung cancer cases.^2^ Among NSCLC subtypes, lung adenocarcinoma (LUAD) is the most common, accounting for nearly 40% of cases.^3^ Despite advances in diagnosis and clinical treatment, the overall survival (OS) of patients with LUAD remains dismal.^4^ At present, dozens of drug targets are widely used, including EGFR, ALK, ROS1, BRAF, RET, and others.^5^, ^6^ However, their clinical availability is limited, and only a small fraction of patients benefit from them.^7^ Therefore, there is an urgent need to identify new therapeutic targets to improve the clinical efficacy of LUAD treatment. A reliable prognostic model is also necessary to guide clinical management and enhance the accuracy of targeted therapy.

Ferroptosis is a form of iron‐dependent, non‐apoptotic cell death characterized by distinct morphological, biological, and genetic features when compared to other regulated cell death mechanisms like apoptosis, necrosis, and autophagy.^8^ Ferroptosis is marked by an excess production of iron‐dependent intracellular oxygen free radicals, resulting in lipid peroxidation and accumulation, ultimately leading to cell membrane rupture.^9^ This process is intricately linked to the maintenance of internal environmental homeostasis and the development of various diseases, particularly cancer.^10^ A growing body of research has highlighted the crucial role of ferroptosis in diverse pathological processes and major diseases. Numerous genes that regulate ferroptosis have been identified, and it has been demonstrated that the immune system may, at least in part, through ferroptosis.^11^

Long non‐coding RNA (lncRNA) refers to a class of RNA molecules that are longer than 200 nucleotides, and most lncRNAs do not have the potential to encode proteins. LncRNA has been shown to play a crucial role in regulating various biological events associated with tumor initiation and development.^12^ Modulating the intracellular abundance of tumor‐associated lncRNAs is a potential therapeutic approach for cancer.^13^ The tumor microenvironment (TME) encompasses to the internal environment in which tumor cells grow and reside. The TME is heterogeneous and comprises various cell types. In vitro, the immune system can recognize tumor antigens and eliminate tumor cells. Interactions between the immune system and the TME are pivotal in either suppressing or enhancing the immune response.^14^ Therefore, the TME significantly influences tumor progression and treatment response.

To date, there have been limited studies utilizing ferroptosis‐related lncRNAs‐based gene signatures (FLncSig) for prognosis prediction in lung cancer patients. Nevertheless, investigations into molecular biomarkers are pivotal for predicting prognosis and determining personalized treatment strategies. In this study, we conducted a comprehensive analysis of lncRNAs associated with the ferroptosis gene set and constructed prognostic models based on these lncRNAs and clinical data. Additionally, we explored the correlation between these lncRNAs and the immune microenvironment in LUAD while identifying potential therapeutic agents. Our research offers substantial support and guidance for evaluating prognosis, tailoring treatment plans, and optimizing therapeutic approaches for LUAD patients. Furthermore, this study enhances our comprehension of the biological mechanisms underlying LUAD, provides potential clinical advantages for patients, and introduces new concepts for future clinical practice and drug development.

## 2 METHODS

The flowchart of this study is presented in Figure S1.

### 2.1 Construction and evaluation of a prognosis model of ferroptosis‐related lncRNAs

Initially, we downloaded both the gene expression and clinical data for LUAD from the TCGA database (https://portal.gdc.cancer.gov/). Genes associated with ferroptosis were retrieved from FerrDb (http://www.zhounan.org/ferrdb/current/).^15^ Subsequently, we utilized the “limma” package in R software to identify lncRNAs linked to ferroptosis. To identify genes significantly impacting patient survival, we performed univariate COX regression analysis. To ensure impartiality, we randomly partitioned the LUAD samples into a training cohort and a test cohort for subsequent statistical analyses. Further selection of ferroptosis‐related lncRNAs and construct a prognostic model were carried out through multivariate Cox regression analysis and LASSO Cox regression analysis. Using the previously established prognostic model, we classified patients in both the training and testing cohorts into high‐risk and low‐risk subgroups based on their median risk score. Kaplan–Meier survival curves were generated to analyze the survival time and vital status in each group using the “survival” and “survminer” R packages. Additionally, a risk heatmap of the genes used to constructing the model was generated using the “pheatmap” package.

### 2.2 Analysis and validation of the clinical independent prognostic model

The correlation between patients' clinical indicators and tumor prognosis was identified and visualized using the R packages “limma” and “ggpubr”. Subsequently, we conducted univariate COX regression analysis and multivariate COX regression analysis on the clinical data of LUAD patients and establish a clinically independent prognostic model. Additionally, we performed multivariate receiver operating characteristic (ROC) curve analysis and assessed 1‐, 3‐, and 5‐year ROC curve analysis. The concordance index (C‐index) was used to further evaluate the model's performance. To provide a more intuitive representation of the results from the prognostic model, we created a nomogram using the “regplot” and “rms” R packages. Survival analyses were conducted for each clinical subgroup of all patients using the “survival” and “survminer” R software packages.

### 2.3 Biological function and pathway analysis

We employed the “limma” R package to identify differentially expressed lncRNAs and conducted Gene Ontology (GO) and Kyoto Encyclopedia of Genes and Genomes (KEGG) enrichment analysis on them. GO and KEGG analyses were based on the DAVID database (https://david.ncifcrf.gov/). Subsequently, we utilized the R package “GSVA” to perform ssGSEA analysis on the enrichment scores of tumor immune cells in LUAD patients from the TCGA database.^16^ Thirteen immune events were obtained from published literature.^17^, ^18^ The “limma” package and “pheatmap” package were used to analyze the genetic differences in the expression of lncRNAs between the high‐risk and low‐risk subgroups of immune cell populations, and these differences were visualized as heatmaps.

### 2.4 TMB and TIDE analysis

We then used the “limma” R package to examine differences in tumor mutation burden between high‐risk and low‐risk LUAD patients. Subsequently, the “survival” and “survminer” packages were employed to conduct a comprehensive survival analysis of tumor mutation burden within the different risk groups. To assess immune evasion in the high‐risk and low‐risk subgroups, we obtained the TIDE score file from the TIDE website (http://tide.dfci.harvard.edu/).^19^ Biological immune markers and cells for TIDE analysis were sourced from the literature.^20^

### 2.5 Evaluation of chemotherapeutic drug sensitivity

To evaluate chemotherapeutic drug sensitivity, we used the “pRRophetic” package to predict the IC50 of various chemotherapeutic drugs in each patient and to identify potential therapeutic drugs for LUAD.^21^

### 2.6 Model validation of immunotherapy

IMvigor210 is a large phase 2 trial that investigates the clinical activity of atezolizumab, a PD‐L1 checkpoint inhibitor, in metastatic urothelial carcinoma.^22^ We utilized the IMvigor210 immunotherapy model to validate the predictive capacity of the LASSO regression model for lung cancer established by FLncSig and to predict patient response to immunotherapy. Clinical information and gene expression profile data for the IMvigor210 cohort were obtained from the “IMvigor210 Corebiologies” package.^23^

### 2.7 Analysis of stemness index based on mRNA expression (mRNAsi) with prognosis and clinical features

mRNAsi is an indicator that characterizes the similarity between tumor cells and stem cells.^24^ We conducted an analysis of mRNAsi using the “beeswarm”, “survival” and “survminer” packages.

For detailed methods, please refer to the “Data S1”.

### 2.8 Ethical approval and consent to participate

The work was approved by the Guangdong Medical University Ethical Committee (YS2019131). Informed consent forms are not required for patient data extracted from public databases.

## 3 RESULTS

### 3.1 Construct a prognostic model based on ferroptosis‐related lncRNAs

We initially identified 3252 lncRNAs associated with ferroptosis (Figure S2). Subsequently, we performed univariate Cox regression analysis on the entire cohort, resulting in the identification of 262 lncRNAs associated with prognosis (Table S1). Chi‐square tests confirmed no significant deviation in clinical characteristics between the two cohorts (Table S2). Further analysis in the training cohort revealed 224 lncRNAs linked to prognosis through univariate Cox regression analysis (Table S3). We refined this list to 50 lncRNAs associated with prognosis in the training cohort using LASSO Cox regression analysis (Figure S3A,B). Following that, multivariate Cox regression allowed us to select 30 FLncSig (AC018529.1, AC025741.1, AC010999.2, LINC02880, AC068228.2, AC026355.2, AC024361.1, AC026310.2, AC105036.3, AL606834.1, LINC01585, FAM66C, LINC01800, AC084819.1, LINC00623, LINC01150, LINC01638, AL031600.2, AL365181.2, AC009226.1, LINC02448, AP000346.1, C20orf197, AC084880.3, LINC01711, LINC00862, FRMD6‐AS1, SEMA6A‐AS2, AC007686.2, and LINC01312) for the construction of prognostic models (Table S4). The correlation between ferroptosis and the aforementioned lncRNAs is illustrated in Figure S4 (Red indicates a positive correlation, while blue indicates a negative correlation). Notably, PIK3CA, TUBE1, ANGPTL7, FBXW7, ZEB1, GABPB1, ATM, BACH1, MAPK8, and IREB2, which are ferroptosis‐related mRNAs, displayed positive correlations with most lncRNAs. Conversely, CHMP6, LONP1, OTUB1, YWHAE, PEBP1, UBC, RPL8, HSF1, ATF4, and KEAP1 were significantly negatively correlated with most lncRNAs.

### 3.2 Prognostic performance of ferroptosis‐related lncRNAs prognostic model

**FIGURE 1:**
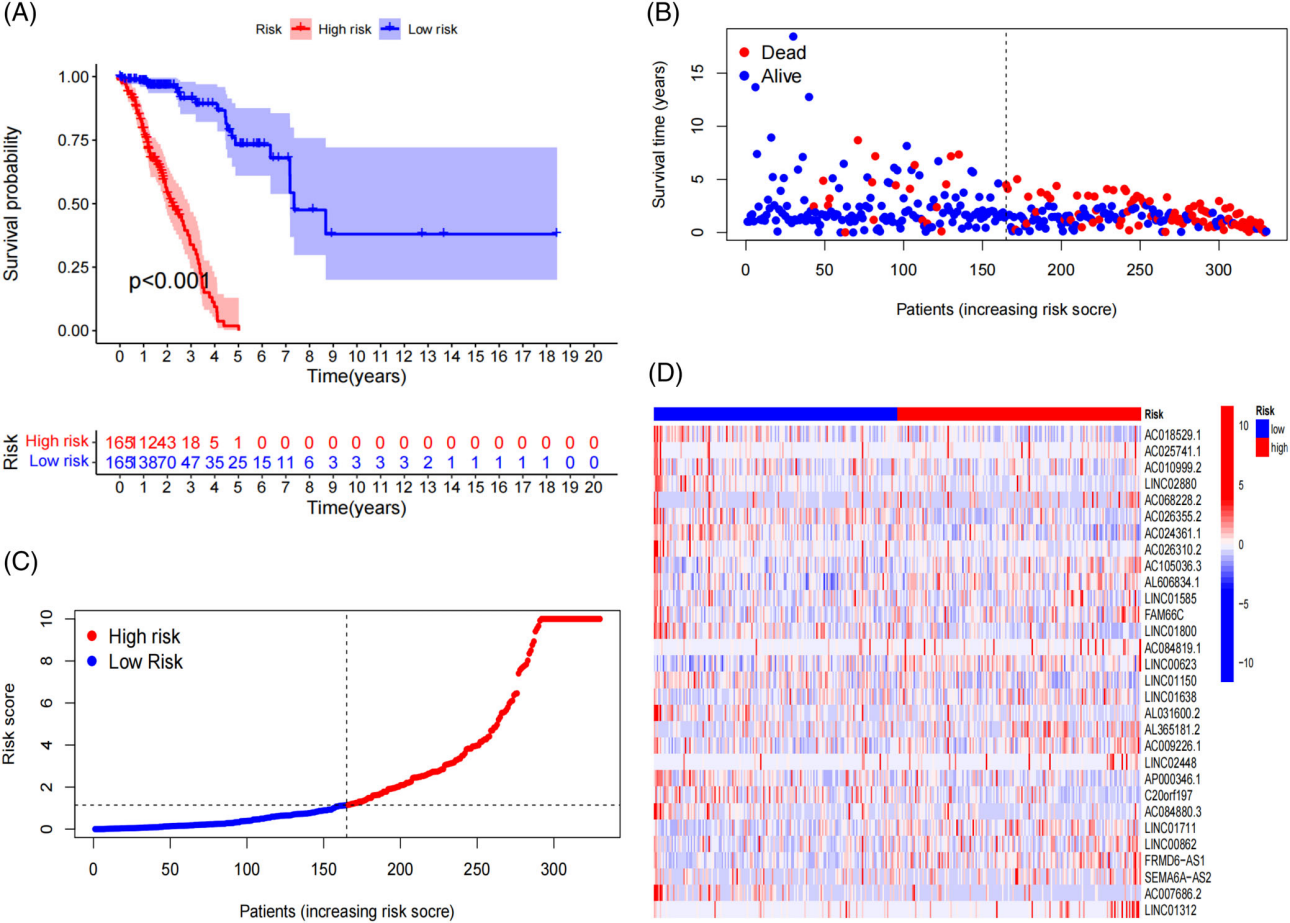
Construction of a 30‐lncRNA signature in the training cohort. (A) Kaplan–Meier curves depicting overall survival for high‐risk and low‐risk patients in the training cohort. (B) Survival status of each patient in the training cohort, with the low‐risk population on the left side of the dashed line and high‐risk population on the right side of the dashed line. (C) Patient distribution based on risk score in the training cohort. (D) Heatmap displaying the expression levels of the 30 FLncSig genes in the training cohort.

Kaplan–Meier survival curve analysis revealed that the high‐risk subgroup exhibited a significantly reduced survival times compared to the low‐risk subgroup (*p* < .001; Figures 1A, S5A,B). Risk curve analysis demonstrated that patients in the high‐risk subgroup had a higher mortality rate and shorter survival times than those in the low‐risk subgroup in the training cohort (Figure 1B,C). Similar trends were observed in the testing and entire cohorts (Figure S5C–F). Notably, the expression levels of LINC00623, AL365181.2, LINC01711, LINC00862, and FRMD6‐AS1 were significantly elevated in the high‐risk subgroup and reduced in the low‐risk subgroup. Conversely, the expression levels of AC018529.1, AC024361.1, LINC01800, AL031600.2, AP000346.1, C20orf197, and AC007686.2 were significantly higher in the low‐risk group and lower in the high‐risk group (Figures 1D, S6A,B). Furthermore, we observed that the risk score was closely associated with the survival status, tumor, node, and metastasis (TNM) stage, primary tumor (T) stage, and regional lymph nodes (N) stage (Figure S7A–D). Age, gender, race, and distant metastasis (M) stage were not significantly associated with risk scores (Figure S7E–H). These findings suggest that the model can be applied to various clinical subgroups of patients.

### 3.3 Prognostic model analysis of patient clinical information

**FIGURE 2:**
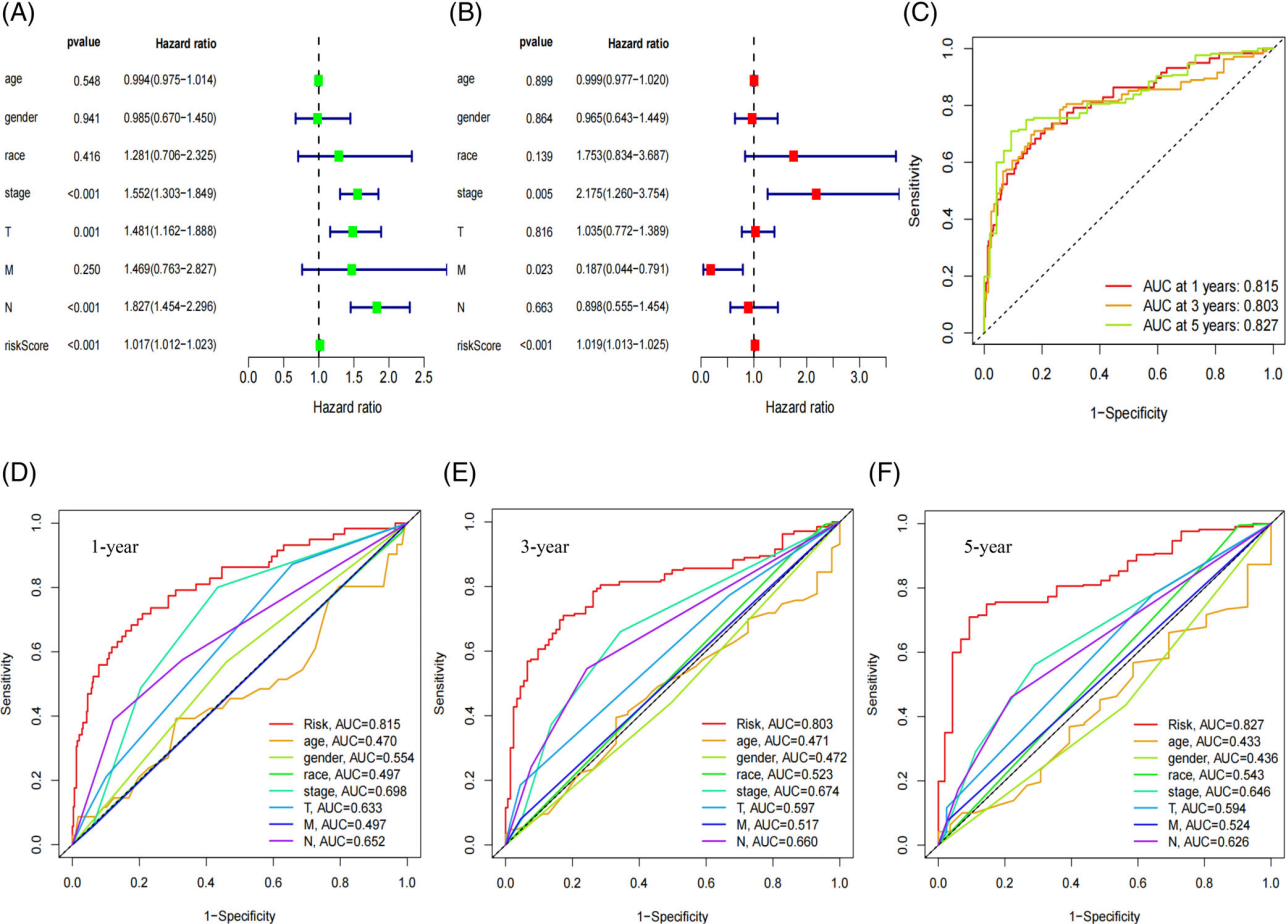
Prognostic analysis of risk score and clinicopathological factors. (A) Forest plot of univariate Cox regression analysis for prognostic factors in all patients with lung adenocarcinoma (LUAD). (B) Forest plot of multivariate Cox regression analysis for prognostic factors in all LUAD patients. (C) ROC curve analysis to assess the accuracy of the prognostic model in predicting the 1‐year, 3‐year, and 5‐year overall survival (OS) rate of patients. (D) Accuracy of the multi‐indicator ROC curve in predicting 1‐year OS rate of patients. (E) Accuracy of the multi‐indicator ROC curve in predicting 3‐year OS rate of patients. (F) Accuracy of the multi‐indicator ROC curve in predicting 5‐year OS rate of patients.

**FIGURE 3:**
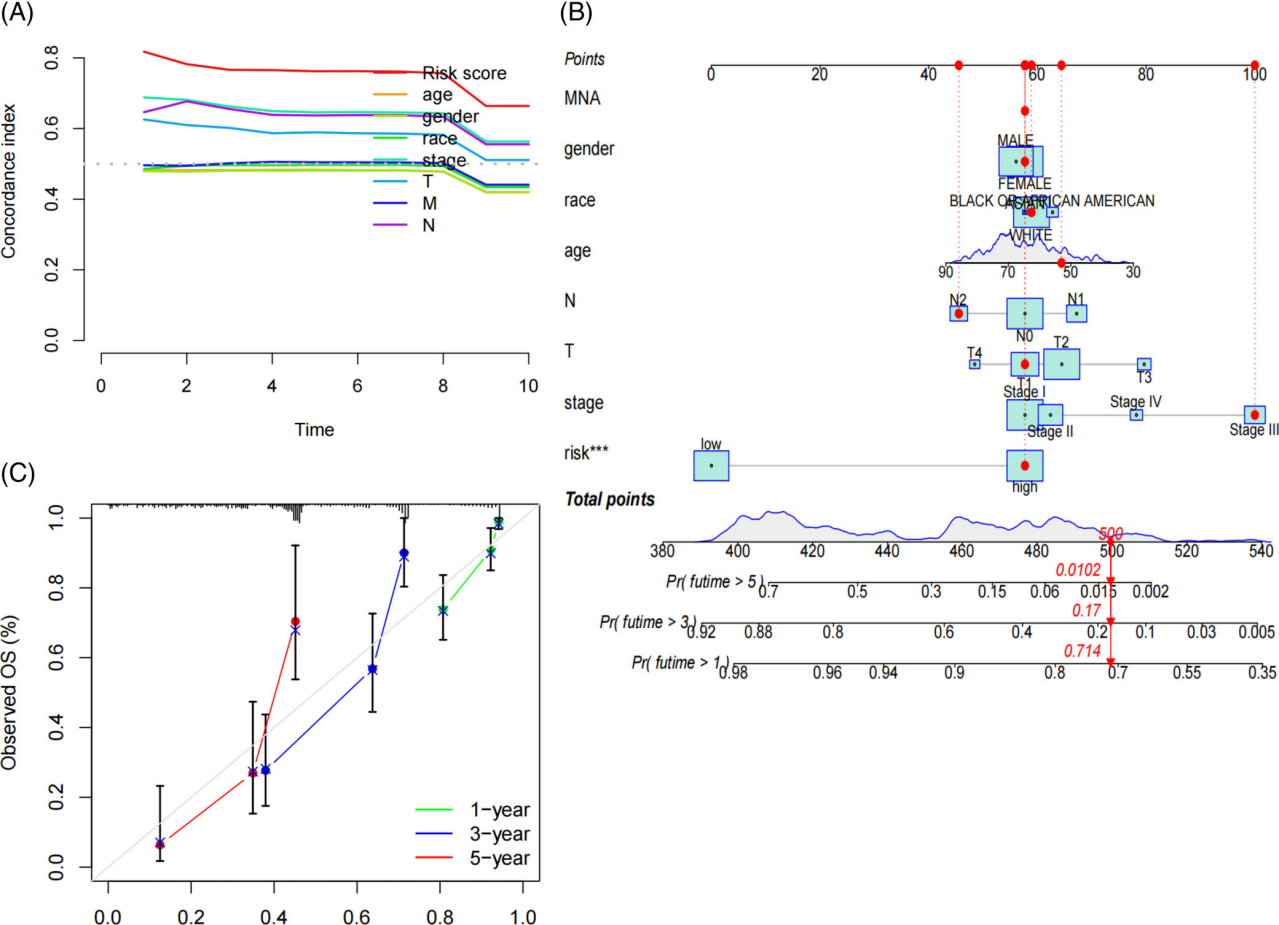
The risk score and clinicopathological factors were used to construct the prognostic model. (A) The c‐index curve was employed to evaluate the quality of the clinical independent prognostic model. (B) The nomogram was constructed based on gender, race, age, N stage, T stage, TNM stage, and risk score for the entire cohort. The vertical line from each variable value point to the axis marked “points” represents the corresponding score for the variable. The total score was calculated by summing the scores of all variables in the table. Survival rates at 1, 3, and 5 years were then determined by direct conversion of the total scores. “***” indicates a *p*‐value <.001. (C) Calibration plot of the nomogram.

Results from univariate COX analysis demonstrated that TNM stage, N stage, and risk score significantly impacted prognosis (Figure 2A). Multivariate COX analysis further confirmed that the risk score remained a significant independent prognostic factor (Figure 2B). These findings underscore the role of the risk score as an independent predictor of prognostic in LUAD patients. ROC analysis indicated that the area under the curve (AUC) for our model was 0.815 at 1 year, 0.803 at 3 years, and 0.827 at 5 years (Figure 2C), indicating that its predictive ability. Moreover, multi‐indicator ROC curve analysis demonstrated that the AUC values of risk score (AUC = 0.815), gender (AUC = 0.554), TNM stage (AUC = 0.698), T (AUC = 0.633) and N (AUC = 0.652) were statistically significant in predicting the 1‐year survival rate of LUAD patients (Figure 2D). Similar trends were observed for the 3‐ and 5‐year survival rates in Figure 2E,F. These results suggest that our LUAD prognostic model outperforms other clinical features in predicting patient survival. The C‐index curve also supported this conclusion (Figure 3A). A nomogram was constructed to predict the 1, 3, and 5‐year overall survival (OS) of patients (Figure 3B), which was well‐validated in our calibration curve (Figure 3C).

### 3.4 Validation of clinical subgroup data

Principal component analysis (PCA) revealed distinct separation between the high‐risk and low‐risk subgroups among the risk‐related lncRNAs (Figure S8A–D). Subsequently, analyses were conducted within every clinical subgroup of the entire patient cohort. In the low‐risk group, patients aged ≤65 years, patients aged >65 years, males, females, stage I, stage II, stage III, T1, T2, T3, M0, N0, N1, White, and Black or African‐American patients showed significantly better OS compared to the high‐risk group (Figure S9A–O). However, patients in stage IV, Asian, T4, N2, and M1 did not exhibit significant associations with risk scores (Figure S9P–T).

### 3.5 GO/KEGG analysis of differential lncRNAs

A total of 580 differentially expressed lncRNAs were identified between the high‐risk and low‐risk subgroups. GO enrichment analysis revealed that at least 20 lncRNAs were closely related to various pathway, including signaling receptor activator activity (GO:0030546), receptor‐ligand activity (GO:0048018), enzyme inhibitor activity (GO:0004857), collagen‐containing extracellular matrix (GO:0062023), apical plasma membrane (GO:0016324), motile cilium (GO:0031514), microtubule‐based movement (GO:0007018), cilium movement (GO:0060294), antimicrobial humoral response (GO:0019730). Notably, motile cilium (GO:0031514) was particularly associated with lung cancer (Figure S10A). A circos plot was generated to visualize representative lncRNAs and GO terms (Figure S10B). In KEGG pathway analysis, these lncRNAs were mainly linked to pathways such as metabolism of xenobiotics by cytochrome P450 (hsa00980), retinol metabolism (hsa00830), IL‐17 signaling pathway (hsa04657), drug metabolism‐cytochrome P450 (hsa00982), and amoebiasis (hsa05146) (Figure S10C). Another circos plot was created to visualize representative lncRNAs and KEGG terms (Figure S10D).

### 3.6 Immune function analysis of genes

Analysis of immune function differences in the training cohort revealed significant disparities in Type_II_IFN_response, HLA, and MHC_class_I. Type_II_IFN_response and HLA were markedly upregulated in the low‐risk subgroup and downregulated in the high‐risk subgroup, indicating they represent low‐risk pathways. Conversely, MHC_class_I was downregulated in the low‐risk subgroup and upregulated in the high‐risk subgroup, signifying it as a high‐risk pathway (Figure S11A). These immune pathway expression profiles were consistent in both the testing and entire cohorts (Figure S11B,C).

### 3.7 Differential TMB in LUAD

The findings indicated that TMB was higher in the high‐risk subgroup compared to the low‐risk subgroup in the testing cohort (*p* = .05; Figure S12A). However, there was no discernible divergence in TMB between the high‐risk and low‐risk subgroups in the training cohort (*p* = .87; Figure S12B) and the entire cohort (*p* = .32; Figure S12C). TMB survival curves for the training cohort (*p* = .13; Figure S12D), testing cohort (*p* = .068; Figure S12E), and entire cohort (*p* = .06; Figure S12F) were not statistically significant. However, in the training cohort (*p* < .001), the survival probability was higher for the H‐TMB+ low‐risk group, followed by the L‐TMB+ low‐risk group, while the H‐TMB+ low‐risk group and L‐TMB+ low‐risk group had worse survival probabilities (Figure S12G). Similar results were observed in the testing and entire cohorts (Figures S12H, S12I).

### 3.8 Immunotherapy analysis for LUAD

**FIGURE 4:**
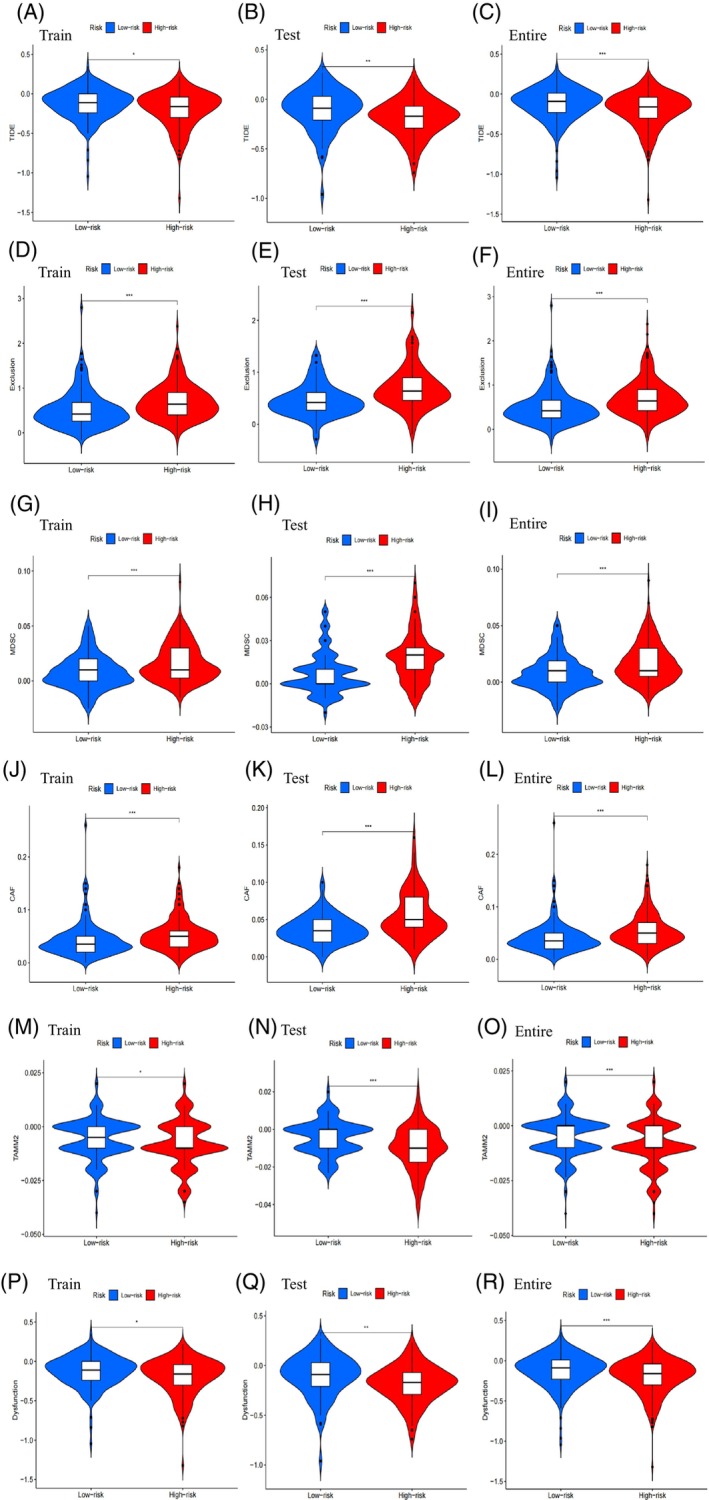
Tumor microenvironment results based on FLncSig. Differential analysis of (A‐C) Tumor Immune Dysfunction and Exclusion (TIDE), (D‐F) exclusion, (G‐I) MDSC, (J‐L) CAF, (M‐O) TAMM2, (P‐R) dysfunction in different risk subgroups in the training cohort, testing cohort, and entire cohort. *p < .05, **p < .01, ***p < .001, and ns (no statistical significance).

The low‐risk subgroup exhibited higher TIDE scores than the high‐risk subgroup, indicating a greater likelihood of immune evasion and a poorer response to immunotherapy (Figure 4A). This result was consistent in both the testing cohort (Figure 4B) and the entire cohort (Figure 4C), and was statistically significant. Exclusion (Figure 4D–F), MDSC (Figure 4G–I), CAF (Figure 4J–L), TAMM2 (Figure 4M–O), and Dysfunction (Figure 4P–R) showed statistical differences between high‐risk and low‐risk subgroups. However, the immune markers including IFNG (Figure S13A–C), MSI (Figure S13D–F), Merck18 (Figure S13G–I), CD274 (Figure S13J–L), and CD8 (Figure S13M–O) did not exhibit significant statistical differences between high‐risk and low‐risk subgroups.

### 3.9 Potential drug screening

**FIGURE 5:**
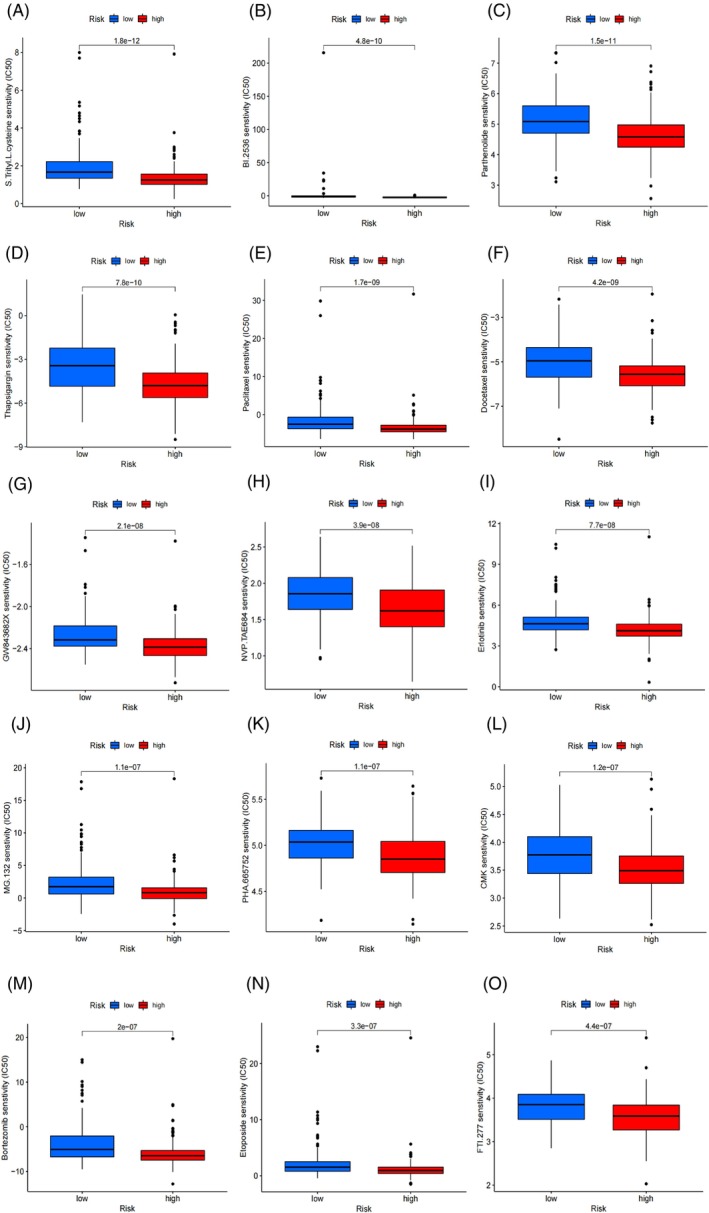
Potential Drug Screening for LUAD. The chart presents the top 15 potential LUAD drugs, including (A) S.Trityl.L.cysteine, (B) BI.2536, (C) Parthenolide, (D) Thapsigargin, (E) Paclitaxel, (F) Docetaxel, (G) GW843682X, (H) NVP.TAE684, (I) Erlotinib, (J) MG.132, (K) PHA.665752, (L) CMK, (M) Bortezomib, (N) Etoposide, and (O) FTI.277.

We screened a total of 70 drugs that exhibited significant differences in sensitivity between the high‐risk and low‐risk subgroups. In general, the high‐risk subgroups showed lower half maximal inhibitory concentrations (IC50) compared to the low‐risk subgroup, indicating higher sensitivity to most of the screened drugs. The 15 most promising candidate drugs for treating LUAD patients include S.Trityl.L.cysteine, BI.2536, Parthenolide, Thapsigargin, Paclitaxel, Docetaxel, GW843682X, NVP.TAE684, Erlotinib, MG.132, PHA.665752, CMK, Bortezomib, Etoposide, and FTI.277 (Figure 5A–O).

### 3.10 FLncSig‐based assessment of response to immunotherapy

We compared the genes acquired from the LASSO Cox regression analysis described above. Unfortunately, the models established by FlncSig did not perform well in predicting the response of IMvigor210 (*p* = .13; Figure S14A). However, the model established by FlncSig demonstrated good performance in predicting the 1‐year survival rate (*p* = .56) and 3‐year survival rate (*p* = .52) of IMvigor210 bladder cancer patients (Figure S14B). By comparing the risk scores between the primary response group (CR/PR) and the secondary response group (SD/PD), we found that the target genes of LASSO Cox regression in lung cancer patients significantly differed among the different drugs used in bladder cancer immunotherapy (*p* = .02), with higher risk scores observed in the major response group (CR/PR) (Figure S14C).

### 3.11 Prognostic and clinical characteristics analyses of LUAD mRNAsi

**FIGURE 6:**
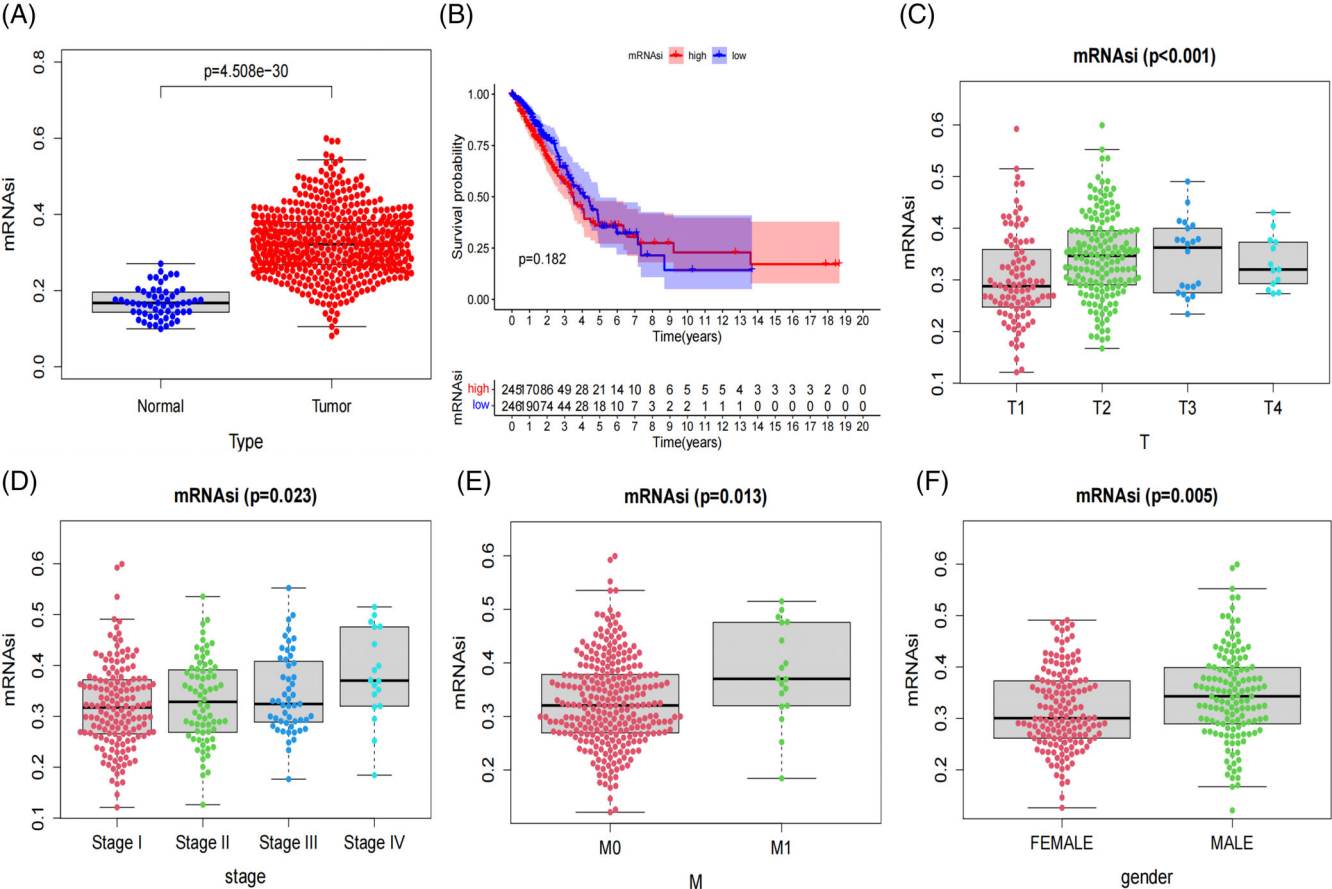
Prognostic and clinical characteristics analyses of lung adenocarcinoma stemness index (mRNAsi). (A) Comparison of mRNAsi between the lung cancer group and the normal control group. (B) Survival analysis of the high mRNAsi and low mRNAsi subgroups. (C‐F) Differences in mRNAsi between clinicopathological groups, including (C) T stage, (D) TNM stage, (E) M stage, and (F) gender. TNM refers to the tumor, node, and metastasis.

The expression level of mRNAsi in the lung cancer tissue group was significantly higher than in the normal control group (*p* < .001; Figure 6A). The 5‐year survival rate was 36.0% in the high mRNAsi group and 37.4% in the low mRNAsi group. Unfortunately, the survival probability between the high and low mRNAsi groups did not reach statistical significance (*p* = .18; Figure 6B). Additionally, we analyzed the clinical correlations of mRNAsi. The mRNAsi values of T1, T2, T3, and T4 lung cancer patients were statistically significant (*p* < .001; Figure 6C). The mRNAsi value of stage I, stage II, stage III, and stage IV lung cancer were positively correlated with TNM stage, and mRNAsi was statistically significant (*p* = .02; Figure 6D). The mRNAsi of M1 was higher than that of M0, and mRNAsi was statistically significant (*p* = .01; Figure 6E). The mRNAsi of male lung cancer patients was higher than that of female patients, and the mRNAsi was statistically significant (*p* = .005; Figure 6F).

## 4 DISCUSSION

LUAD represents a global health challenge due to its high incidence rate and poor prognosis. Early detection, precise assessment, and personalized treatment are crucial for improving the survival rates of lung cancer patients. In recent years, non‐coding RNAs have emerged as important players in various biological processes, including genetic regulation, chromosome remodeling, transcriptional regulation, and post‐translational modification.^25^, ^26^ Reliable prognostic markers based on lncRNAs have been developed for assessing the prognosis of cancer patients.^27^, ^28^ For example, ferroptosis‐related lncRNAs were used to predict the prognosis of gastric cancer.^29^ Despite the growing recognition of the roles of lncRNAs in LUAD progression, their precise impact on LUAD remains unclear. In this study, we developed a comprehensive algorithm utilizing ferroptosis‐related lncRNAs to systematically evaluate LUAD patients, encompassing prognostic features, TME, drug resistance analysis, and screening of potential therapeutic agents.

We constructed a prognostic model featuring 30 ferroptosis‐related lncRNAs. Using this model, we calculated a risk score for LUAD patients, and our results indicate that the high‐risk subgroup exhibited poorer OS. Among the 30 lncRNAs used to construct the model, the expression levels of LINC00623, AL365181.2, LINC01711, LINC00862, and FRMD6‐AS1 were significantly increased in the high‐risk group and decreased in the low‐risk group, suggesting their association with poor prognosis. In contrast, the expression levels of AC018529.1, AC024361.1, LINC01800, AL031600.2, AP000346.1, C20orf197, and AC007686.2 indicated their protective genes. LINC00623 has been reported to promote the tumorigenicity and migration of pancreatic cancer cells in vitro and in vivo.^30^ Exosomal lncRNA LINC01711 been shown to promote the metastasis of esophageal squamous cell carcinoma through the miR‐326/FSCN1 axis.^31^ These findings align with our conclusion of a poor prognosis.

In the clinical correlation analysis, we observed significant associations between survival state, TNM stage, T stage, N stage, and risk scores. We explored the relationship between clinicopathological factors and OS in LUAD patients. The FLncSig risk score emerged as an independent prognostic factor for OS. Our results demonstrated that FLncSig outperformed other clinical characteristics in predicting the prognosis of LUAD. We incorporated gender, age, race, T stage, N stage, TNM stage, and risk score into the nomogram, providing a more comprehensive tool for doctors to assess patient outcomes. Additionally, our risk assessment model proved effective across various patient subgroups, including different age groups, gender, tumor stages, and races. These findings reinforce the predictive capability of our risk assessment model. Therefore, this model holds promise for assessing patient prognosis and can guiding clinicians in making more personalized treatment decisions. In the future, it may also help doctors identify high‐risk patients early, enabling interventions to improve treatment outcomes.

The results of GO term analysis revealed that the differential expression of lncRNAs between high‐risk and low‐risk tumor populations was primarily associated with signaling receptor activator activity, receptor‐ligand activity, and enzyme inhibitor activity. This suggests that these differentially expressed lncRNAs may regulate signaling pathways related to these biological functions, potentially influencing tumor development and outcomes. Duan et al. have elucidated the synergistic effect of the peroxisome proliferator‐activated receptor gamma (PPARgamma) and nuclear factor erythrocyte‐2 related factor 2 (Nrf2) pathway in promoting the expression of relevant genes and inhibiting ferroptosis.^32^ Chen et al. have described the key molecular mechanism of ferroptosis, outlining the interplay between ferroptosis and tumor‐associated signaling pathways. Ferroptosis can be initiated through two main pathways: the exogenous or transporter‐dependent pathway and the endogenous or enzyme‐regulated pathway.^33^ Inhibition of the GPX4 enzyme has been found to directly induce ferroptosis.^34^ KEGG pathway analysis further confirmed that the differential lncRNAs associated with ferroptosis were related to metabolism and the IL‐17 signaling pathway. Ferroptosis may contribute to hypertensive nephropathy through processes such as drug metabolism‐cytochrome P450, branched‐chain amino acid (BCAA) metabolism, retinol metabolism, organic and amino acid metabolism, humoral immunity, and more.^35^, ^36^ It has been reported that ferroptosis‐related genes may impact the prognosis of tongue squamous cell carcinoma (TSCC) through the IL‐17 signaling pathway.^37^ The regulation of ferroptosis‐related enzymes, metabolism, and associated signaling pathways offers promising perspectives for the development of novel anticancer therapeutics.

The risk model showed significant correlations with various immune microenvironment characteristics. Low‐risk pathways, such as Type II IFN response and HLA, were consistent with findings from other studies. For example, a study on bladder cancer reported a significant reduction in Type II IFN response in the high‐risk group.^38^ Similarly, in uterine corpus endometrial carcinoma (UCEC), the low‐risk group exhibited elevated expression levels of HLA.^39^ In our analysis of tumor mutation burden (TMB), we did not observe significant differences between the high‐risk and low‐risk subgroups. When exploring the immune status of LUAD patients using the TIDE score, we found that patients in the high‐risk subgroup had lower TIDE scores, indicating reduced immune evasion and a potentially better response to immunotherapy.^40^, ^41^ We also observed positive correlations between risk scores and the expression of Exclusion, MDSC, CAF, and TAMM2, while Dysfunction expression was negatively correlated with risk scores. Previous studies have also shown significant correlations between MDSC, Treg, M2, and CAF with the expression of cancer susceptibility 1 (CASC1) in various tumors,^42^, ^43^ supporting our findings.

Among the 15 drugs screened by our model, Paclitaxel, Docetaxel, and Erlotinib have been proven to be beneficial for NSCLC patients.^44^, ^45^ While there may not be clear reports demonstrating the benefits of the other screened drugs in the treatment of LUAD patients, they hold potential as viable therapeutic agents for LUAD patients in a clinical setting. For example, MG.132, a proteasome inhibitor, plays a significant role in cancer research by inhibiting proteasome function, preventing the degradation of specific proteins within cancer cells.^46^

In our analysis of the IMvigor210 bladder cancer dataset, we did not observe statistically significant differences in target gene expression between the high‐risk and low‐risk subgroups. However, this lack of significance does not necessarily imply that these genes do not play important roles in bladder cancer development and prognosis. Other factors, such as tumor heterogeneity or sample size limitations, could have masked potential differences. Regarding the prognostic analysis of the lung cancer stemness index, we found that mRNAsi expression levels were significantly upregulated in the lung cancer tissue compared to the normal controls. As clinical features and TNM stage aggressiveness increased, mRNAsi scores also showed a gradual increase. Tumor stemness has been associated with cancer metastasis, drug resistance, recurrence, and poor prognosis.^47^ Additionally, the lack of statistically significant difference in OS between the high mRNAsi group and the low mRNAsi group may suggest that tumor progression alone may not be the sole prognostic factor. Tumor tissue composition, including tumor cells and other cell types, can affect the assessment of clinical features by mRNAsi and partially explain the lack of statistical significance in OS differences between the two groups.

However, our study has several limitations that should be acknowledged. Firstly, the data we utilized are sourced from TCGA data, which occasionally contains incomplete or inaccurate clinical information. Future studies with larger sample sizes and more comprehensive data integration are warranted to further validate and extend our findings. Secondly, the precise mechanism by which iron‐death‐related lncRNAs operate in LUAD remain incompletely understood. Additionally, our study is primarily based on bioinformatics analysis, and while we employed various methods to validate our results, further laboratory experiments and clinical trials are essential to confirm our findings.

In summary, this study comprehensively investigated the role of ferroptosis‐related lncRNAs and their potential applications in LUAD through a comprehensive bioinformatics analysis approach. Our findings provide novel insights and possibilities for disease diagnosis, treatment, and predicting treatment responses.

## AUTHOR CONTRIBUTIONS

**Jiaxin Dong:** Formal analysis (equal); writing – original draft (equal). **Tao Tao:** Conceptualization (equal); writing – review and editing (equal). **Jiaao Yu:** Formal analysis (supporting). **Huisi Shan:** Formal analysis (equal). **Ziyu Liu:** Writing – original draft (equal). **Guangzhao Zheng:** Formal analysis (equal). **Zhihong Li:** Formal analysis (equal). **Wanyi Situ:** Writing – review and editing (equal). **Xiao Zhu:** Conceptualization (equal); formal analysis (equal); writing – review and editing (equal). **Zesong Li:** Conceptualization (equal); funding acquisition (equal); writing – review and editing (equal).

## FUNDING INFORMATION

This work was supported partly by The National Natural Science Foundation of China (81972366); Shenzhen High‐level Hospital Construction Fund (China); The Natural Science Foundation of Guangdong, China (2022A1515012606); Shenzhen Project of Science and Technology (Grant No. KCXFZ20211020164005007, and JCYJ20220818101808018). The funders had no role in the design of the study; the collection, analysis, and interpretation of the data; the writing of the manuscript; and the decision to submit the manuscript for publication.

## CONFLICT OF INTEREST STATEMENT

The authors have stated explicitly that there are no conflicts of interest in connection with this article.

## ETHICS STATEMENT

The work was approved by the Guangdong Medical University committee (YS2021265). Informed consent forms are not required for patient data extracted from public databases.

## TRANSPARENCY STATEMENT

The lead author Jiaxin Dong affirms that this manuscript is an honest, accurate, and transparent account of the study being reported; that no important aspects of the study have been omitted; and that any discrepancies from the study as planned (and, if relevant, registered) have been explained.

## Supporting information


**Data S1.** Supporting Information.

## Data Availability

All data generated or analyzed during this study are included in this published article and its supplementary materials.
