# Supplementary material for: A ferroptosis‐related LncRNAs signature for predicting prognoses and screening potential therapeutic drugs in patients with lung adenocarcinoma: A retrospective study
Source: Cancer Rep (Hoboken). 2023 Dec 3;7(1):e1925. doi: 10.1002/cnr2.1925 (PMC10809199; doi:10.1002/cnr2.1925)
Supplement: Supplementary file 1 — Data S1. Supporting Information. [file CNR2-7-e1925-s001.docx]

**A ferroptosis-related LncRNAs signature for predicting prognoses and screening potential therapeutic drugs in patients with lung adenocarcinoma: A retrospective study**

**Abbreviations……………………………………………………………......………………………2**

**Materials and Methods…………………………………...…………….......………………………3**

Figure S1…………………………………………………………………………………………….11

Figure S2…………………………………………………………………………………………….11

Figure S3…………………………………………………………………………………………….12

Figure S4…………………………………………………………………………………………….13

Figure S5…………………………………………………………………………………………….14

Figure S6…………………………………………………………………………………………….15

Figure S7…………………………………………………………………………………………….16

Figure S8…………………………………………………………………………………………….17

Figure S9…………………………………………………………………………………………….18

Figure S10……………………………………………………………………………………......….20

Figure S11…………………………………………………………………………………………...21

Figure S12…………………………………………………………………………………………...22

Figure S13…………………………………………………………………………………………...23

Figure S14…………………………………………………………………………………………...25

Table S1…………………...………………………………………………………………………...26

Table S2…………………...………………………………………………………………………...32

Table S3…………………...………………………………………………………………………...33

Table S4…………………...………………………………………………………………………...38

**Abbreviations**

APC: antigen-presenting cell; AUC: area under the curve; BCAA: branched-chain amino acid; BP: biological process; CAF: cancer-associated fibroblasts; CASC1: cancer susceptibility 1; CC: cell component; CCR: CC chemokine receptor; C-index: concordance index; CR: complete response; DAVID: Database for Annotation, Visualization and Integrated Discovery; EGFR-TKI: epidermal growth factor receptor tyrosine kinase inhibitor; FDR: false discovery rate; FLncSig: ferroptosis-related lncRNAs-based gene signatures; GO: Gene Ontology; GSEA: gene set analysis; HLA: human leukocyte antigen; IC50: half maximal inhibitory concentration; ICTs: immune checkpoint therapy; KEGG: Kyoto Encyclopedia of Genes and Genomes; LASSO: least absolute shrinkage and selection operator; LncRNA: Long non-coding RNA; lncRNA: long non-coding RNA; LUAD: lung adenocarcinoma; M: distant metastasis; MDSC: Myeloid-derived suppressor cells; MF: molecular function; mRNAsi: stemness index based on mRNA expression; MSI: Microsatellite instability; Nrf2: nuclear factor erythrocyte-2 related factor 2; NSCLC: non-small cell lung cancer; OS: overall survival; PCA: principal components analysis; PD: progressive disease; PD-L1: programmed cell death ligand 1; PPARgamma: peroxisome proliferator-activated receptor gamma; PR: partial response; PTL: Parthenolide; ROC: receiver operating characteristic; SD: stable disease; ssGSEA: single sample gene set enrichment analysis; T: primary tumor; TAMM2: tumor-associated macrophages; TCGA: The Cancer Genome Atlas; TG: Thapsigargin; TIDE: tumor immune dysfunction and exclusion; TMB: tumor mutation burden; TME: tumor microenvironment; TNM: tumor, node, and metastasis; TSCC: tongue squamous cell carcinoma; UCEC: uterine corpus endometrial carcinoma

**Materials and Methods**

**1. Data Collection and Screening of Ferroptosis-Related lncRNAs**

**1.1 LUAD Data Collection**

In this study, we retrieved LUAD RNA-seq transcriptome data and clinical data from the Cancer Genome Atlas (TCGA) database (<https://portal.gdc.cancer.gov/>). The data comprised 526 tumor samples and 59 normal samples. Clinical characteristics of LUAD patients, including age, gender, primary tumor (T), regional lymph nodes (N), distant metastasis (M), tumor-node-metastasis (TNM) stage, race, and survival status, were collected. Patients with incomplete information were excluded from the analysis.

**1.2 Selection of the Ferroptosis Gene Set**

FerrDb is the first manually curated database for managing and identifying ferroptosis-related markers, regulators, and associated diseases [1]. We obtained 288 genes associated with ferroptosis from FerrDb (<http://www.zhounan.org/ferrdb/current/>), comprising 108 drivers, 69 suppressors, and 111 markers. After removing 28 genes with multiple annotations, we included 259 ferroptosis-associated genes in this study.

**1.3 Screening of Ferroptosis-Related lncRNAs**

We used R software (version 4.0.3) to screen lncRNAs from the TCGA transcriptome database. The "limma" package was used to read and process gene expression data. Pearson correlation coefficients were calculated to correlate ferroptosis gene sets and lncRNAs. Ferroptosis-related lncRNAs were identified in the entire cohort based on correlation coefficients and p-values. The threshold for the correlation coefficient was set at 0.4, and the significance level threshold was 0.001.

**2. Construction of Ferroptosis-Related lncRNAs Prognostic Model**

**2.1 Data Integration and Preliminary Analysis**

We integrated the lncRNAs associated with ferroptosis with their corresponding survival data and conducted a univariate Cox regression analysis to identify genes impacting patient survival.

**2.2 Sample Grouping**

The 494 LUAD samples were randomly divided into a training cohort (330 cases) and a testing cohort (164 cases). The Chi-square test was used to assess any deviation between the two cohorts.

**2.3 Establishment of FLncSig Prognostic Model**

Univariate Cox regression analyses were performed on the training cohort. Subsequently, multivariable Cox regression analysis and the least absolute shrinkage and selection operator (LASSO) Cox regression analysis were carried out on the training cohort, with LASSO regression analysis conducted using the “glmnet” package. Ferroptosis-related lncRNAs influencing LUAD patients prognosis were selected to construct a prognostic model. The risk score formula for calculating the prognostic lncRNA signature of each patient was as follows:

*FLncSig=*$\sum_{i=1}^{n} Coef\left( i \right)\times x(i)$

Where Coef(i) and X(i) represent regression coefficients estimated by multivariate Cox regression analysis and the FLncSig expression values, respectively.

**2.4 Verify of Sample Grouping Appropriateness**

We performed statistics comparisons between clinical information from the training and testing cohorts to validate the appropriateness of the grouping. Additionally, we analyzed the correlation between ferroptosis genes and lncRNAs, presenting the results in the form of a heatmap.

**3. Evaluation and Testing of Genetic Prognostic Models**

**3.1 Survival Curve Analysis**

Patients in both the training and testing cohorts were stratified into high-risk and low-risk subgroups based on the median risk score from the prognostic model. Kaplan-Meier survival curves were utilized to assess survival differences between high-risk and low-risk subgroups in each cohort, employing the “survival” and “survminer” R packages. Samples were ranked by patient risk score, and death risk curves and survival state maps were generated separately for the training group, testing group, and the entire patient cohort.

**3.2 Visualization of FLncSig in the Heatmap**

The “pheatmap” package was employed to construct a heatmap representing the risk associated with the genes used in the model.

**3.3 Model Validation and Clinical Correlation Analysis**

To validate the prognostic value of ferroptosis-associated lncRNAs, we assessed the model's performance using the testing cohort and the entire cohort. We conducted clinical correlation analyses across all patients, utilizing the “limma” and “ggpubr” R packages to explore and visualize the relationships between various clinical indicators and tumor prognosis. Clinical relevance was determined using risk scores, with a p-value <0.05 considered statistically significant.

**4. Establishment of a Prognostic Model with Clinical Information**

**4.1 Establishment of the Clinical Independent Prognostic Model**

We conducted univariate and multivariate independent prognostic analyses on LUAD patient clinical registration data to establish a clinical independent prognostic model. A forest plot was generated to visualize the results. Clinical characteristics analyzed included patient age, sex, race, T, N, M, stage, and model risk score.

**4.2 Assessment of the Clinical Independent Prognostic Model**

Multivariate receiver operating characteristic (ROC) curve analysis was performed, evaluating the model's performance at 1-year, 3-year, and 5-year intervals. AUC value >0.5 indicated good model accuracy. Additionally, the concordance index (C-index) was utilized to assess the model's quality in predicting clinical outcomes.

**4.3 Nomogram for Survival Probability Prediction**

A nomogram was constructed to predict the probability of survival at 1, 3, and 5 years, based on the clinical COX regression model as described earlier. The nomogram is an intuitive and effective tool for displaying risk model results and can be readily applied for outcome prediction. Calibration curves at 1, 3, and 5 years were used to evaluate the nomogram's predictive accuracy.

**5. Validation of Clinical Subgrouped Data**

We performed validation analyses for each clinical subgroup of all patients.

**5.1 Clinical Subgroup Stratification**

Risk scores were computed for each clinical subgroup, and these subgroups were further divided into high-risk and low-risk categories based on the median risk score. Principal components analysis (PCA) was employed to determine the discriminative potential of coding genes, noncoding genes, and all genes (mRNAs and lncRNAs) within each clinical subgroups.

**5.2 Survival Analysis of High-Risk and Low-Risk Subgroups**

The “survival” and “survminer” R packages were used to analyze survival differences between high-risk and low-risk subgroups within each clinical subgroup. P-values were calculated to assess the significance of survival differences for each clinical variable, and survival curves were generated to validate the accuracy of r the clinical independent prognosis model.

**6. Gene Ontology (GO) and Kyoto Encyclopedia of Genes and Genomes (KEGG)** **Enrichment Analyses**

**6.1 Data Source and lncRNA Data Screening**

The lncRNA dataset was obtained from the TCGA LUAD database. Samples with missing or corrupted genomic data, incomplete clinical information, non-lung adenocarcinoma cases, low-quality samples, and unclear survival time were excluded. Differential expression analysis of lncRNA genes was conducted using the “limma” R package in all patients (526 patients), and differentially expressed genes between high-risk and low-risk subgroups in LUAD samples were identified. A correlation filter of 0.4 and a p-value filter of 0.001 were applied.

**6.2 GO Enrichment Analysis**

To determine the biological functions of these genes, we performed GO enrichment analysis of differential lncRNAs using the "clusterProfiler" package in R software. GO terms were categorized into three parts: cell component (CC), molecular function (MF), and biological process (BP).

**6.3 KEGG Enrichment Analysis**

KEGG signaling pathway analysis of these differentially expressed genes was conducted using the "clusterProfiler" package. The KEGG database provides comprehensive analysis, annotation, and visualization of gene function. The p-values obtained from both analyses were corrected using false discovery rate (FDR), with a threshold of FDR≤0.05. Both GO and KEGG analyses were based on the Database for Annotation, Visualization, and Integrated Discovery (DAVID) (<https://david.ncifcrf.gov/>).

**7. Gene Enrichment Analysis and Tumor Immune Function Analysis**

Single sample gene set enrichment analysis (ssGSEA) is an extension of a gene set enrichment analysis (GSEA), which can assess the level of immune cell infiltration in a sample based on the expression level of immune cell-specific marker genes [2].

**7.1 Source of 13 Immunological Events**

Thirteen immune events representing distinct immune states, including antigen-presenting cell (APC) co-inhibition, APC co-stimulation, CC chemokine receptor (CCR), check-point, human leukocyte antigen (HLA), Inflammation-promoting, MHC class I, Parainflammation, T cell co-inhibition, T cell co-stimulation, Type I IFN response, and Type II IFN response were obtained from the published literature [3, 4].

**7.2 Definition of High - and Low-Risk Immune Cell Subgroups**

Multivariable Cox proportional-hazards models were utilized to correlate immune cell data with patient survival time. Subsequently, patients were categorized into "high-risk" and "low-risk" subgroups based on the median risk score across all samples. Thus, "high risk" and "low risk" immune cell subgroups denote varying risk levels associated with specific immune cell subgroups concerning patient outcomes.

**7.3 ssGSEA Analysis and Gene Differential Analysis**

ssGSEA was performed to assess the enrichment fraction of tumor immune cells in LUAD patients from the TCGA dataset using the R package named “GSVA”. Additionally, genetic differential analysis of the expression of lncRNAs in high-risk and low-risk immune cell subgroups was conducted using the “limma” package, and the results were visualized with heatmaps using the “pheatmap” package.

**8. Tumor Mutation Burden (TMB) Analysis**

Tumor mutation burden (TMB) is defined as the total number of gene coding errors, gene insertions, base substitutions, or base deletions per million bases. The TMB values of LUAD samples were calculated using data from the TCGA database. We employed the “limma” R package to analyze differences in TMB among LUAD patients in high-risk and low-risk subgroups. Subsequently, all LUAD patients were categorized into high TMB and low TMB subgroups based on their TMB levels. These subgroups were further combined with high-risk and low-risk subgroups. To comprehensively assess the relationship between TMB and risk grouping, we utilized the “survival” and “survminer” packages. Theoretically, higher TMB levels suggest a greater potential for an effective response to immunotherapy in patient.

**9. Tumor Immune Dysfunction and Exclusion (TIDE)**

**9.1 Obtaining TIDE Score File**

The TIDE scoring file was acquired from the TIDE website (<http://tide.dfci.harvard.edu/>) [5], designed to analyze immune escape mechanisms in cancer, particularly melanoma and non-small cell lung cancer, and predict the efficacy of immune checkpoint inhibition therapy.

**9.2 TIDE Score for Immune Escape Assessment**

TIDE scores were used to assess immune escape in high-risk and low-risk subgroups to determine the potential responsiveness immunotherapy. Lower TIDE scores indicate a more favorable response to immunotherapy.

**9.3 Analysis of Biological Immune Markers and Cells**

The association between specific biological immune markers or cell populations and LUAD lncRNAs risk subgroups was examined. These markers and cells include IFNG, Microsatellite Instability (MSI), Merck18, CD274, CD8, Dysfunction, Exclusion, Myeloid-Derived Suppressor cells (MDSC), Cancer-Associated Fibroblasts (CAF), and Tumor-Associated Macrophages (TAMM2) [6].

**10. Evaluation of Chemotherapeutic Drug Sensitivity**

Leveraging gene expression and drug sensitivity data from various cancer cell lines, the “pRRophetic” package was employed to predict chemotherapy response based on tumor gene expression levels [7]. This analysis was conducted to predict the difference in chemotherapeutic drug sensitivity between the FLncSig high-risk subgroup and low-risk subgroup. Predictions were made for the half maximal inhibitory concentration (IC50) of various chemotherapeutic agents for each patient, facilitating the identification of potential therapeutic options for LUAD.

**11. Model Validation and Immunotherapy Response Analysis**

**11.1 Application of IMvigor210 Clinical Trial Data**

IMvigor210, a phase 2 trial exploring the clinical activity of atezolizumab in metastatic urothelial cancer, was utilized to evaluate the predictive model's performance [8]. Given its extensive follow-up duration, IMvigor210 data were employed to assess the model's effectiveness in predicting immune checkpoint therapy (ICTs) outcomes in a cancer cohort.

**11.2 Data Acquisition and Analysis**

The IMvigor210 clinical trial data was retrieved from the website (https://clinicaltrials.gov/ct2/show/NCT02108652). Clinical information and gene expression profile for the IMvigor210 cohort were obtained using the “IMvigor210 Corebiologies” package [9]. These data were then integrated with the lncRNAs identified through the previously mentioned LASSO regression analysis to employ the IMvigor210 model. The same risk-score formula was applied to compute a risk score for each patient within the IMvigor210 cohort, subsequently categorizing patients into high-risk and low-risk subgroups. Survival curve and ROC curve were generated to confirm the predictive function of the LASSO regression model established by FLncSig for lung cancer.

**11.3 Analysis of Response to Immunotherapy**

In addition, patient response to immunotherapy was predicted using the IMvigor 210 cohort. After PD-L1 treatment, samples were divided into the following categories based on patient response: Patients were categorized as complete response (CR), partial response (PR), stable disease (SD), or progressive disease (PD) based on their response to PD-L1 treatment. CR and PR indicated responders to immunotherapy, while SD and PD indicated non-responders.

**12. Prognosis and Clinical Characteristics Analysis with mRNAsi**

Stemness indices, such as mRNAsi, serve as a prognostic indicator for predicting tumor recurrence risk and guiding treatment decisions. mRNAsi reflects transcriptome stemness signature and can be used to assess the similarity between cancer cells and stem cells [10].

**12.1 Evaluation of the Impact of mRNAsi on prognosis**

We conducted a comprehensive molecular signature analysis using LUAD samples from TCGA to derive the mRNAsi index for each sample. The mRNAsi index had been previously determined in a separate study. Initially, we assessed the statistical significance of mRNAsi between the lung cancer cell group and the normal control group. The Wilcoxon rank-sun test was employed to quantify the difference between the normal and tumor groups. Subsequently, the sample were stratified into two groups based on the median mRNAsi score, which was used for prognostic evaluation through OS analysis. The Log-Rank test was utilized to determine the significance of the differences between the low-risk and higher-risk subgroups.

**12.2 Exploring the association between mRNAsi and clinical features**

Furthermore, we extracted clinical features (including age, gender, histological type, primary tumor (T), regional lymph nodes (N), distant metastasis (M), and the tumor, node, and metastasis (TNM) stage) to investigate their association with stemness indicators. The Wilcoxon rank-sum test and Kruskal-Wallis test were employed to assess statistical significance. A p-value of less than 0.05 was considered statistically significant.

1. Zhou, N. and J. Bao, *FerrDb: a manually curated resource for regulators and markers of ferroptosis and ferroptosis-disease associations.* Database (Oxford), 2020. **2020**.

2. Wang, X., et al., *A combination of ssGSEA and mass cytometry identifies immune microenvironment in muscle-invasive bladder cancer.* J Clin Lab Anal, 2021. **35**(5): p. e23754.

3. Zheng, D., Z. Wei, and W. Guo, *Identification of a Solute Carrier Family-Based Signature for Predicting Overall Survival in Osteosarcoma.* Front Genet, 2022. **13**: p. 849789.

4. Jia, C.L., F. Yang, and R. Li, *Prognostic Model Construction and Immune Microenvironment Analysis of Breast Cancer Based on Ferroptosis-Related lncRNAs.* Int J Gen Med, 2021. **14**: p. 9817-9831.

5. Fu, J., et al., *Large-scale public data reuse to model immunotherapy response and resistance.* Genome Med, 2020. **12**(1): p. 21.

6. Liu, Z., et al., *The Identification and Validation of Two Heterogenous Subtypes and a Risk Signature Based on Ferroptosis in Hepatocellular Carcinoma.* Front Oncol, 2021. **11**: p. 619242.

7. Geeleher, P., N. Cox, and R.S. Huang, *pRRophetic: an R package for prediction of clinical chemotherapeutic response from tumor gene expression levels.* PLoS One, 2014. **9**(9): p. e107468.

8. Mariathasan, S., et al., *TGFbeta attenuates tumour response to PD-L1 blockade by contributing to exclusion of T cells.* Nature, 2018. **554**(7693): p. 544-548.

9. Powles, T., et al., *MPDL3280A (anti-PD-L1) treatment leads to clinical activity in metastatic bladder cancer.* Nature, 2014. **515**(7528): p. 558-62.

10. Bai, K.H., et al., *Identification of cancer stem cell characteristics in liver hepatocellular carcinoma by WGCNA analysis of transcriptome stemness index.* Cancer Med, 2020. **9**(12): p. 4290-4298.


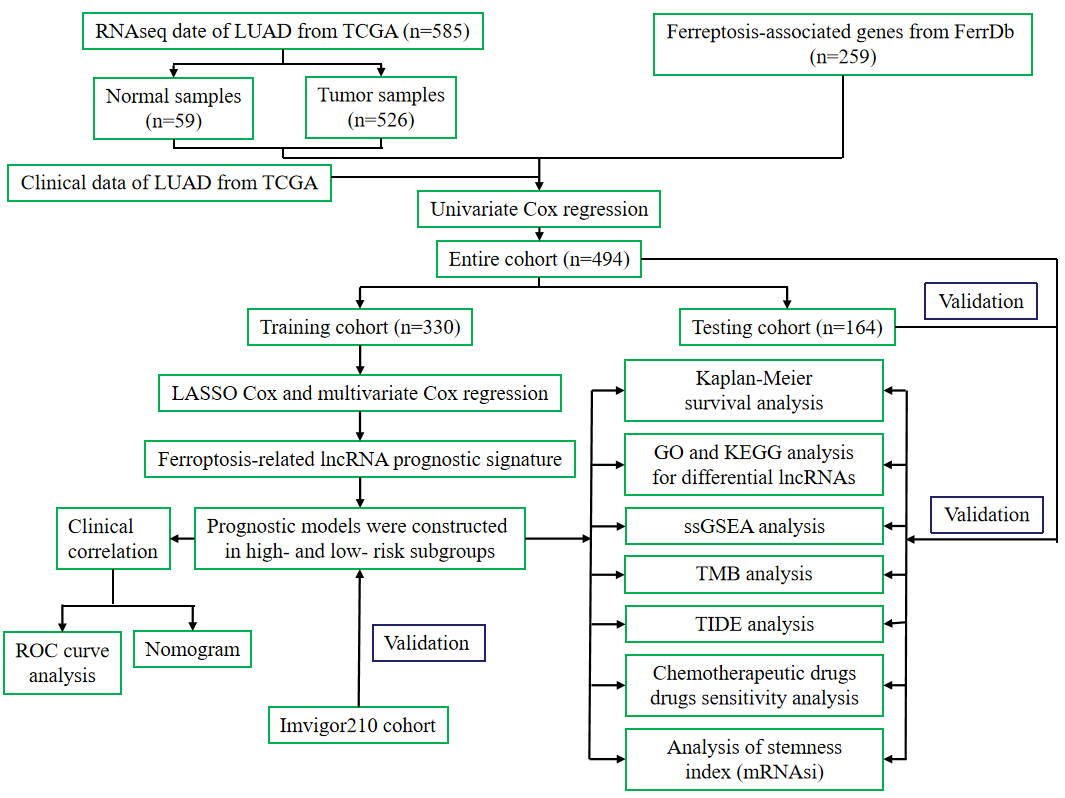
**Figure S1.** **Flowchart of Our Study.**


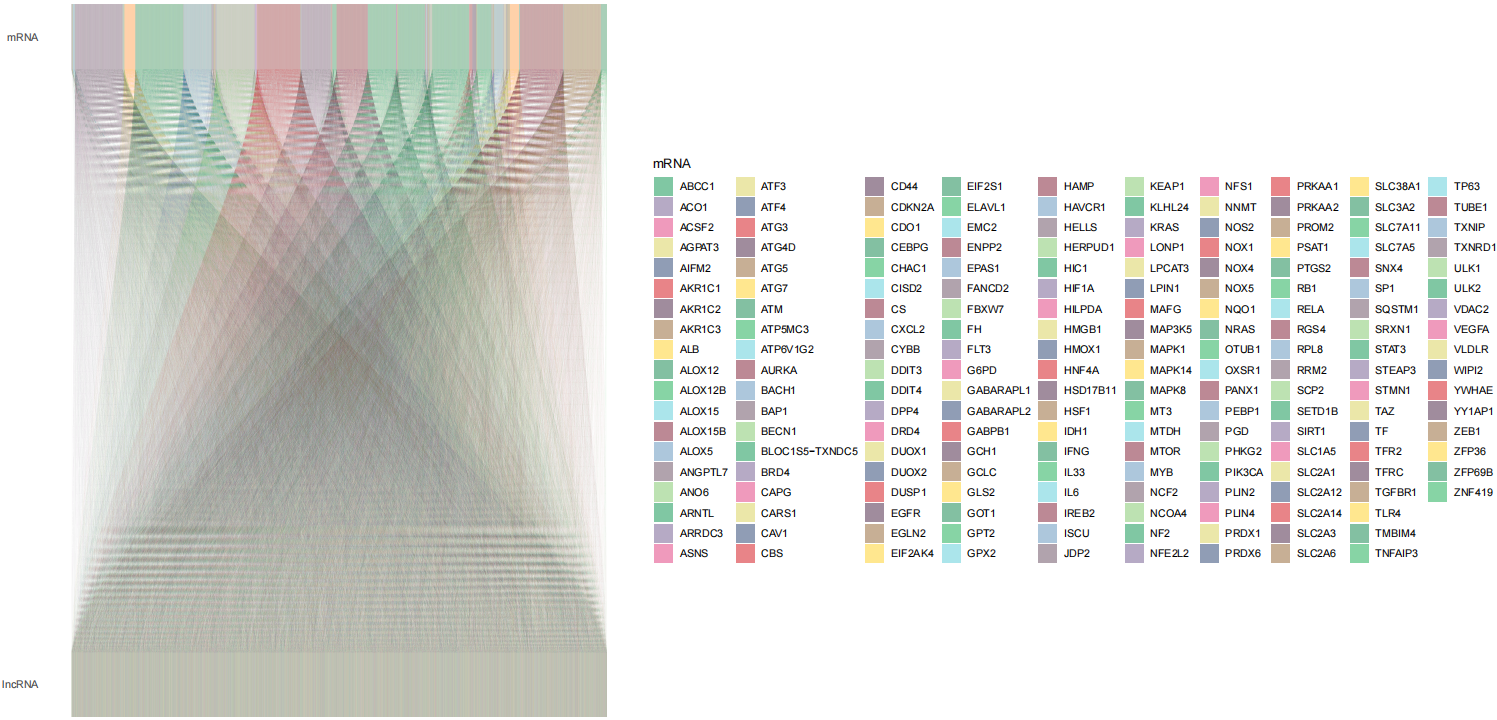


**Figure S2.** **Ferroptosis-related lncRNA, correlation＞0.4, P＜0.001.**

A B


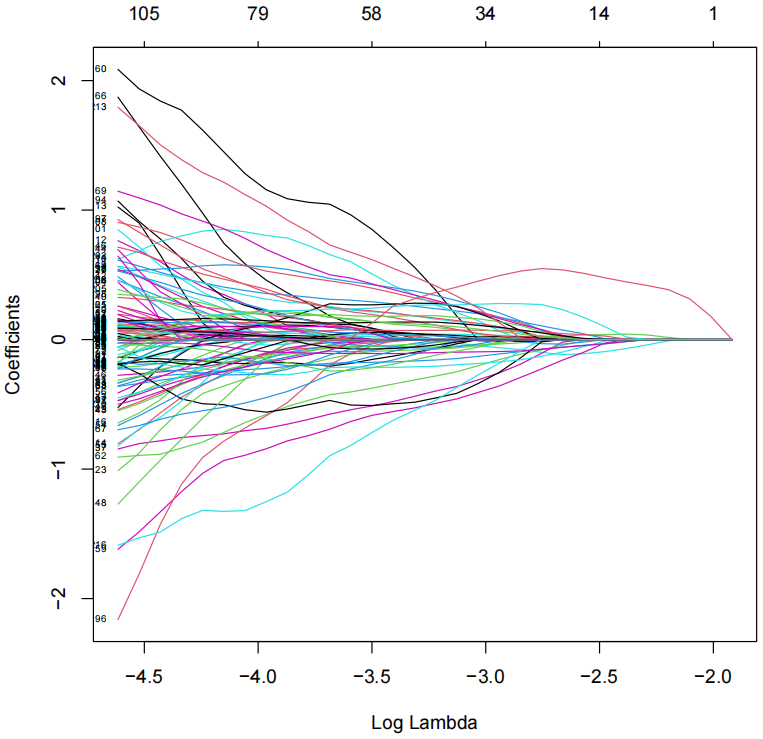

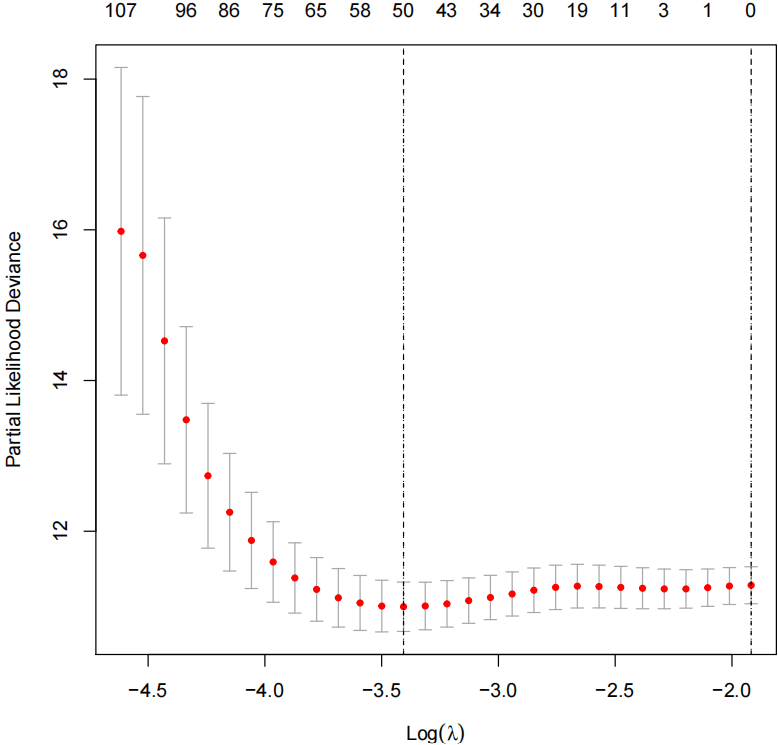


**Figure S3. Construction of Risk Signature.** (A) LASSO Cox regression analysis of prognostic ferroptosis-related lncRNAs. (B) Correlation between ferroptosis genes and lncRNAs involved in model construction.


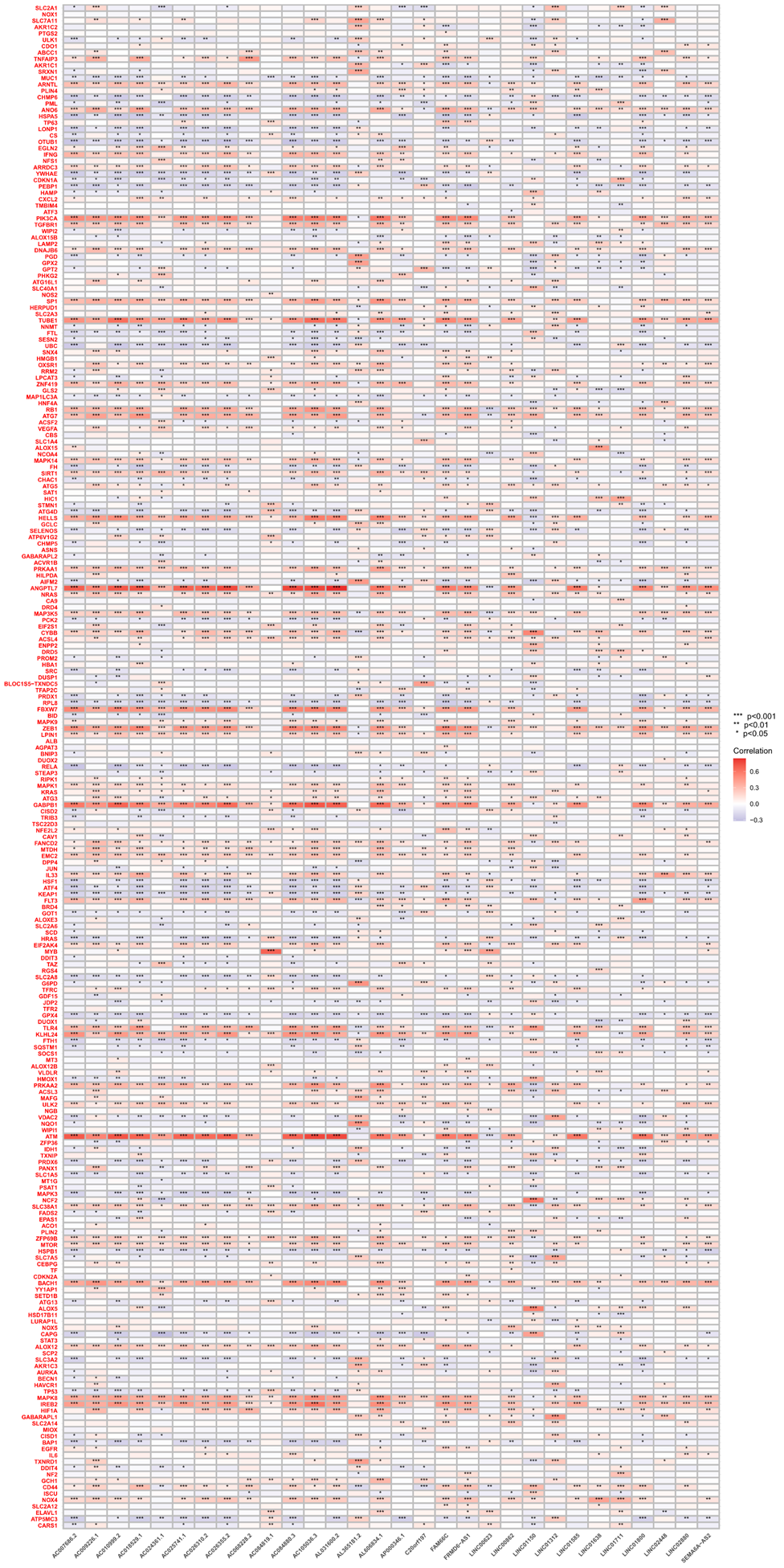


**Figure S4.** **Correlation between Ferroptosis-Associated Genes and lncRNAs Involved in Model Construction.**


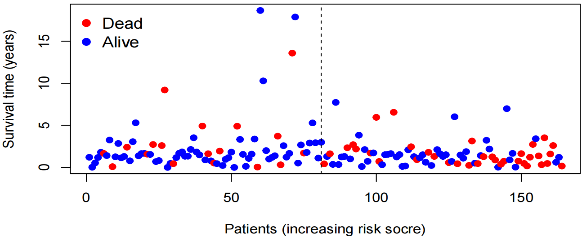

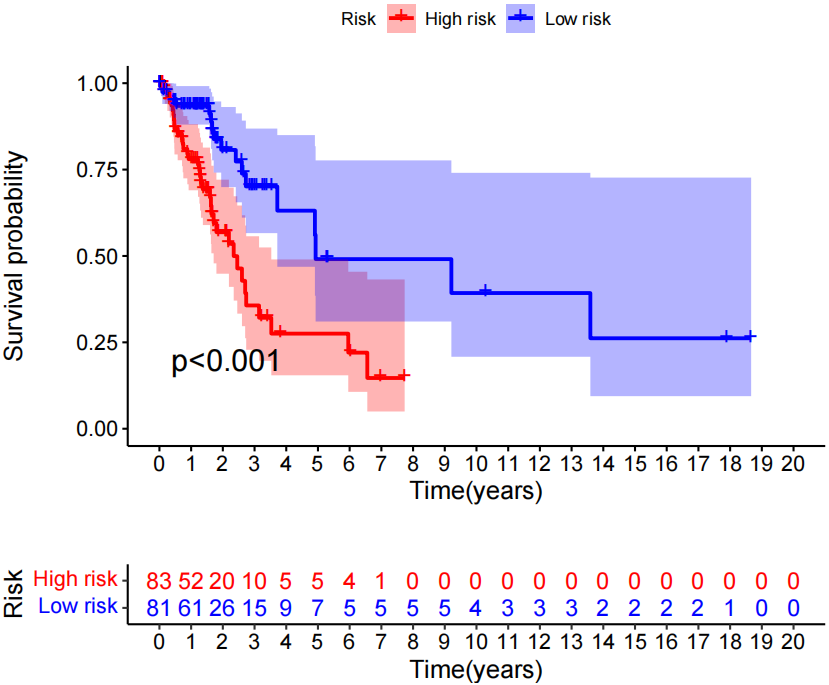
A C


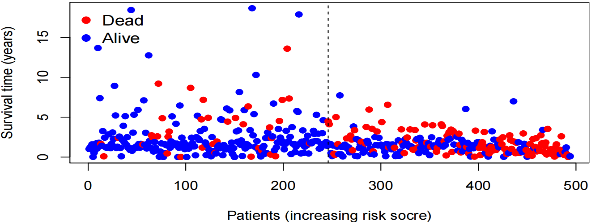
 D


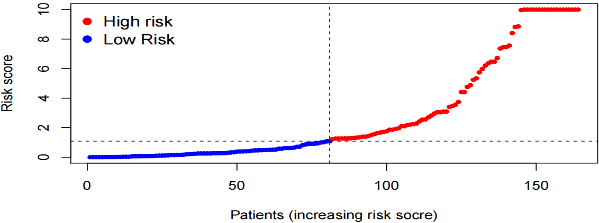

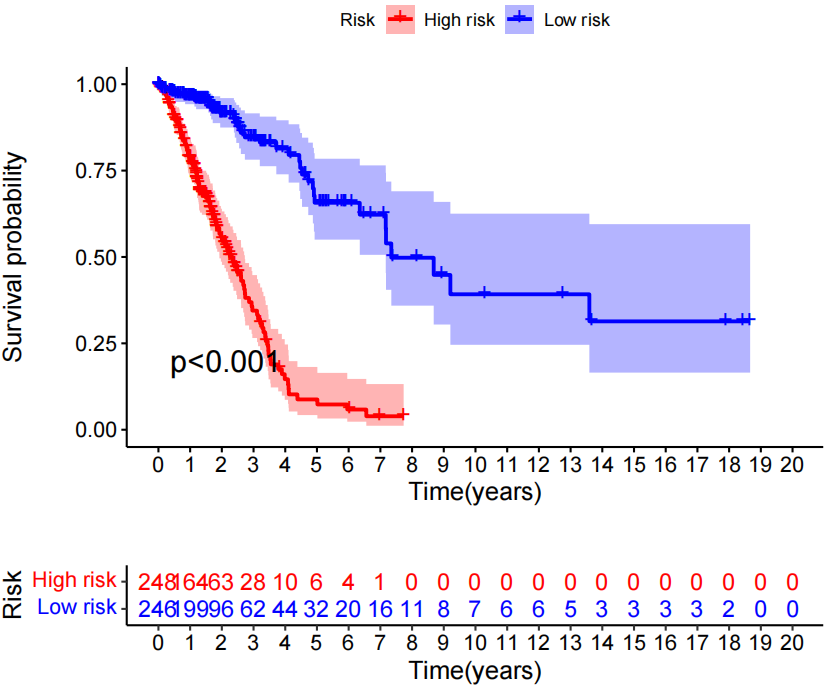
B E


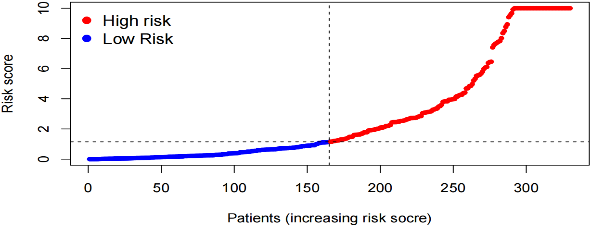
 F

**Figure S5.** **The 30 lncRNAs Characteristics Were Validated in the Testing Cohort and the Entire Cohort.** (A) Kaplan-Meier curves of overall survival for high-risk and low-risk patients in the testing cohort. (B) Kaplan-Meier curves of overall survival for high-risk and low-risk patients in the entire cohort. (C) Survival status of each patient in the testing cohort. (D) Survival status of each patient in the entire cohort. Low-risk population: left side of the dashed line; high-risk population: the right of the dashed line. (E) Patient distribution based on risk score in the testing cohort. (F) Patient distribution is based on risk score in the entire cohort.


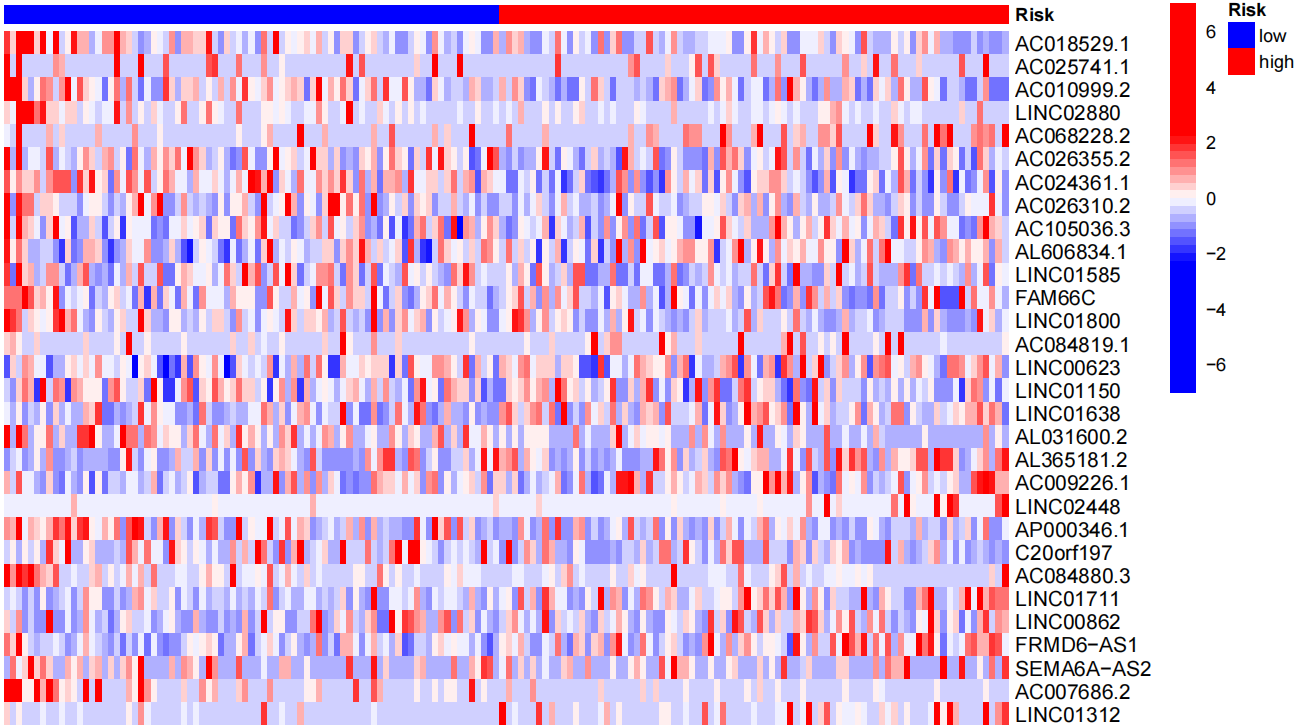
A


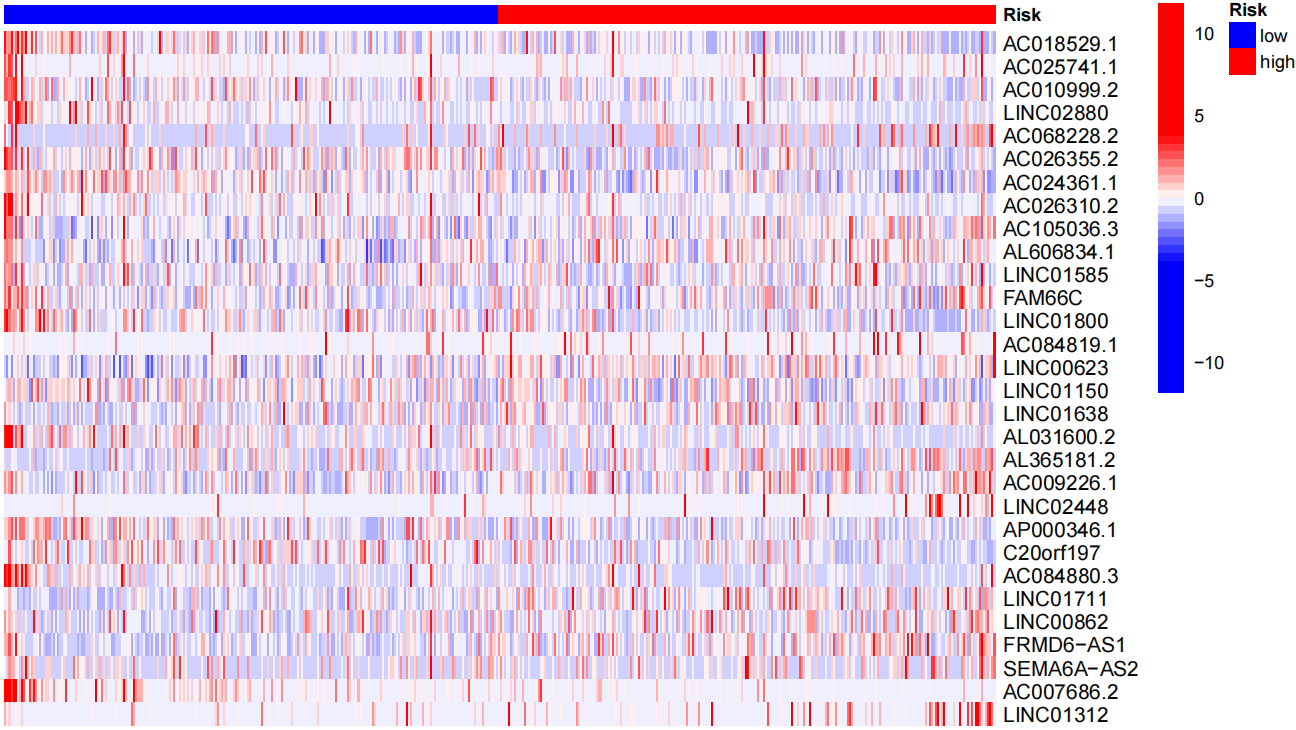
B

**Figure S6.** **The Heatmap of 30 FLncSig** (A) Testing cohort (B) Entire cohort


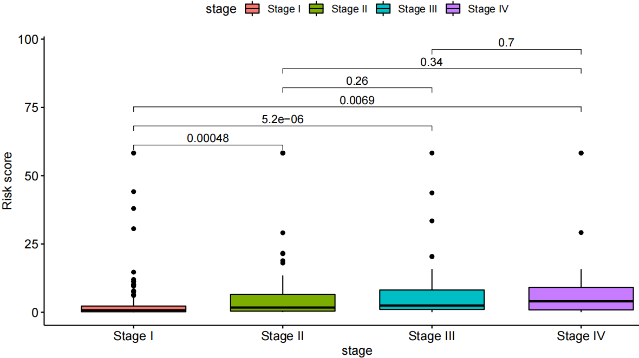

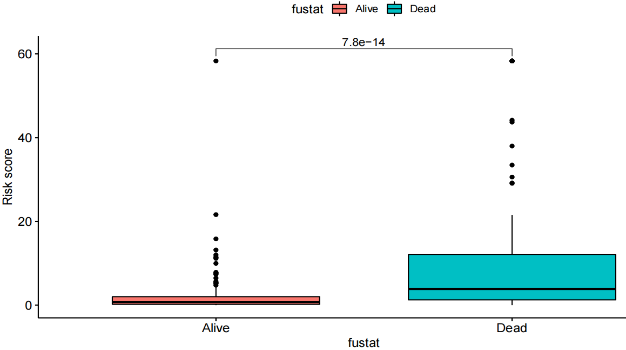
A B


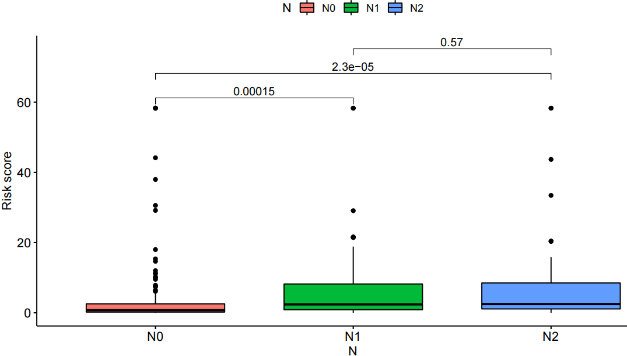

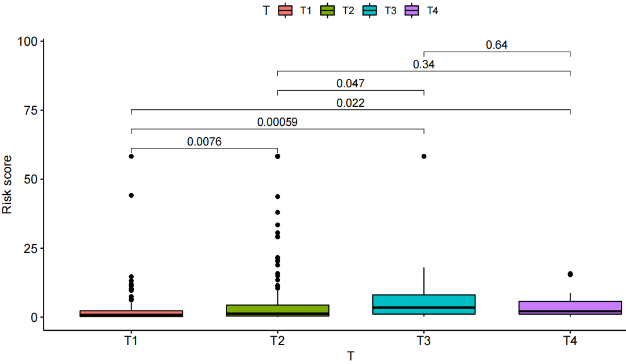
C D


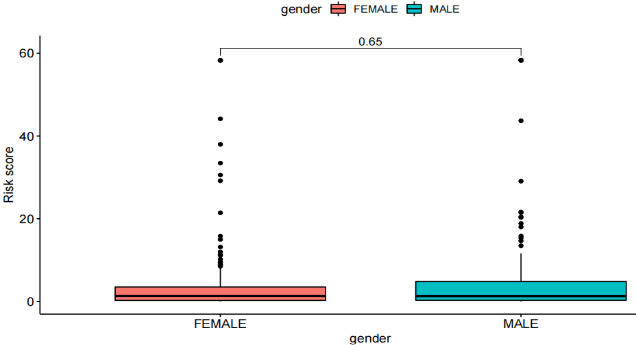

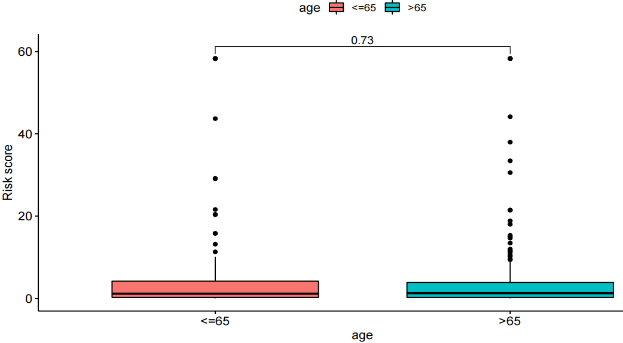
E F


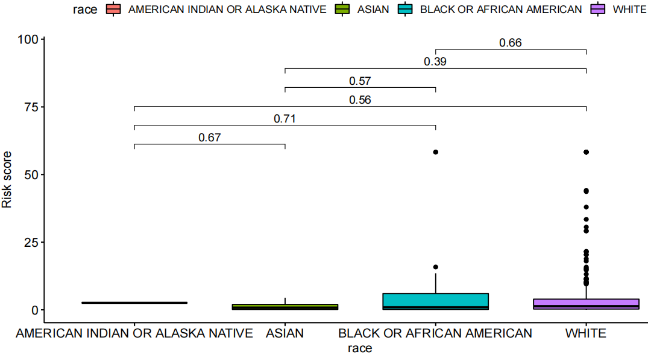

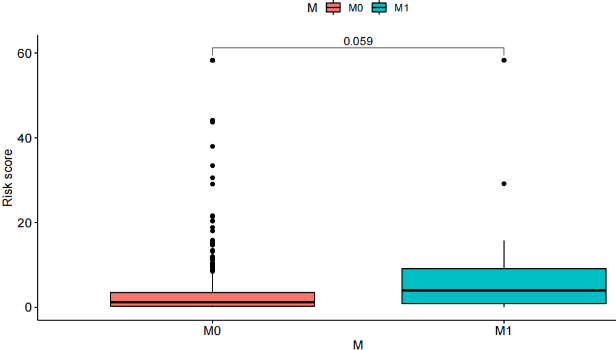
 G H

**Figure S7. Scatter Plots Show the Correlation between Clinicopathological Factors and Risk Scores.** Including: (A) fustat, (B) tumor, node, and metastasis (TNM) stage, (C) T stage, (D) N stage, (E) age, (F) gender, (G) race, and (H) M stage.


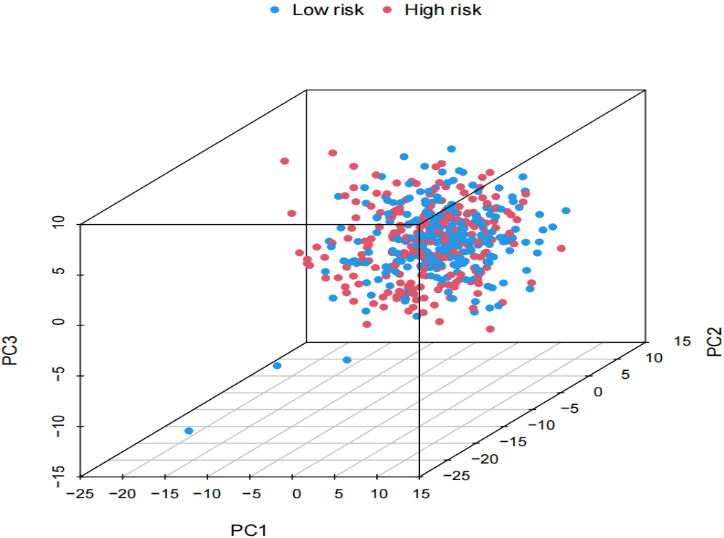

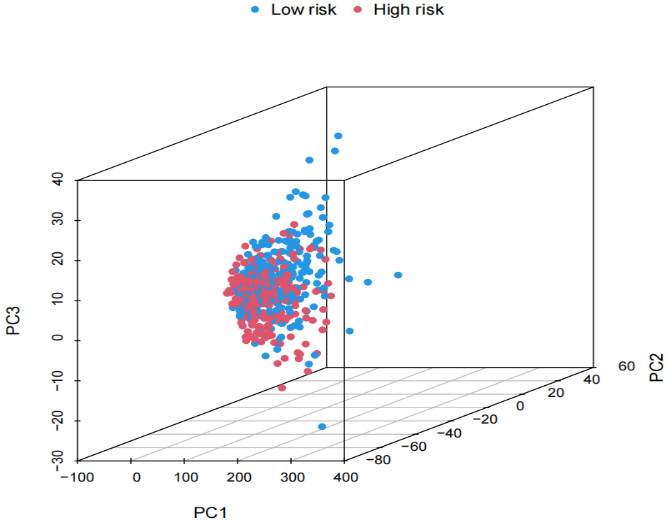
A B

mRNA lncRNA

C D


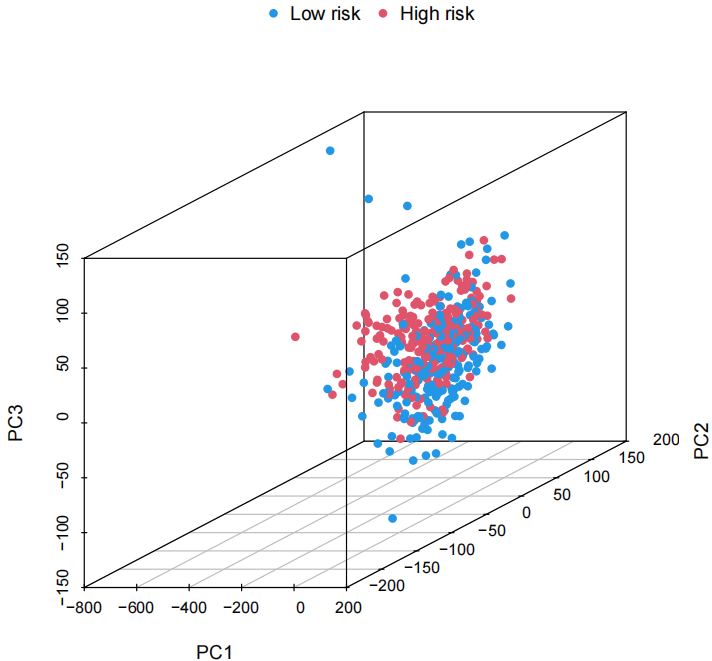

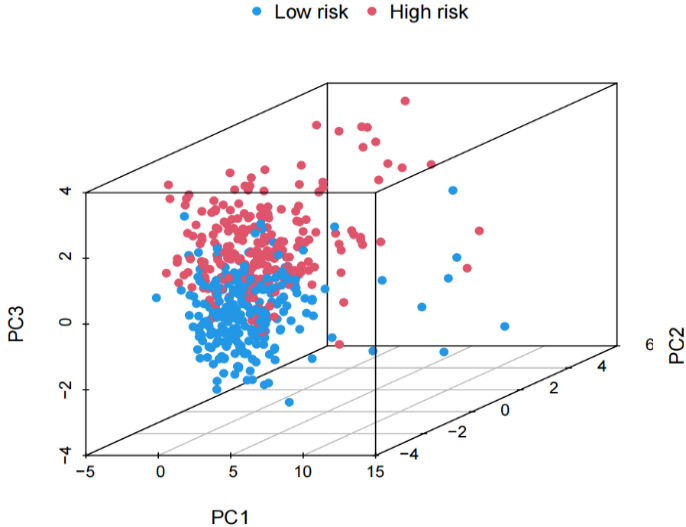


all genes risk lncRNA

**Figure S8. Principal Component Analysis (PCA).** (A) PCA analysis of ferroptosis genes (mRNAs). (B) PCA analysis of all ferroptosis-related lncRNAs. (C) PCA analysis of all genes (mRNAs and their co-expressed lncRNAs). (D) PCA analysis of ferroptosis-related lncRNAs for constructing prognostic models.

A B C


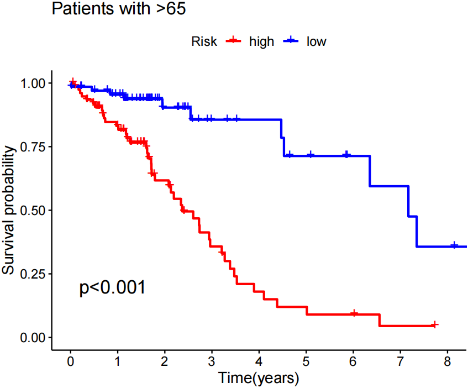

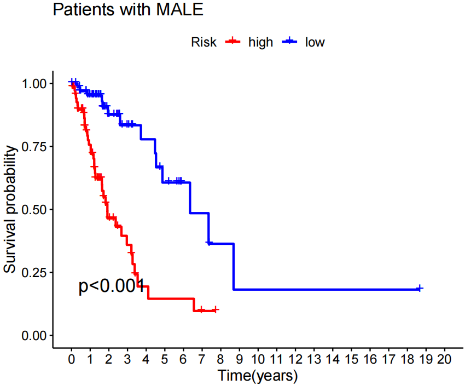

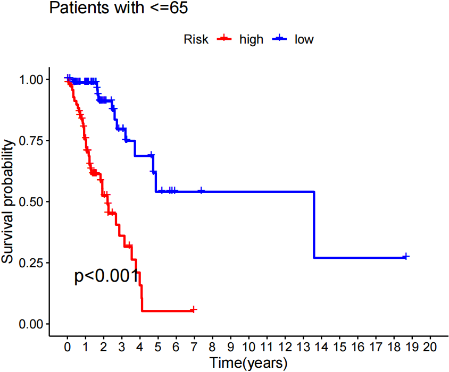


D E F


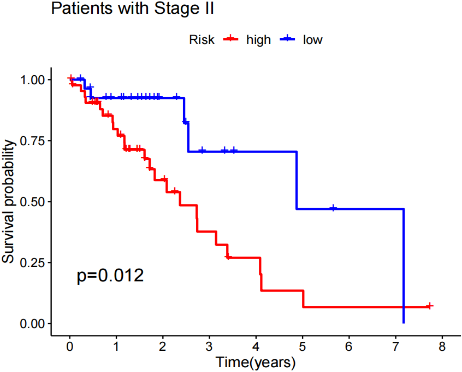

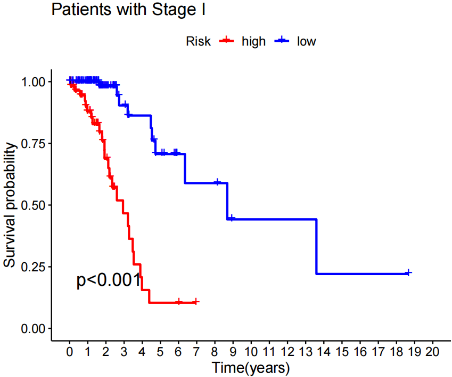

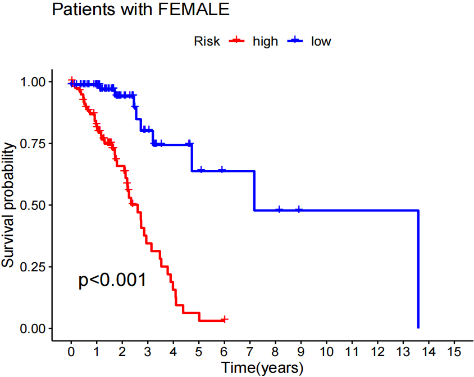


G H I


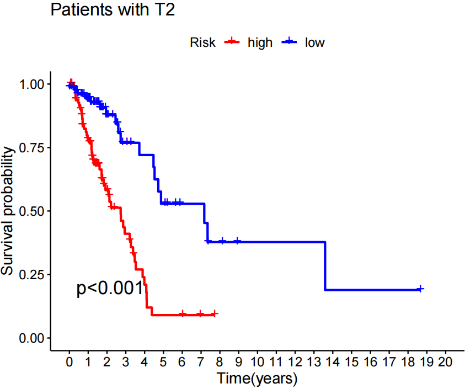

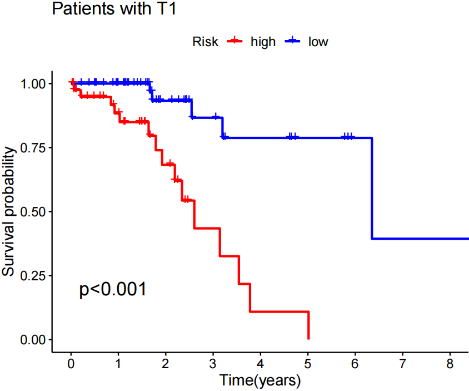

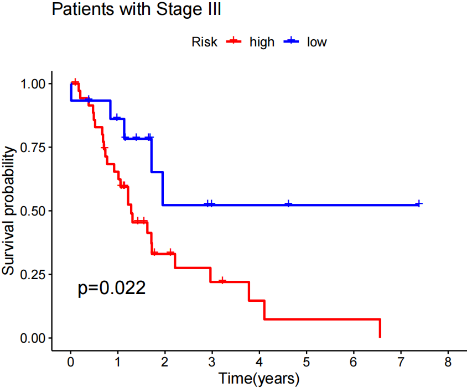


J K L
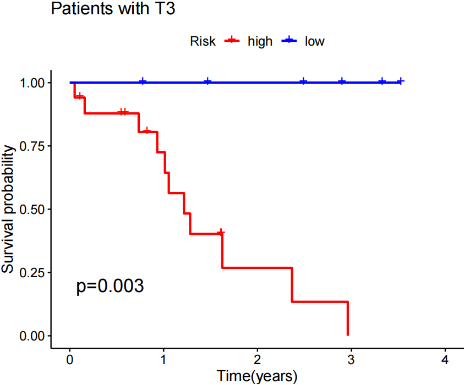


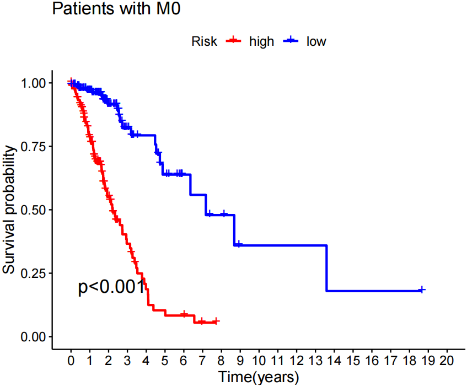

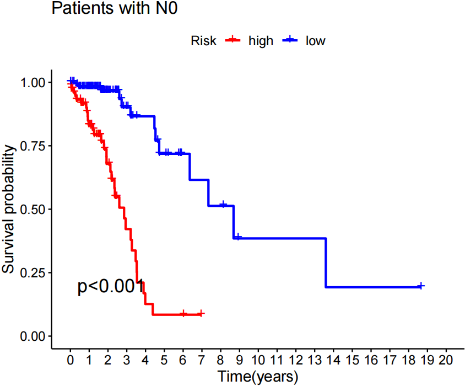


M N O


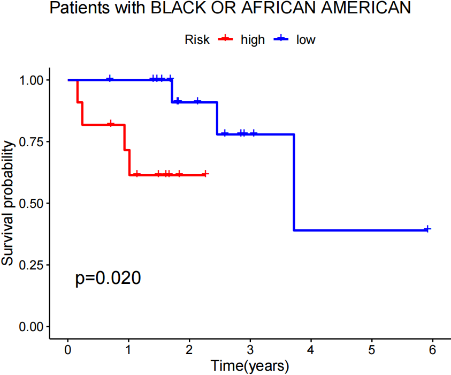

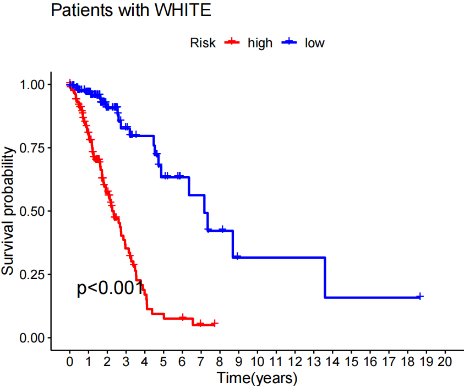

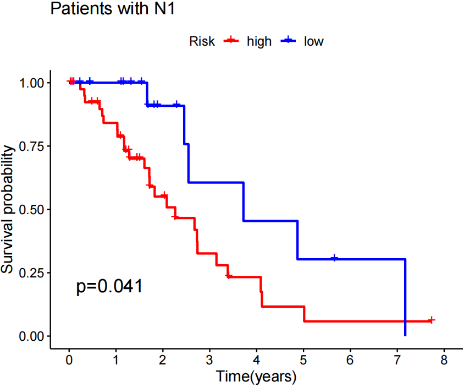


P Q R


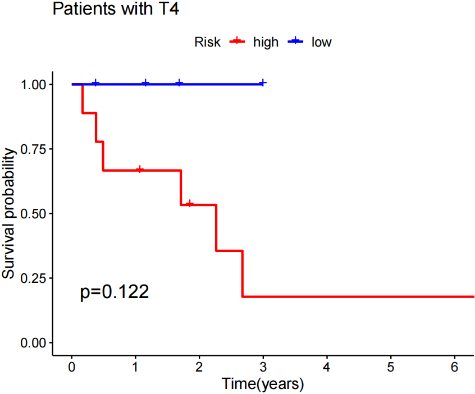

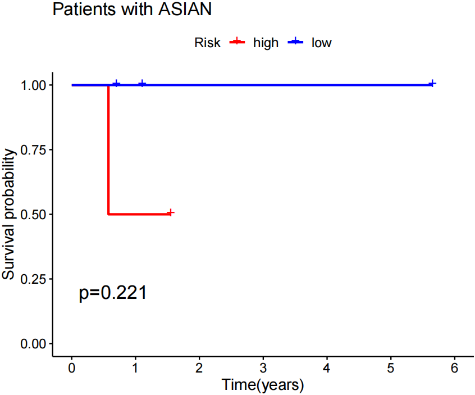

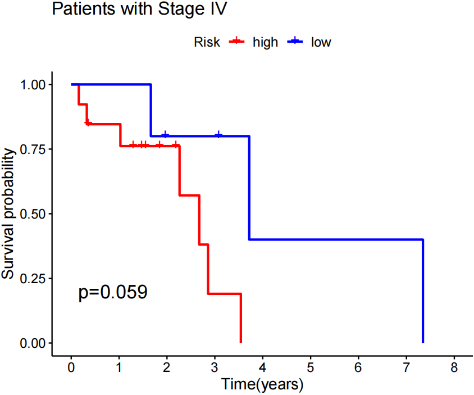


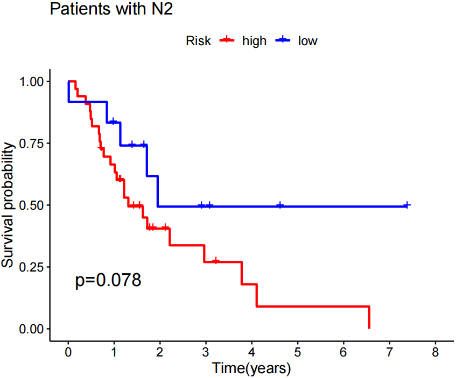

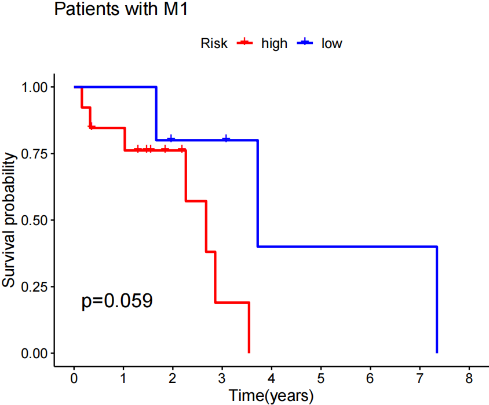


S T

**Figure S9. Stratification Analysis of Survival Based on Clinicopathological Features of the Prognostic Model.** Survival analysis of all lung adenocarcinoma (LUAD) patients adjusted to (A and B) age, (C and D) gender, (E-G) pathological stage Ⅰ-Ⅲ, (H-J) T1-T3 stage, (K) M0, (L and N) N0-N1 stage, (N) race-white, (O) Race-Black or African American, (P) stage Ⅳ, (Q) race-Asian, (R) T4, (S) N2, (T) M1 between high-risk and low-risk subgroups.

A B


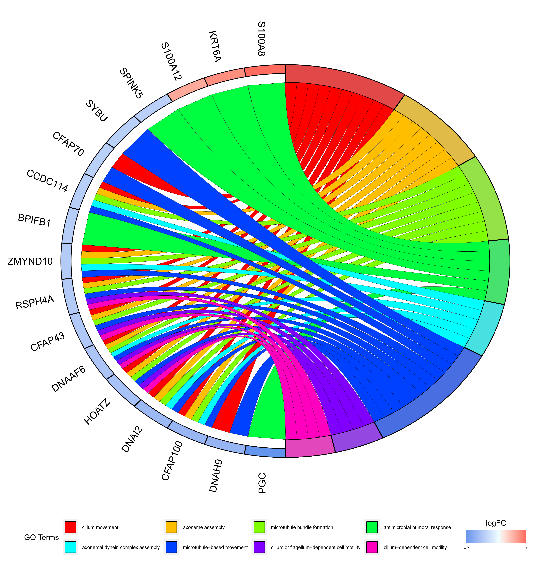

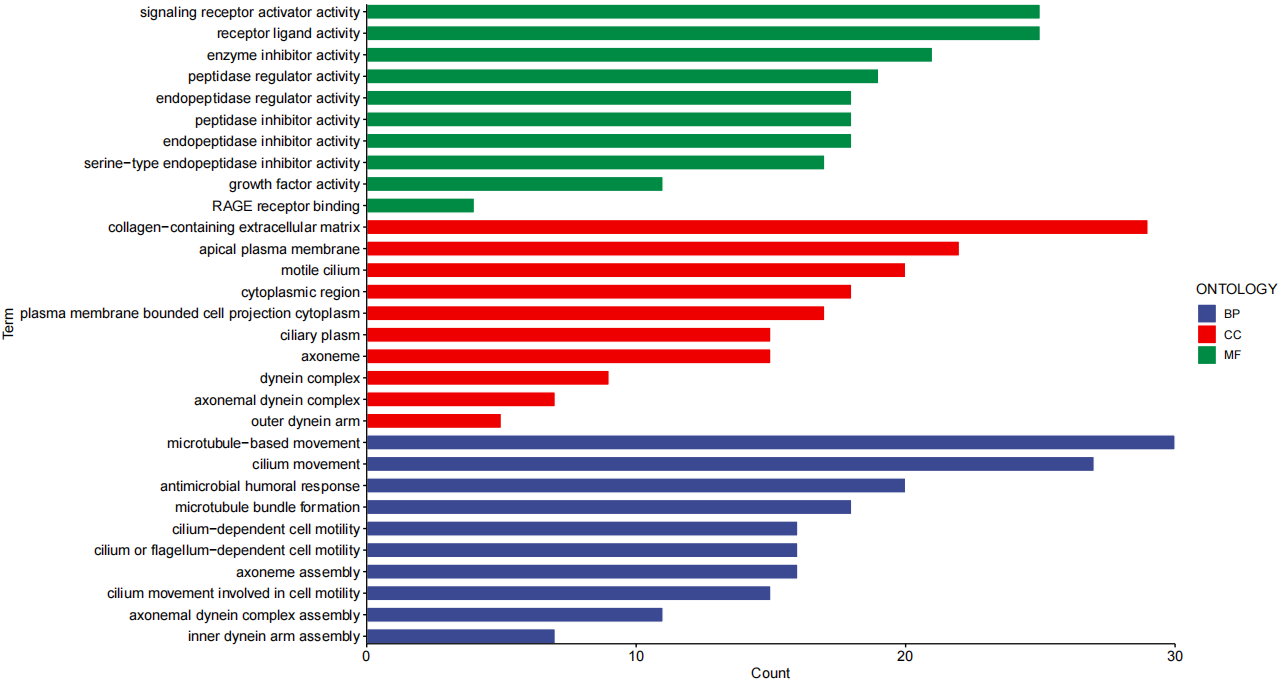


C D


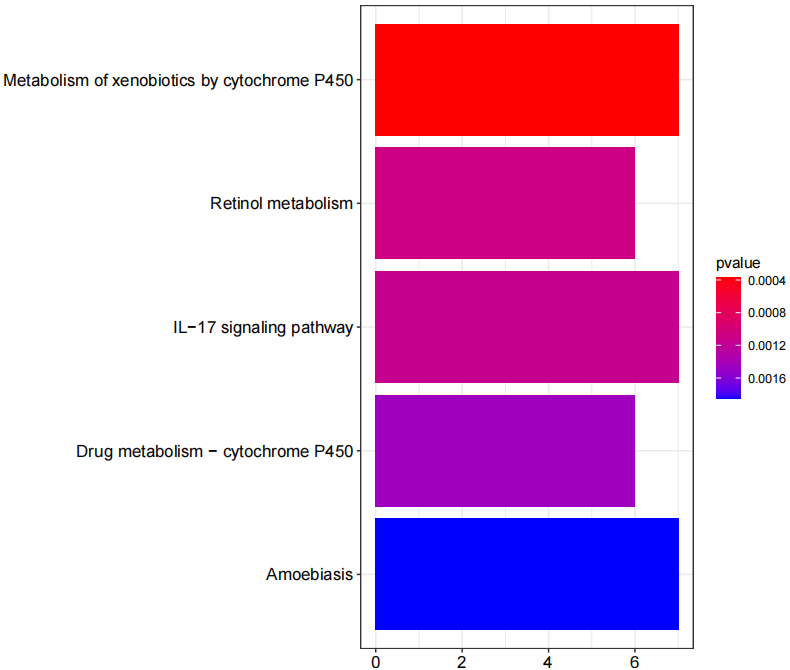

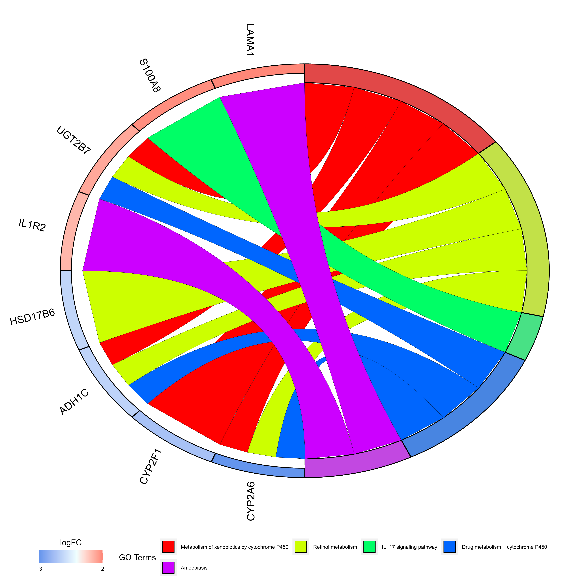


**Figure S10. Gene Ontology (GO) and Kyoto Encyclopedia of Genes and Genomes (KEGG) Enrichment Analysis of all Differentially Expressed lncRNAs (n = 580) between High-Risk and Low-Risk Subgroups.** (A) Bar chart of GO enrichment analysis. (B) Eight significantly enriched pathways were identified in the GO enrichment analysis of differentially expressed lncRNAs. (C) Bar chart of KEGG pathway analysis. (D) KEGG pathway of differential lncRNAs. BP, biological; CC, cellular components; MF, molecular functions.

A B


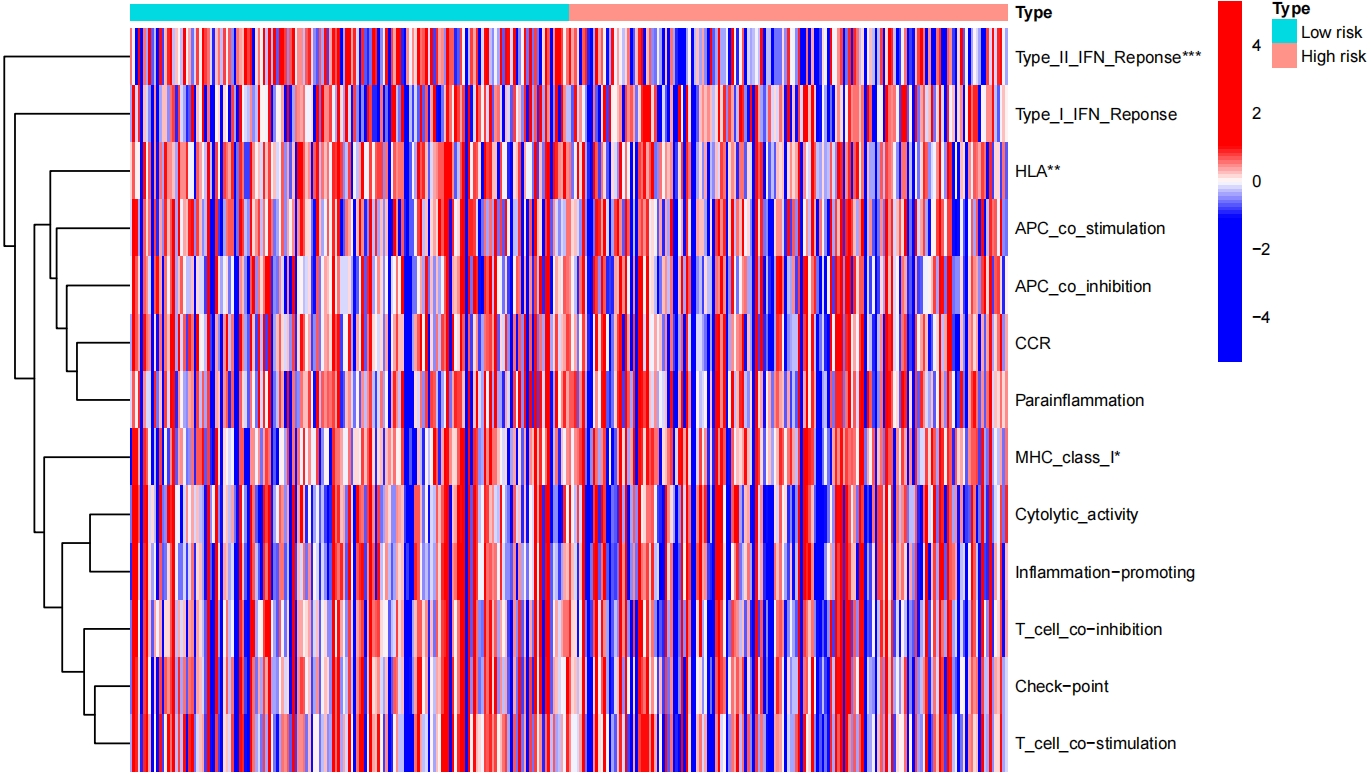
Train Test


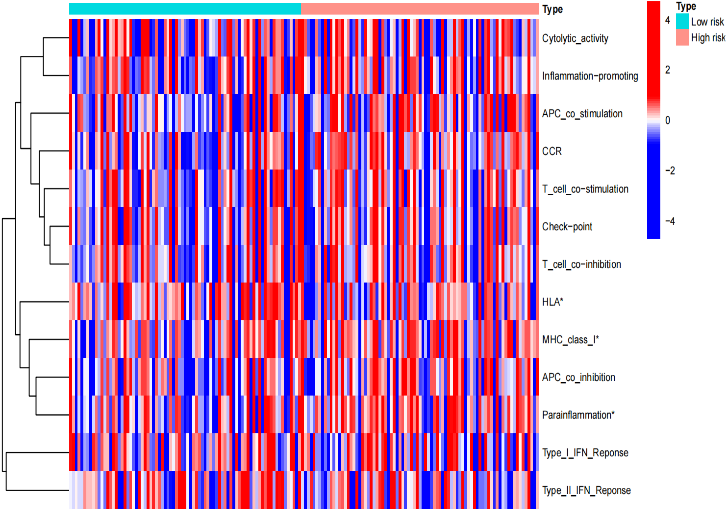


C


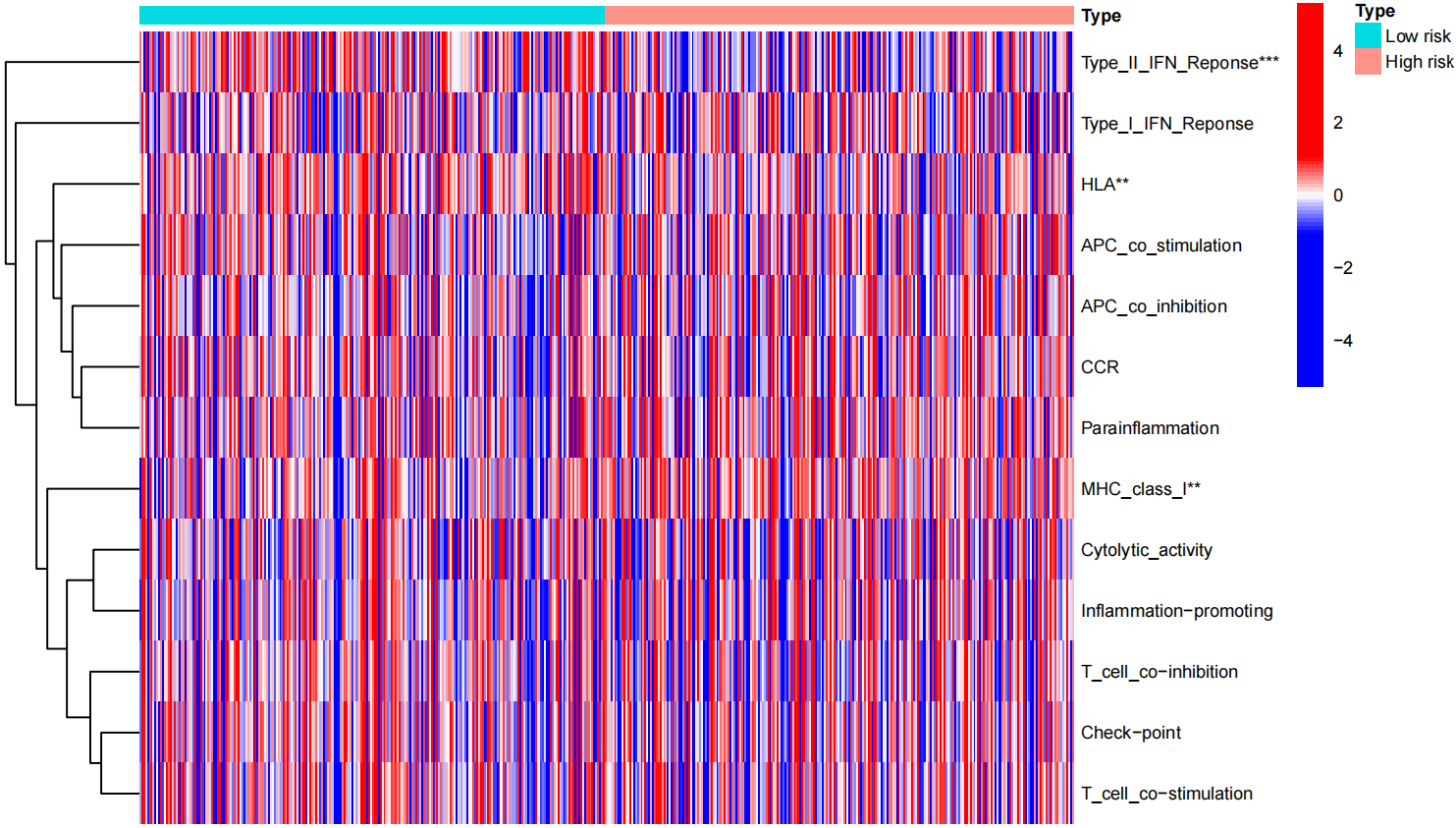
Entire

**Figure S11.** **Comparison of 13 Immune-Related Enrichment Pathways between High-Risk and Low-Risk Subgroups.** (A) Heatmap of immune functions in the training cohort. (B) Heatmap of immune functions in the testing cohort. (C) Heatmap of immune functions in the entire cohort. *P<0.05, **P<0.01, ***P<0.001.

A B C

Test Train Entire


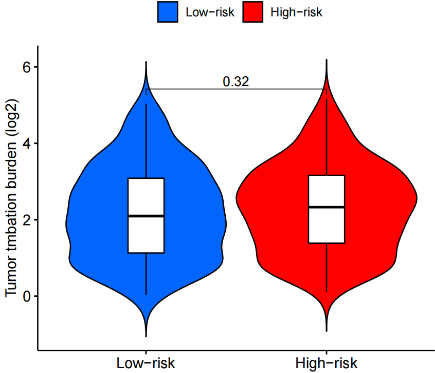

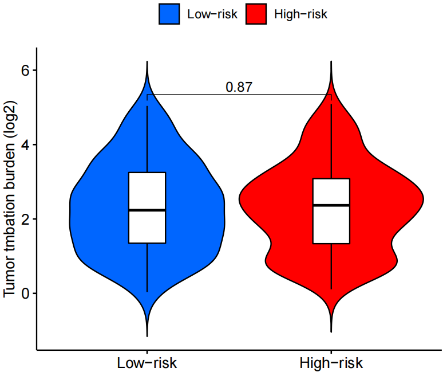

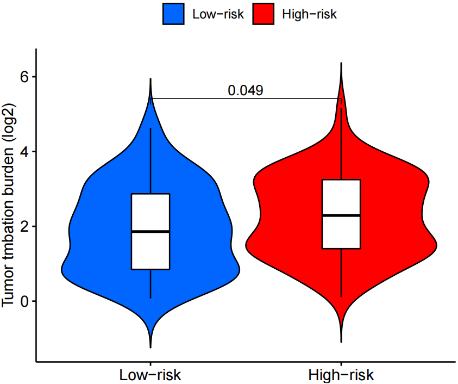


D E F


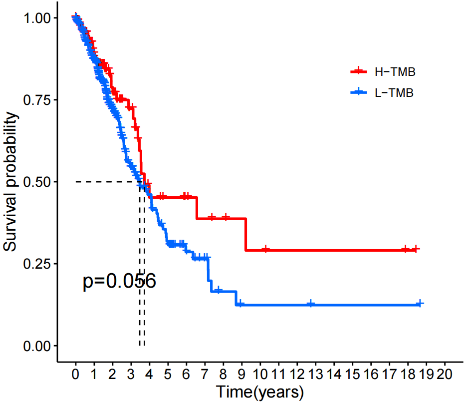

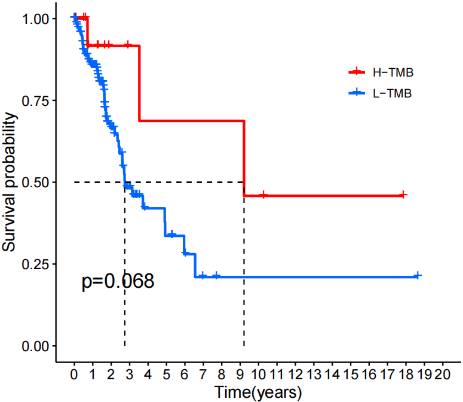

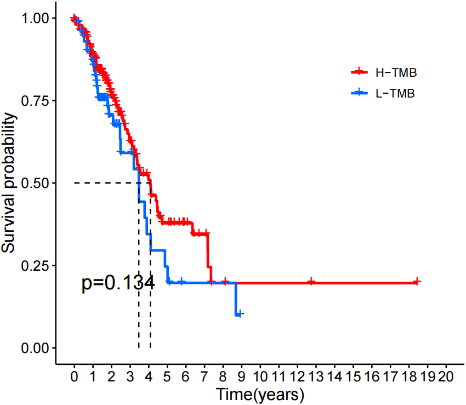
 Train Test Entire

G H I


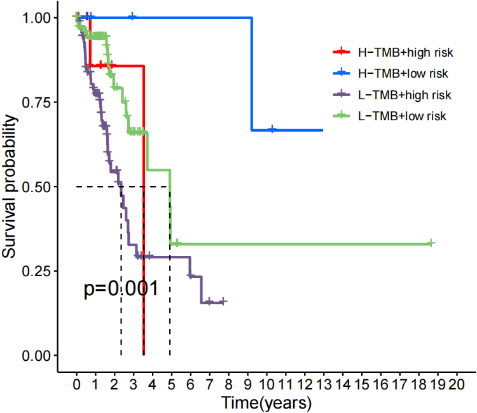

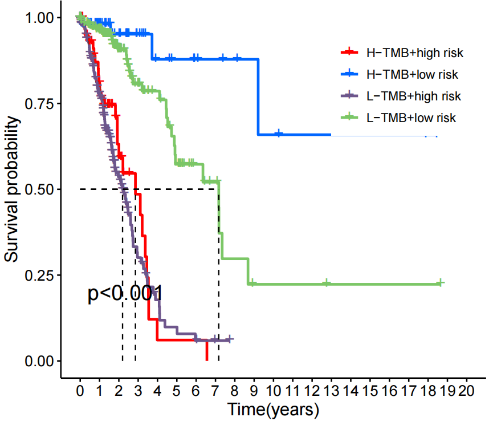

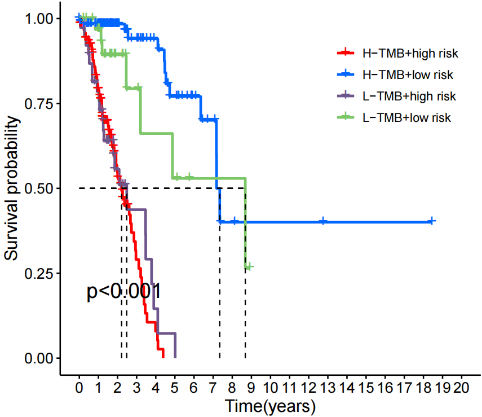
 Train Test Entire

**Figure S12. Tumor Mutational Burden and Survival Analysis of Lung Adenocarcinoma (LUAD).** Tumor mutation burden (TMB) differences between high-risk and low-risk in the (A) testing cohort, (B) training cohort, and (C) entire cohort. Survival analysis of the high TMB and low TMB subgroups in the (D) training cohort, (E) testing cohort, and (F) entire cohort. Combined survival analysis of the high and low TMB subgroups and the high and low-risk subgroups in the (G) training cohort, (H) testing cohort, and (I) entire cohort.

A B C

Train Test Entire


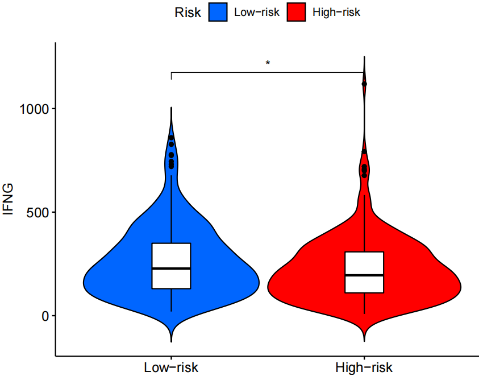

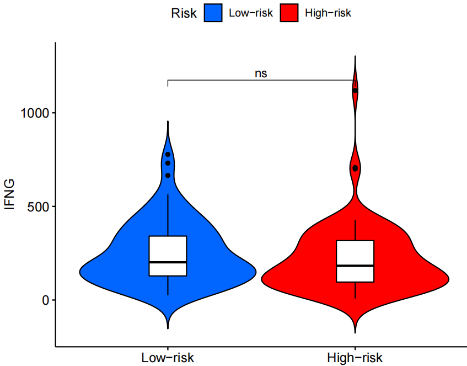

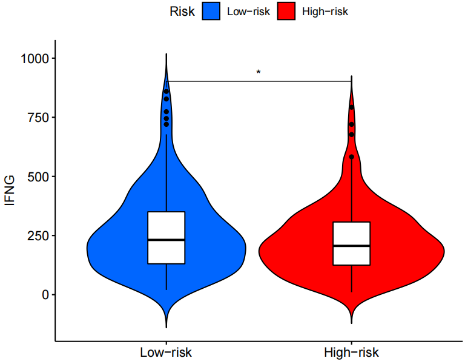


D E F

Train Test Entire


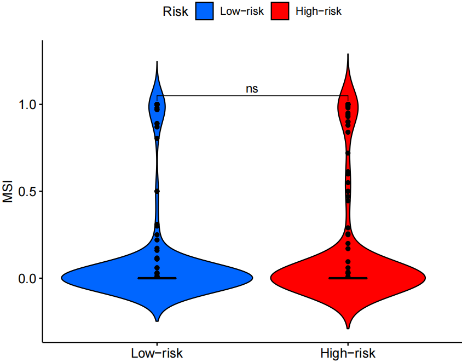

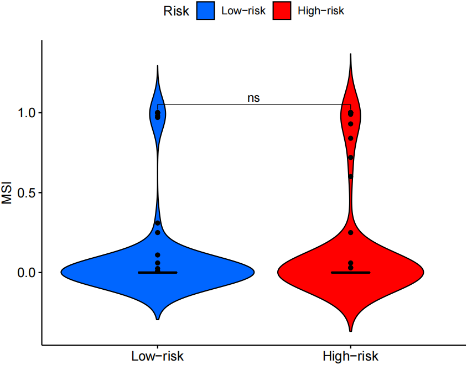

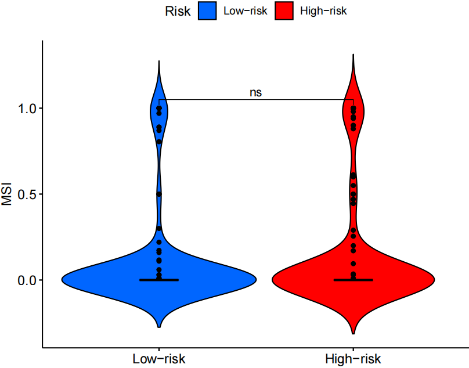


G H I

Train Test Entire


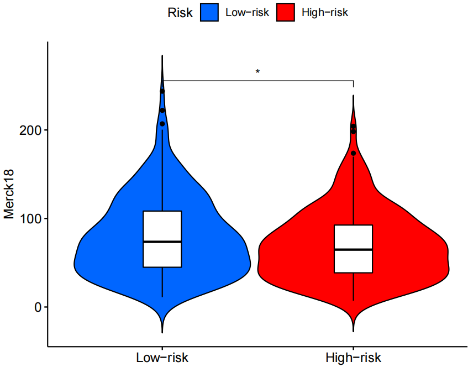

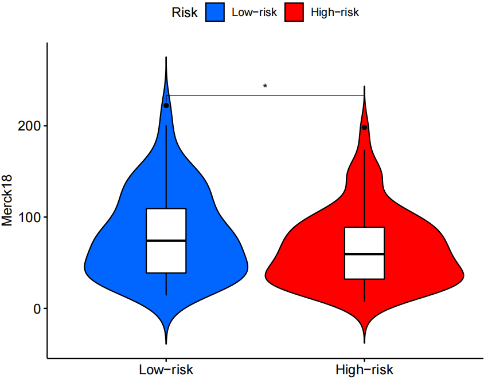

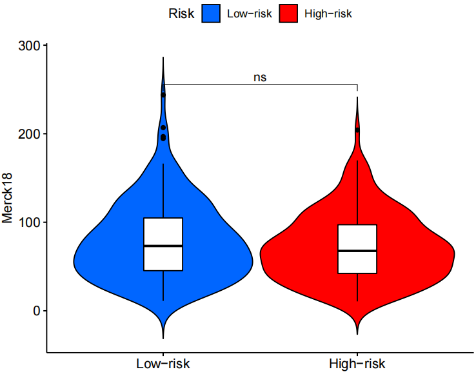


J K L

Train Test Entire


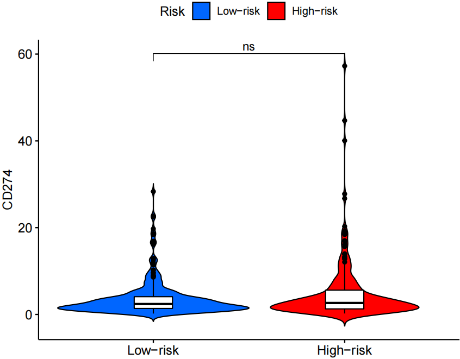

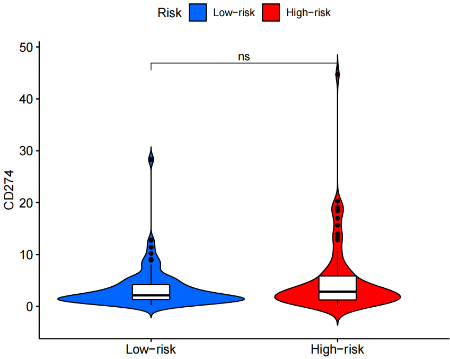

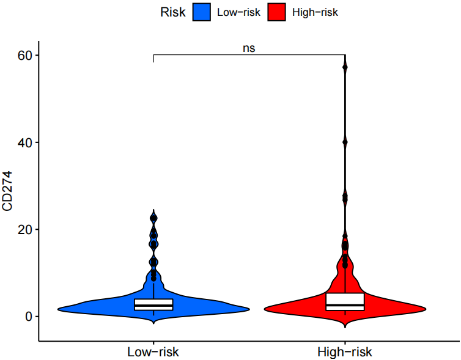


M N O

Train Test Entire


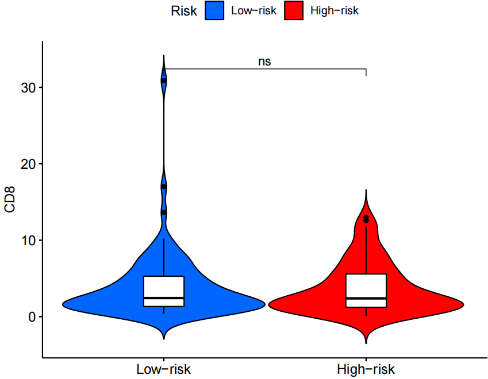

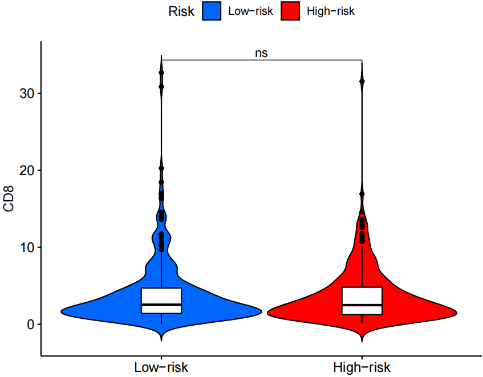

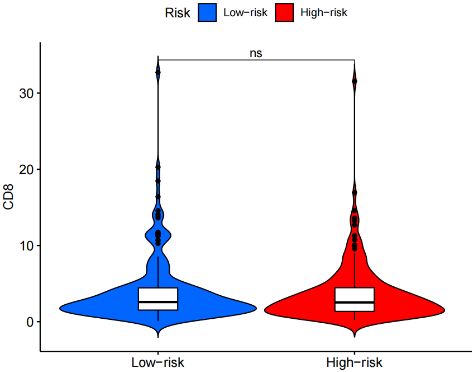


**Figure S13. Tumor Microenvironment Results Based on FLncSig.** Differential analysis of (A-C) IFNG, (D-F) MSI, (G-I) Merck18, (J-L) CD274, and (M-O) CD8 in different risk subgroups in the training cohort, testing cohort, and entire cohort. *P<0.05, **P<0.01, ***P<0.001 and ns, no statistical significance.

A B


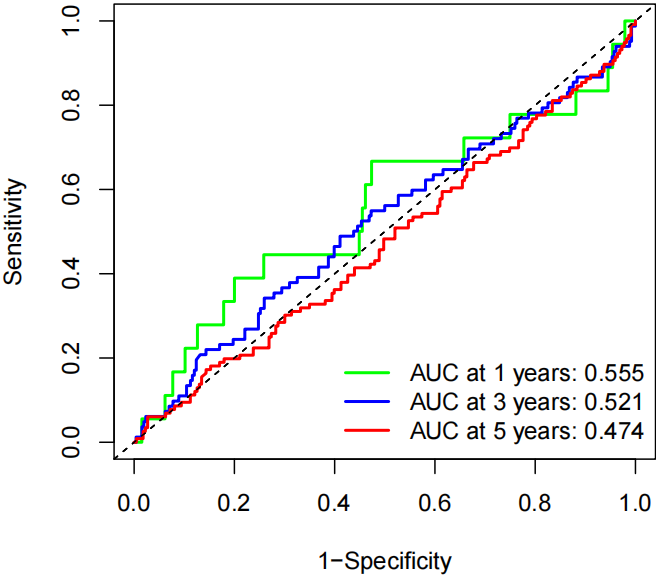

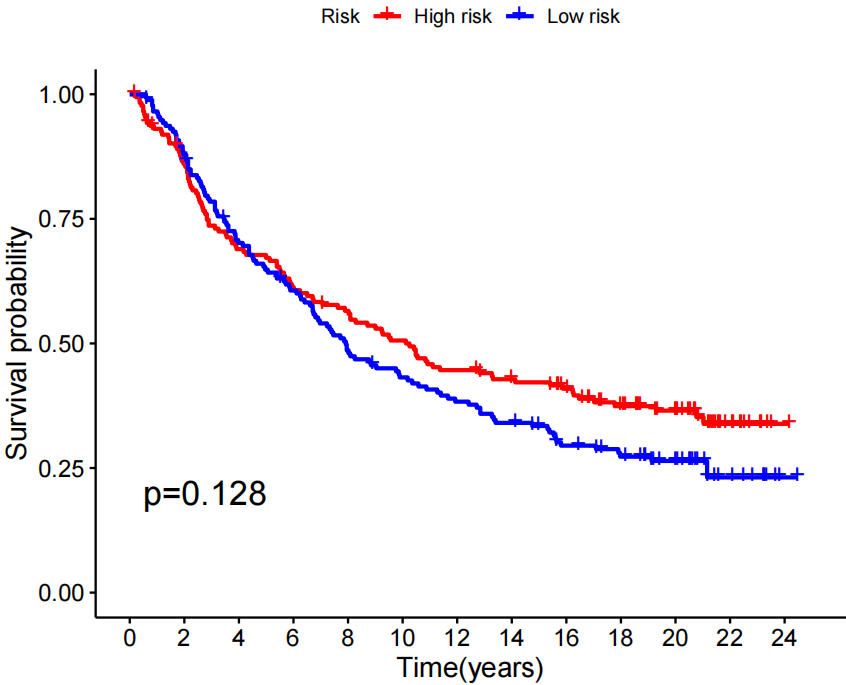


C


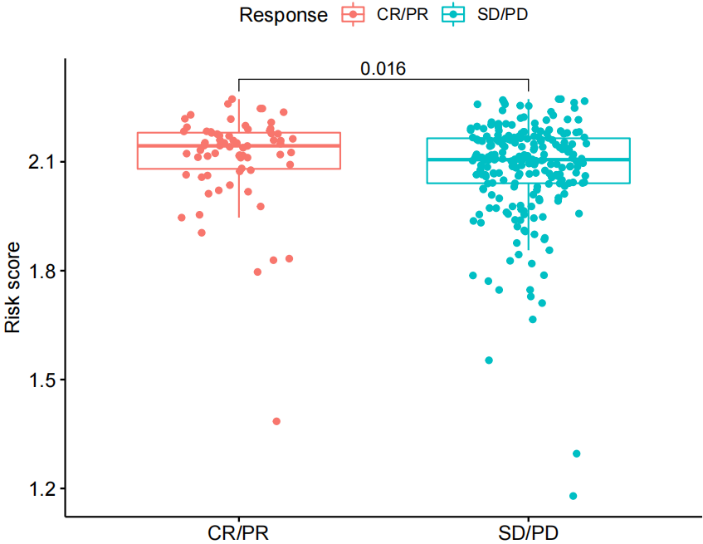


**Figure S14. Validation with IMvigor210 Immunotherapy Model.** (A) Survival probability analysis of target lncRNAs in lung adenocarcinoma (LUAD) patients between high and low-risk subgroups in the IMvigor210 cohort. (B) ROC curve analysis was used to evaluate the accuracy of target lncRNAs in prognostic 1, 3, and 5-year clinical survival probability of LUAD patients in IMvigor210 bladder cancer. (C) LUAD patients target lncRNAs risk score in response to different immunotherapy agents in IMvigor210 bladder cancer.

**Table S1. Results of univariate Cox regression analysis of the entire cohort.**

| **gene** | **HR** | **HR.95L** | **HR.95H** | **pvalue** |
| --- | --- | --- | --- | --- |
| AC027020.2 | 0.900074336492765 | 0.815984580382894 | 0.992829804250396 | 0.03539924170773 |
| AL365181.3 | 1.01055793327405 | 1.00520707291289 | 1.01593727702672 | 0.000105621069843816 |
| AF111169.3 | 0.707223513396973 | 0.55021889507678 | 0.90902930156872 | 0.00683777083603178 |
| LINC02285 | 0.769313367715307 | 0.600967471150768 | 0.984817125978832 | 0.0373985826149057 |
| AL591848.2 | 0.538803214546601 | 0.304178637842762 | 0.954402669643814 | 0.034010098233624 |
| GORAB-AS1 | 1.11167726178447 | 1.01267512751335 | 1.22035813934053 | 0.0261060212655977 |
| AC004080.2 | 1.08293433552249 | 1.03215192267248 | 1.13621526956714 | 0.00114845980215798 |
| tumorAS1 | 1.30659935571208 | 1.04874457142254 | 1.62785288512297 | 0.0171119351791932 |
| FAM78B-AS1 | 1.54969567137818 | 1.12531026125365 | 2.13412847689919 | 0.00729516295862941 |
| AL606489.1 | 1.08410455027604 | 1.04537214055317 | 1.12427204661042 | 1.35856587324959e-05 |
| AC011611.2 | 1.38694334304682 | 1.14976442814587 | 1.67304866086694 | 0.000629853428038503 |
| AC007728.2 | 0.786468105672075 | 0.620938673943865 | 0.99612426668618 | 0.0463523455855686 |
| AL590226.1 | 0.820658019529936 | 0.719994365086321 | 0.935395633183952 | 0.00307412012424966 |
| LINC00847 | 0.986511772592371 | 0.973339408716591 | 0.999862400256222 | 0.0477003794824091 |
| DEPDC1-AS1 | 4.82770492953787 | 2.38001041588258 | 9.7927028937146 | 1.28367388542163e-05 |
| AC008073.2 | 0.407311154227321 | 0.169054945361649 | 0.981351808449541 | 0.0452932505811988 |
| LINC00628 | 1.88283648626068 | 1.31509662889205 | 2.69567509802009 | 0.000548410211581988 |
| AC064875.1 | 1.58787292819046 | 1.00553295008231 | 2.50746674773188 | 0.0472966092707372 |
| AC090236.2 | 0.662104916983504 | 0.449126354482483 | 0.976079263036947 | 0.0373215183306912 |
| AC099788.1 | 1.34695439013482 | 1.0273291443911 | 1.76602225198117 | 0.0311580198111756 |
| AC026355.3 | 1.10545568848133 | 1.02595128116639 | 1.19112115909287 | 0.0084697310997158 |
| LINC01082 | 0.641045329841093 | 0.4458374773691 | 0.921724026737366 | 0.0164002098213968 |
| DLGAP1-AS2 | 1.02106058109818 | 1.0057404890669 | 1.03661403871669 | 0.00689096224643526 |
| KMT2E-AS1 | 0.979124337603289 | 0.959820042558253 | 0.998816888561582 | 0.0378492762150111 |
| AF131215.6 | 0.88736850584262 | 0.80009056694602 | 0.984167165183551 | 0.0236922445624163 |
| HIF1A-AS1 | 1.20519331499773 | 1.05788691949692 | 1.37301151923304 | 0.00501617142436243 |
| LINC01843 | 1.03582970631423 | 1.01909037841524 | 1.05284399029607 | 2.28664423559726e-05 |
| LUADT1 | 1.19686116286839 | 1.0009929558729 | 1.43105566805264 | 0.0487411939910493 |
| AP001189.1 | 0.828775843617428 | 0.692280538374225 | 0.992183603163029 | 0.0408126416965732 |
| AL135960.1 | 0.23330392335349 | 0.0677669298319502 | 0.803204760597975 | 0.0210328208192996 |
| LINC02323 | 1.12737085830617 | 1.07390291791424 | 1.18350088351234 | 1.32451295261076e-06 |
| AL021026.1 | 0.573753614252676 | 0.335903376638552 | 0.980023520937201 | 0.0419680965149103 |
| LINC02709 | 1.21505524068079 | 1.0520724806062 | 1.40328662247222 | 0.00803140793580747 |
| AL772337.2 | 1.20126855348331 | 1.05867632744877 | 1.36306640676984 | 0.0044495475954983 |
| LINC00996 | 0.840768240681752 | 0.736831904518121 | 0.959365671063588 | 0.00999170853315355 |
| LINC01352 | 0.697922329274609 | 0.494555746305597 | 0.984915414164673 | 0.0407119535849591 |
| SH3BP5-AS1 | 0.951126207885257 | 0.912470270562301 | 0.991419767318788 | 0.0179316809256616 |
| AL512363.1 | 1.13686170678945 | 1.03548731231273 | 1.24816067275374 | 0.00710787499432714 |
| AL035458.2 | 1.08546383257029 | 1.00739713816424 | 1.16958018559122 | 0.0312796933136497 |
| AC012085.2 | 1.03714244100137 | 1.01290398630833 | 1.06196091383419 | 0.00250595287593591 |
| AP000302.1 | 0.380789585419503 | 0.159933577422275 | 0.906630806995012 | 0.0291519333647097 |
| AC004943.2 | 1.09655951085447 | 1.00666262619729 | 1.19448435806908 | 0.0346759336772672 |
| AC018529.1 | 0.638859360191904 | 0.451407664725562 | 0.904152308430436 | 0.0114531597433896 |
| AL031667.3 | 1.055275843884 | 1.01613260468903 | 1.09592695042582 | 0.00527381951020459 |
| AP002026.1 | 0.787341291485007 | 0.650801143332134 | 0.952527996652446 | 0.0138756877263351 |
| AC006017.1 | 0.85143395212193 | 0.740034021083052 | 0.979603307649301 | 0.0245761730364081 |
| AC015914.1 | 0.793748457522411 | 0.633643570597562 | 0.994307593502521 | 0.0444703507140493 |
| FENDRR | 0.893230476233486 | 0.820046901561947 | 0.972945184174967 | 0.00963040556747013 |
| AL122010.1 | 0.914788403278734 | 0.862619365921295 | 0.970112492060153 | 0.00295118091730569 |
| AC087501.4 | 0.796909028679255 | 0.656399380622775 | 0.96749634252851 | 0.0217990893535553 |
| LINC00592 | 1.08793788795567 | 1.03864615592478 | 1.1395688910008 | 0.000366888453545186 |
| MEG3 | 1.00584977246163 | 1.00272218960587 | 1.00898711053634 | 0.000241733394545725 |
| AC087854.1 | 0.691122933358591 | 0.485657118658156 | 0.983514686933666 | 0.0401398578668072 |
| AC025279.1 | 0.198285618913248 | 0.043588408668354 | 0.902010141433653 | 0.0363143093973259 |
| AL353801.3 | 0.731647995979619 | 0.559237864659522 | 0.957211276004895 | 0.0226717921744864 |
| ABALON | 1.1183527167626 | 1.03246249362577 | 1.2113881199675 | 0.00607834554196909 |
| AC068580.2 | 1.11951413190135 | 1.02763069717317 | 1.21961313045092 | 0.00977321474122288 |
| LINC01337 | 0.42210121351391 | 0.232985051441869 | 0.764724746704915 | 0.00444611138278454 |
| LINC01096 | 1.22032290411823 | 1.03994238278238 | 1.43199086311994 | 0.0146921565211559 |
| LINC02617 | 1.01041538203749 | 1.00083321503135 | 1.02008929052779 | 0.033066241146591 |
| LINC00471 | 1.15907160254323 | 1.0011872572422 | 1.34185385411587 | 0.0481722578159708 |
| AP001178.1 | 1.26370506942584 | 1.0048611713264 | 1.58922500745512 | 0.0453457475456383 |
| AC004830.2 | 1.31031147081809 | 1.09999969662129 | 1.56083329461914 | 0.00246430708919087 |
| CASC15 | 1.03259358959148 | 1.00727235827093 | 1.0585513565524 | 0.0113418217506531 |
| AC023090.1 | 1.05118695842021 | 1.00272521476313 | 1.10199085979279 | 0.0381743841830789 |
| PAN3-AS1 | 0.847303210885745 | 0.722325744993625 | 0.993904393070788 | 0.0418450072804703 |
| KCNQ1-AS1 | 1.38660822042745 | 1.14307794375588 | 1.682022094346 | 0.000909849636792187 |
| AC245041.2 | 1.01438822359551 | 1.00033155529753 | 1.02864241632686 | 0.0448002088574715 |
| RASSF10-DT | 0.912176144155566 | 0.840707532316545 | 0.989720308171601 | 0.0272314435086328 |
| AC026462.3 | 1.09349933384086 | 1.02790356519193 | 1.16328110301585 | 0.00462687883438794 |
| AL596223.1 | 1.08678906519718 | 1.02100342862696 | 1.15681342404551 | 0.00899071568753341 |
| LINC01956 | 1.05897356463245 | 1.00722386848597 | 1.11338208483488 | 0.0249910544569037 |
| FAM30A | 0.916719953434662 | 0.844818444101499 | 0.994740916101832 | 0.0369341120863739 |
| AL109811.1 | 0.512169912880728 | 0.28061646997206 | 0.93479195888385 | 0.0292852740901982 |
| AC124852.1 | 1.17410237413834 | 1.0630731882868 | 1.29672763845999 | 0.00154168538355393 |
| AC010976.2 | 0.810317325155148 | 0.676175095417948 | 0.97107120906418 | 0.0227333373591394 |
| AC010999.2 | 0.562749926316356 | 0.367079407321453 | 0.862721997618734 | 0.0083559050747207 |
| MAGEA4-AS1 | 1.04345043815379 | 1.00459005148472 | 1.08381405457298 | 0.0280591791952323 |
| LINC00968 | 0.790218359446041 | 0.635847641096913 | 0.982067110492619 | 0.0337419577904144 |
| AC021086.1 | 2.48153152257411 | 1.19279348533862 | 5.16266962657058 | 0.0150307316323068 |
| TMPO-AS1 | 1.09922075349912 | 1.05012710580702 | 1.15060953882779 | 4.94748862086894e-05 |
| LINC02178 | 1.04031106960529 | 1.02220197980475 | 1.05874097578056 | 1.02969594645382e-05 |
| LINC02587 | 1.01053917000649 | 1.0041614496001 | 1.01695739716365 | 0.00117220194320479 |
| AL591686.1 | 0.342646191710652 | 0.137882288179281 | 0.85149742tumor | 0.0211053810668783 |
| AP000695.2 | 1.11402191866362 | 1.05722315384764 | 1.1738721676179 | 5.25295303303744e-05 |
| PRR34 | 1.46716910335071 | 1.02751030410911 | 2.09495240020324 | 0.0349191877519117 |
| AC090559.1 | 0.941808336659223 | 0.898195661058074 | 0.98753866385407 | 0.0132002984013525 |
| AL391807.1 | 0.504235383756956 | 0.280537050153929 | 0.906309245402764 | 0.0220907752239189 |
| ATP13A4-AS1 | 0.946756478920246 | 0.903615245797879 | 0.991957400614682 | 0.0214868189712314 |
| IL12A-AS1 | 1.09992425267418 | 1.03874383119441 | 1.16470810732008 | 0.00110711417061283 |
| AC084117.1 | 1.04206700694672 | 1.00352085803816 | 1.08209374849446 | 0.0321350056504007 |
| AC007128.1 | 1.06375413606779 | 1.00830447065735 | 1.12225314369936 | 0.023651252829863 |
| AC005332.4 | 0.916566049699898 | 0.852017217918799 | 0.986005101533689 | 0.0193757027748284 |
| AL355102.4 | 1.23726696951845 | 1.00244254677219 | 1.52709954180473 | 0.0474029018681014 |
| AC148476.1 | 1.02845249819413 | 1.0032351246644 | 1.05430373701836 | 0.0267624495215382 |
| AF131215.5 | 0.911574537435258 | 0.840305868478876 | 0.988887699671223 | 0.025813824351394 |
| AC009318.2 | 1.08268872663129 | 1.01512577662956 | 1.15474841222778 | 0.0156657759692463 |
| AL136295.6 | 0.915516833656535 | 0.845152527685305 | 0.991739414190788 | 0.0305214011852772 |
| LINC00543 | 1.07637260019865 | 1.02887213308497 | 1.12606604572381 | 0.0013934508611322 |
| AC005180.2 | 0.781319591220235 | 0.615757312418729 | 0.99139757062183 | 0.0422475455588526 |
| GACAT2 | 1.13724588067896 | 1.00719102287312 | 1.2840942420554 | 0.0379306613804044 |
| AC068228.2 | 1.04197472862098 | 1.0135406544414 | 1.07120649805917 | 0.00358280294464909 |
| ADPGK-AS1 | 0.297316147978973 | 0.109276019369486 | 0.808932210004515 | 0.0175407052591581 |
| AC107214.1 | 1.1825478119157 | 1.00062580886364 | 1.39754473158625 | 0.0491481317929801 |
| LINC02320 | 1.08032282833873 | 1.04502091699496 | 1.11681727556792 | 5.1671428065165e-06 |
| AC007773.1 | 1.04932358305303 | 1.00678747055879 | 1.0936568184943 | 0.0225865198143406 |
| AC092171.2 | 1.01795194839597 | 1.00270788335448 | 1.03342776739376 | 0.0208197637673615 |
| AC107021.2 | 1.06075253427258 | 1.00572218892164 | 1.11879398839969 | 0.0300151952366292 |
| LINC01876 | 1.05522277273449 | 1.00746187592232 | 1.10524787757163 | 0.0229331939266423 |
| AC009275.1 | 1.0587930227384 | 1.02048335798107 | 1.09854085932122 | 0.0023789843440602 |
| AC009318.3 | 1.10649746484168 | 1.01651336409744 | 1.20444716512719 | 0.0193653359898308 |
| AL356215.1 | 1.32727858826114 | 1.00619723342555 | 1.75081822165121 | 0.0451037270238196 |
| AC099524.1 | 0.728356729895554 | 0.539526604933734 | 0.983275933258755 | 0.0384417118069004 |
| AC005291.1 | 1.03927721580813 | 1.01143564625241 | 1.06788517420746 | 0.00542455295714957 |
| AC026355.2 | 0.916802153676929 | 0.870389078189568 | 0.965690183905998 | 0.00104881570388471 |
| AL161431.1 | 1.00817435347025 | 1.00228741318498 | 1.01409587072961 | 0.00643737057427191 |
| AC018647.1 | 0.31201337563415 | 0.147920145215427 | 0.658141231762823 | 0.00222443293378668 |
| AC004817.3 | 1.07577043748 | 1.01465481206018 | 1.14056723567509 | 0.0143856836613496 |
| AC105020.6 | 0.64842586724775 | 0.425578146321801 | 0.987964511218273 | 0.0437663362326413 |
| AC135050.6 | 0.989769812771202 | 0.981611090185081 | 0.997996347095498 | 0.0148964985257594 |
| AC010275.1 | 1.06195123586839 | 1.01123214920099 | 1.11521417535378 | 0.0160710059363004 |
| L3MBTL2-AS1 | 0.770753713933964 | 0.610807399454099 | 0.972583645964231 | 0.0282194514228125 |
| AC104072.1 | 1.05858445736172 | 1.01459755483911 | 1.10447836979609 | 0.00855793888420535 |
| AC026310.2 | 0.295973674413109 | 0.0877428295599995 | 0.998376920198305 | 0.0496947045307871 |
| AC090948.1 | 0.865076446637423 | 0.762808528822942 | 0.981055179969722 | 0.0239500029444689 |
| LINC00621 | 1.41317173329202 | 1.04530682995787 | 1.91049583772073 | 0.0245769286557678 |
| FGD5-AS1 | 1.00664830006416 | 1.00082003720961 | 1.01251050373389 | 0.0253094413533993 |
| LINC01235 | 1.1236590320175 | 1.03612424573434 | 1.2185890113397 | 0.00483909783787721 |
| AC104938.1 | 0.621569274135425 | 0.397545715340022 | 0.97183379833133 | 0.0370461874015064 |
| KTN1-AS1 | 1.15322983742462 | 1.05325905349436 | 1.26268941483495 | 0.00205936884974052 |
| SLC2A1-AS1 | 1.22447114497474 | 1.05830061880977 | 1.41673316468623 | 0.00649940299752528 |
| AL117335.1 | 1.30272133266941 | 1.06394390446197 | 1.59508679308629 | 0.0104682784386432 |
| AP005137.2 | 1.12623973083672 | 1.0664401105147 | 1.18939255829658 | 1.94748423859919e-05 |
| AC105036.3 | 1.26983558572256 | 1.02777697278727 | 1.56890303778106 | 0.0268373725779523 |
| AC079949.1 | 1.01020379244945 | 1.00028610558619 | 1.02021981169199 | 0.0437171175521479 |
| MIR193BHG | 1.09845787816848 | 1.0539996783502 | 1.14479134566632 | 8.39311386060915e-06 |
| AL606834.1 | 1.05729574211145 | 1.00412816493337 | 1.11327848906737 | 0.0343056706375651 |
| AL009178.2 | 1.21384488494803 | 1.0184365076376 | 1.44674645268981 | 0.0304676067839329 |
| AC108134.4 | 0.931326444483299 | 0.867690201801646 | 0.999629757709518 | 0.0488132833284748 |
| HAGLR | 0.99466390742308 | 0.989501019281587 | 0.999853733802576 | 0.0438989247752425 |
| AL359878.1 | 0.712798689479804 | 0.521658622236169 | 0.973974070525578 | 0.0335429240623782 |
| AC123595.1 | 0.795476777225003 | 0.686959109431153 | 0.921136781530217 | 0.00223026867425132 |
| DDX11-AS1 | 1.21475913736198 | 1.0575042460051 | 1.39539842736225 | 0.00595181346225701 |
| AL024497.1 | 1.1231261827222 | 1.04660175296641 | 1.20524585281927 | 0.00125954475829745 |
| AP000438.1 | 0.41174723240622 | 0.202345309343191 | 0.837853785415097 | 0.0143638663243906 |
| AP002840.2 | 0.936446636763186 | 0.882050899033784 | 0.994196938595824 | 0.0315094583599669 |
| FAM66C | 1.51129530104336 | 1.14021476275544 | 2.00314323368011 | 0.00406906884000374 |
| AC009065.4 | 0.903233394126211 | 0.822637115751332 | 0.991725936799776 | 0.0328270977008781 |
| LINC01800 | 0.159773075578381 | 0.0440837455961236 | 0.579066849574136 | 0.0052456421320353 |
| AL359232.1 | 1.66091549012998 | 1.05036424248516 | 2.62636536333983 | 0.0299966733960379 |
| AL021368.2 | 0.761648876314389 | 0.580252636957899 | 0.999752476494238 | 0.0497918708581807 |
| AC024896.1 | 1.02089942290548 | 1.00968522626086 | 1.03223817144322 | 0.000242264996988585 |
| FOCAD-AS1 | 0.666813592335906 | 0.4508804056353 | 0.986160324038495 | 0.0423801736952071 |
| GSEC | 1.04787655113082 | 1.02258317979085 | 1.07379554847988 | 0.000175901976765164 |
| LINC00598 | 1.28297388721317 | 1.114742560187 | 1.47659383794833 | 0.000511546867757586 |
| LINC00941 | 1.03722555867994 | 1.02620357662576 | 1.04836592278928 | 2.00864322347581e-11 |
| LINC02198 | 0.891933650027584 | 0.806295799373647 | 0.986667221470745 | 0.0263789692078769 |
| AP003064.2 | 0.795293090793936 | 0.642010146105167 | 0.985173060116972 | 0.036019406282093 |
| AC046134.2 | 1.12641060401411 | 1.03332112377312 | 1.22788629753592 | 0.00683561062640955 |
| LINC02360 | 1.04689973946296 | 1.01007435648022 | 1.08506770561607 | 0.012120729479696 |
| AC027601.2 | 0.689052298724774 | 0.499322836433653 | 0.950873935125903 | 0.023419554391062 |
| AC024075.2 | 0.956851686690128 | 0.925783616912861 | 0.988962359665433 | 0.00881857614663393 |
| AC012676.4 | 0.661909174596601 | 0.455714996457591 | 0.961398590831597 | 0.030259806415568 |
| AL360270.1 | 1.13615053548883 | 1.00735335178552 | 1.28141534150212 | 0.0375889159402316 |
| LINC00623 | 1.05141591007507 | 1.01787080614612 | 1.08606653151253 | 0.00244024788214618 |
| LINC01150 | 0.863347546600981 | 0.766981070997451 | 0.971821879844555 | 0.0149621207961794 |
| AC060234.2 | 0.506881559321559 | 0.273383868957181 | 0.939810077896353 | 0.03100320615484 |
| AC034102.8 | 0.752892540137946 | 0.602980774144764 | 0.940075042689968 | 0.0122297378613908 |
| LINC01638 | 1.15301538604926 | 1.04062141299617 | 1.27754864916586 | 0.00651075075930044 |
| AL121985.1 | 0.455818791148829 | 0.207952639273722 | 0.999125431107883 | 0.0497451238060453 |
| AC005034.3 | 1.0375772356562 | 1.0154129052821 | 1.06022536679586 | 0.000813109913936192 |
| AP001528.1 | 1.09776623749284 | 1.01828489338855 | 1.18345142896994 | 0.0149953694665927 |
| AL356608.1 | 0.255017942726007 | 0.0708546631449377 | 0.917852802139125 | 0.0365150159895266 |
| LINP1 | 1.02181194699149 | 1.00944683293563 | 1.03432852622672 | 0.000513517179055657 |
| AC114550.2 | 0.537777959394233 | 0.290685719264027 | 0.994906575880127 | 0.0481284510585364 |
| GCC2-AS1 | 1.29124022201864 | 1.0722217373833 | 1.55499674444925 | 0.00703288804270919 |
| LINC00892 | 0.751468810058742 | 0.607346067506306 | 0.929791765689235 | 0.00853808100410746 |
| ABCA9-AS1 | 1.56770908551276 | 1.2839626282453 | 1.91416145823344 | 1.01685257333296e-05 |
| AC107959.1 | 0.765476361318656 | 0.594108125489503 | 0.986275114912568 | 0.0387491393170002 |
| LINC00602 | 1.7488169105869 | 1.23688850604408 | 2.47262430834305 | 0.00156111850624511 |
| AC004704.1 | 1.02094051065511 | 1.0092009976238 | 1.03281658336734 | 0.00044455514285128 |
| LINC02410 | 0.509924384104286 | 0.260126444325921 | 0.999601859695372 | 0.0498646123800452 |
| AL359962.1 | 1.05861123155399 | 1.01309185140899 | 1.10617584971557 | 0.0110853315341898 |
| AC008764.2 | 0.964300389704284 | 0.932004168590949 | 0.99771575377143 | 0.0364798160672983 |
| AC138625.1 | 1.22329464683091 | 1.00437905790292 | 1.48992532370163 | 0.0451346866206586 |
| AC012645.4 | 0.85319110766051 | 0.729516120950565 | 0.997832734994895 | 0.0469093253485732 |
| AC010998.1 | 0.762926945343493 | 0.584942734497733 | 0.995067533287585 | 0.0458854014524171 |
| AL031600.2 | 0.450346933280297 | 0.253046478628167 | 0.80148264229745 | 0.00668027403474454 |
| AL365181.2 | 1.02303558806355 | 1.01079157651885 | 1.03542791487144 | 0.00020955148888538 |
| HOXA11-AS | 1.11400432844467 | 1.0282410345631 | 1.20692094759743 | 0.00825841822010014 |
| AC009226.1 | 1.23021162045601 | 1.0207503688945 | 1.48265499305583 | 0.0295829008026549 |
| AL162632.3 | 3.69590163476944 | 1.6984077896363 | 8.04264380865598 | 0.000983583101304433 |
| AC105020.5 | 0.686704909122262 | 0.530769393442651 | 0.888452947812196 | 0.00423727227693341 |
| AL138826.1 | 1.08191819493626 | 1.00344904187558 | 1.16652359181711 | 0.0404041934487167 |
| AC022167.3 | 0.563979573247144 | 0.359322551704775 | 0.885201770751547 | 0.0127696530968543 |
| AL590428.1 | 1.46118039939895 | 1.11935056220074 | 1.90739901482694 | 0.00528413104779609 |
| GLCCI1-DT | 0.661247844120438 | 0.447525872703041 | 0.977035604026555 | 0.0378384449190725 |
| AC087752.3 | 0.849131090799612 | 0.766026138768901 | 0.941251966311898 | 0.00185773903879497 |
| PLUT | 1.20735127767947 | 1.09636948943615 | 1.32956737829682 | 0.00012810323155763 |
| LINC02404 | 1.0124782954335 | 1.000747729195 | 1.024346365041 | 0.0370081924933443 |
| LINC02448 | 1.19262471152563 | 1.10987926629595 | 1.28153912388008 | 1.5740233517746e-06 |
| AP000346.1 | 0.628936280513909 | 0.404247266732128 | 0.978512107563084 | 0.0397549722035545 |
| C20orf197 | 0.962723675976729 | 0.929658034367036 | 0.996965380842633 | 0.0331383352410423 |
| CTBP1-DT | 1.03835960967607 | 1.0031507870153 | 1.07480419988964 | 0.0324600122961379 |
| AC103591.3 | 0.947287850739426 | 0.897790108123755 | 0.999514545815006 | 0.0479628330067552 |
| AL442125.1 | 1.03270627371899 | 1.00320083943396 | 1.06307950099037 | 0.0295520155066482 |
| ID2-AS1 | 1.56518911841024 | 1.07388385631271 | 2.28126809243743 | 0.0197629658150211 |
| AC010327.6 | 1.16948203967476 | 1.00126327036714 | 1.36596266096961 | 0.0481664230404989 |
| LINC00707 | 1.02107055676441 | 1.01276242160848 | 1.02944684720365 | 5.6657299358137e-07 |
| AP000977.1 | 0.291992556403562 | 0.0928063110336035 | 0.918683783953188 | 0.0352921619617768 |
| LINC02555 | 1.01322226015262 | 1.0028709247566 | 1.02368043895374 | 0.0121712237967999 |
| AL049555.1 | 1.02342752099544 | 1.01022858933136 | 1.03679890055786 | 0.000471329299788763 |
| AC046143.1 | 1.10112279983907 | 1.04201788060186 | 1.16358024453967 | 0.000621293504370194 |
| AC007671.1 | 0.643030112916971 | 0.425912424999818 | 0.97082804315509 | 0.0356568127785474 |
| AC026356.1 | 1.06657897560281 | 1.00387293410852 | 1.13320189492724 | 0.0370691343561036 |
| CYP1B1-AS1 | 0.750027118342993 | 0.563999286807342 | 0.99741381134415 | 0.0479547843452179 |
| LINC01138 | 1.09858153110161 | 1.03864520471221 | 1.16197655850341 | 0.00102122706828614 |
| AP001094.3 | 1.22573904264002 | 1.00545527314966 | 1.49428447070111 | 0.0440316086601743 |
| LINC02550 | 1.07654117132139 | 1.00703026713361 | 1.15085010984706 | 0.0303358683213502 |
| AC090617.5 | 0.907419292091338 | 0.839581500142123 | 0.980738345854642 | 0.0142630962490632 |
| DLGAP1-AS1 | 1.00880311751466 | 1.00031772558364 | 1.01736048845234 | 0.0419843662895513 |
| AC112721.2 | 1.04633410196824 | 1.0125930868637 | 1.08119941479419 | 0.00676368323481789 |
| FLG-AS1 | 1.7999454619879 | 1.43700964336122 | 2.25454552869444 | 3.12827117556518e-07 |
| TSPOAP1-AS1 | 0.720639204846623 | 0.547579790482264 | 0.948393042600416 | 0.0193813980635348 |
| AC007613.1 | 0.610590436190649 | 0.387570077396567 | 0.961943923204402 | 0.0333977797834225 |
| LINC02728 | 0.625632803204484 | 0.396747438710681 | 0.986563153923552 | 0.043572749804963 |
| AL024497.2 | 1.08879835123933 | 1.04280132205898 | 1.13682426804061 | 0.000111994684695231 |
| MIR223HG | 0.925074795545666 | 0.86612485612352 | 0.988036968692899 | 0.0204382465130329 |
| AL358115.1 | 1.29971961097911 | 1.05897843905071 | 1.59518929269041 | 0.012132719898511 |
| LINC01711 | 1.06880264938 | 1.01034623700583 | 1.13064122127781 | 0.0204145594616605 |
| LINC01338 | 1.1060561620723 | 1.01516211132125 | 1.20508854695718 | 0.0212277149086074 |
| MIR4435-2HG | 1.03543380836814 | 1.0056280827215 | 1.06612294339503 | 0.0194616599913443 |
| LINC00862 | 1.05139179725229 | 1.00002418576536 | 1.10539797643333 | 0.0498894849661575 |
| AC125807.2 | 1.10080617961915 | 1.03956067236217 | 1.16565995357849 | 0.00100775691075166 |
| LINC01269 | 1.02311849529664 | 1.000391301447 | 1.0463620124485 | 0.0461413591336993 |
| AC016168.1 | 1.26256450260186 | 1.01483458591296 | 1.57076743871143 | 0.0364293082069303 |
| ZEB2-AS1 | 0.445124159078132 | 0.206371131676582 | 0.960093184474687 | 0.0390363087603401 |
| STXBP5-AS1 | 1.63653675513164 | 1.15181896524885 | 2.32523741291076 | 0.00598370680514821 |
| TM4SF19-AS1 | 1.12532841387258 | 1.04966516498379 | 1.20644571365624 | 0.000884627304099929 |
| AC005291.2 | 1.02131874609956 | 1.008879149942 | 1.03391172391097 | 0.000741416189263892 |
| FRMD6-AS1 | 1.58476111759657 | 1.25094442188153 | 2.00765737942895 | 0.000136059711460992 |
| AC133785.1 | 1.15947705002919 | 1.06793581011704 | 1.25886501492731 | 0.00042129215310981 |
| AC005180.1 | 0.695936866206565 | 0.499239309311358 | 0.970132184529876 | 0.0324452079301877 |
| AL137779.1 | 0.649380946713751 | 0.441341572601285 | 0.955485818998097 | 0.0284477721015956 |
| LINC00857 | 1.05135177788922 | 1.02865943058329 | 1.07454472102982 | 6.8583180302061e-06 |
| AP000695.1 | 1.06489675647512 | 1.02745161698602 | 1.10370657187516 | 0.000575789524385656 |
| LINC02147 | 0.10088391577722 | 0.0216729005177021 | 0.469598633290098 | 0.00346364691838044 |
| AL353152.1 | 0.585598707233393 | 0.398040383804421 | 0.861535311155563 | 0.00659621882491494 |
| AC007686.2 | 0.242396846310041 | 0.0753280077765219 | 0.780005111450285 | 0.0174718141876433 |
| AC091057.1 | 1.12197453070584 | 1.02180395098391 | 1.23196514002559 | 0.0158647653346004 |
| AC026368.1 | 1.03939870870033 | 1.01326320187145 | 1.06620833920797 | 0.00293918582379964 |
| TMEM18-DT | 0.26141146121509 | 0.070913459335955 | 0.963652777547693 | 0.0438433070836247 |
| AC244250.1 | 1.39892522447395 | 1.12198530550248 | 1.74422229424213 | 0.00285840279606844 |
| TARID | 1.2051151554344 | 1.11621910337044 | 1.30109091796802 | 1.82264442950164e-06 |
| AL590666.2 | 1.01451594705172 | 1.00593687158793 | 1.02316818867325 | 0.000880689068545508 |
| AL049836.1 | 1.03208916253678 | 1.01716048879875 | 1.04723694161957 | 2.14923483691842e-05 |
| LINC01312 | 1.89950102660853 | 1.57838481384697 | 2.28594707604472 | 1.1187704848376e-11 |
| AC092384.1 | 0.20309260504225 | 0.0420938876951781 | 0.979871627005176 | 0.0471124788792383 |
| ZFHX4-AS1 | 1.21536609376706 | 1.05234444467722 | 1.40364188678896 | 0.00794786102749245 |
| MHENCR | 0.983674981747719 | 0.970692069056644 | 0.996831539642373 | 0.0151776677605342 |
| AL513218.1 | 0.924332568725719 | 0.859676021850957 | 0.993851958052184 | 0.0334495452238149 |
| AL157931.1 | 1.04727286985455 | 1.00110785419676 | 1.09556673572738 | 0.0446324984197609 |

**Table S2.** **Clinical and statistical analysis of lung adenocarcinoma (LUAD) patients in training and testing cohorts.**

| Covariates | Type | Total | Test | Train | Pvalue |
| --- | --- | --- | --- | --- | --- |
| fustat | Alive | 190(64.63%) | 66(66.67%) | 124(63.59%) | 0.6947 |
| fustat | Dead | 104(35.37%) | 33(33.33%) | 71(36.41%) |  |
| age | <=65 | 147(50%) | 51(51.52%) | 96(49.23%) | 0.8051 |
| age | >65 | 147(50%) | 48(48.48%) | 99(50.77%) |  |
| gender | FEMALE | 155(52.72%) | 55(55.56%) | 100(51.28%) | 0.5687 |
| gender | MALE | 139(47.28%) | 44(44.44%) | 95(48.72%) |  |
| race | AMERICAN INDIAN OR ALASKA NATIVE | 1(0.34%) | 1(1.01%) | 0(0%) | 0.084 |
| race | ASIAN | 5(1.7%) | 2(2.02%) | 3(1.54%) |  |
| race | BLACK OR AFRICAN AMERICAN | 27(9.18%) | 14(14.14%) | 13(6.67%) |  |
| race | WHITE | 261(88.78%) | 82(82.83%) | 179(91.79%) |  |
| stage | Stage I | 153(52.04%) | 48(48.48%) | 105(53.85%) | 0.4079 |
| stage | Stage II | 72(24.49%) | 22(22.22%) | 50(25.64%) |  |
| stage | Stage III | 51(17.35%) | 21(21.21%) | 30(15.38%) |  |
| stage | Stage IV | 18(6.12%) | 8(8.08%) | 10(5.13%) |  |
| T | T1 | 96(32.65%) | 31(31.31%) | 65(33.33%) | 0.4014 |
| T | T2 | 162(55.1%) | 52(52.53%) | 110(56.41%) |  |
| T | T3 | 23(7.82%) | 9(9.09%) | 14(7.18%) |  |
| T | T4 | 13(4.42%) | 7(7.07%) | 6(3.08%) |  |
| M | M0 | 276(93.88%) | 91(91.92%) | 185(94.87%) | 0.4589 |
| M | M1 | 18(6.12%) | 8(8.08%) | 10(5.13%) |  |
| N | N0 | 189(64.29%) | 63(63.64%) | 126(64.62%) | 0.9587 |
| N | N1 | 60(20.41%) | 20(20.2%) | 40(20.51%) |  |
| N | N2 | 45(15.31%) | 16(16.16%) | 29(14.87%) |  |

**Table S3.** **Results of univariate Cox regression analysis of the training cohort.**

| Id | HR | HR.95L | HR.95H | pvalue |
| --- | --- | --- | --- | --- |
| AC027020.2 | 0.774477973394788 | 0.606112370245951 | 0.989612092936335 | 0.0410071055682236 |
| AF111169.3 | 0.617596951375662 | 0.416620156589778 | 0.915524581121215 | 0.0164226777393507 |
| AL591848.2 | 0.36065539986686 | 0.168500070409868 | 0.771942214247925 | 0.00862337106574737 |
| GORAB-AS1 | 1.59056982698718 | 1.0583101114237 | 2.39052083809218 | 0.0255752123724396 |
| AL021578.1 | 0.781231337600925 | 0.612584411012652 | 0.996307434335159 | 0.0466152226482749 |
| FAM78B-AS1 | 2.02658842864745 | 1.10779741928534 | 3.70741128985234 | 0.0218955463040048 |
| AL606489.1 | 1.41893442748073 | 1.16195811641703 | 1.73274310067081 | 0.000598137842226401 |
| AC011611.2 | 1.65578869427209 | 1.07801952577655 | 2.54321571597168 | 0.0212750548842115 |
| LINC01561 | 1.70104333042569 | 1.09589444864755 | 2.64035319784369 | 0.0178763953190125 |
| AC005785.1 | 0.68664627800085 | 0.485455182576301 | 0.971218617113673 | 0.0335830366540114 |
| AL590226.1 | 0.553291717138954 | 0.402835841631176 | 0.759941625390067 | 0.000256845390144625 |
| AC092168.2 | 1.4050p-value2318 | 1.07975522074038 | 1.82835446314804 | 0.0113713957491088 |
| DEPDC1-AS1 | 3.6482836714252 | 1.48131221778757 | 8.98525887207428 | 0.00488687437314491 |
| LINC00628 | 1.78344182075181 | 1.03703922040385 | 3.06706310178691 | 0.036488582850962 |
| AC090236.2 | 0.574317318348304 | 0.337433638012832 | 0.977497039409698 | 0.0409689028756319 |
| AL078645.1 | 0.472087590764995 | 0.253447498913753 | 0.879340669406794 | 0.0180236337185894 |
| LINC01082 | 0.509645207565587 | 0.315688885211533 | 0.822766494995628 | 0.00581079603729544 |
| AC027117.1 | 0.863612881097122 | 0.756768248192219 | 0.985542416953296 | 0.0295485645600933 |
| AF131215.6 | 0.723823736391051 | 0.556339210069834 | 0.941729060041151 | 0.0160799313995936 |
| HIF1A-AS1 | 1.46786825022085 | 1.03082915832501 | 2.09019815030016 | 0.0333090853720473 |
| LINC01843 | 1.27598226224546 | 1.09349285846641 | 1.48892671859645 | 0.00196833754784615 |
| AP001189.1 | 0.658127510875275 | 0.463088973786613 | 0.9p-value5655421 | 0.0196537948714156 |
| AC087392.2 | 0.545731875211557 | 0.31666935133586 | 0.940486593873274 | 0.0291893885767965 |
| AL135960.1 | 0.133150306521239 | 0.0313665990214717 | 0.565219203859613 | 0.0062676576883574 |
| LINC02323 | 1.469020924129 | 1.17628145589259 | 1.83461404132336 | 0.00069426231852098 |
| MED4-AS1 | 0.590722681702133 | 0.371228692970532 | 0.93999546178684 | 0.026347405534445 |
| LINC02709 | 1.53453667407273 | 1.10046094192867 | 2.13983315023173 | 0.011594454207036 |
| LINC00996 | 0.665230122017328 | 0.492620770728246 | 0.898320049690539 | 0.007823450741278 |
| LINC01215 | 0.752636584835686 | 0.573115721624668 | 0.988389966388149 | 0.0409575375554989 |
| LINC01352 | 0.507602294631889 | 0.290276119483271 | 0.887637915148609 | 0.0174081075470672 |
| SH3BP5-AS1 | 0.731862805972267 | 0.589390492785209 | 0.90877469745817 | 0.00471427788384872 |
| AL512363.1 | 1.53555560541434 | 1.17370894085942 | 2.00895719137392 | 0.00175897059765508 |
| AC119424.1 | 0.493523614938151 | 0.287842713954462 | 0.846175868603552 | 0.0102537869610835 |
| AL157935.2 | 0.73381438704769 | 0.546344410808361 | 0.985611903380592 | 0.0397584295530976 |
| AC004943.2 | 1.41480862044952 | 1.01532284855625 | 1.97147482236275 | 0.0403845440586944 |
| AC011352.1 | 1.6712228681927 | 1.04540266530437 | 2.6716842876587 | 0.0319160031653854 |
| AC018529.1 | 0.475942908595362 | 0.289505883630504 | 0.782442309639973 | 0.00341999321048562 |
| FENDRR | 0.728553060122552 | 0.573375687805045 | 0.925727359396533 | 0.0095562989299475 |
| AL122010.1 | 0.72288848766522 | 0.527731098723325 | 0.990215977157859 | 0.0432584939301937 |
| LINC00592 | 1.36449619624192 | 1.11909713196229 | 1.66370712280709 | 0.00212400372669314 |
| AC092614.1 | 0.642108233614318 | 0.425162113298755 | 0.969754761251693 | 0.0352073267343009 |
| AC131009.1 | 1.44075044331446 | 1.12119395994688 | 1.85138514303909 | 0.00431665494089276 |
| LINC01096 | 1.54923429381301 | 1.07232805401957 | 2.23823939710384 | 0.0197027611665477 |
| NR4A1AS | 0.798712552177233 | 0.665674900358969 | 0.958338282938797 | 0.0156175769943657 |
| AL139099.3 | 1.34441392888347 | 1.05412663096413 | 1.71464106785988 | 0.0170931290643805 |
| CASC15 | 1.22266689254091 | 1.0299524070328 | 1.45144020238989 | 0.021607271773895 |
| AC103681.2 | 0.385872212541705 | 0.162497597959586 | 0.916305017929325 | 0.0309240108540641 |
| AL139123.1 | 0.688757788770027 | 0.4968508441554 | 0.954788136463351 | 0.0252464613720581 |
| KCNQ1-AS1 | 2.05275445874727 | 1.07156949023952 | 3.93236360897593 | 0.0301299286586111 |
| AL590822.2 | 0.253343385558216 | 0.0724152843547727 | 0.886316632986734 | 0.0316473611333471 |
| AC011005.4 | 1.3951520575924 | 1.02657273131754 | 1.89606562148419 | 0.0333771156998353 |
| AC026462.3 | 1.44199648789925 | 1.10528202330413 | 1.88128805795445 | 0.00698134626115307 |
| AL596223.1 | 1.4721193241448 | 1.13469127030676 | 1.90988981869462 | 0.00359976649185412 |
| AL109659.2 | 0.592610234481141 | 0.384856153681561 | 0.912514680231339 | 0.017518199952459 |
| AC010175.1 | 0.71506885413756 | 0.517898077869201 | 0.987305201558871 | 0.0415914346910456 |
| LINC00926 | 0.778046514803529 | 0.618636980526393 | 0.978532480684916 | 0.0319141618968144 |
| LINC01285 | 1.79630104593373 | 1.03798222614542 | 3.10862495170561 | 0.0363327008283341 |
| AP001972.3 | 0.191227624006975 | 0.0444992875406942 | 0.821766059735497 | 0.0261587003556118 |
| AL109811.1 | 0.411819214075691 | 0.193135743269776 | 0.878113301094277 | 0.0216523383563611 |
| AC025741.1 | 2.51392496354158 | 1.1201580587822 | 5.64189908091028 | 0.0254121228405984 |
| AC010976.2 | 0.596731611879937 | 0.414651922133326 | 0.858765141578994 | 0.00544013838651029 |
| AC010999.2 | 0.530157252781644 | 0.309969585207815 | 0.906755778921158 | 0.0204809668015775 |
| LINC02880 | 0.217304674674402 | 0.0517031780708461 | 0.913315649004068 | 0.0371836983238142 |
| AC112722.1 | 0.727904612508924 | 0.535490859820662 | 0.989456897712903 | 0.0425976015838764 |
| MAGEA4-AS1 | 1.33131589997977 | 1.09989797436892 | 1.61142402917496 | 0.00331083681730601 |
| AC021086.1 | 3.37585977335453 | 1.43363726400849 | 7.94931151377062 | 0.00536400128403246 |
| AC084048.1 | 0.456973433015016 | 0.216634528135413 | 0.963949377224845 | 0.0397463157481358 |
| TMPO-AS1 | 1.53253164312843 | 1.20832952326841 | 1.94371915273331 | 0.000430804344454661 |
| LINC02178 | 1.43534151332232 | 1.24210562748632 | 1.65863934135433 | 9.64387752823089e-07 |
| AL591686.1 | 0.377587859920463 | 0.142622211636836 | 0.999652090113096 | 0.0499181746630899 |
| AP000695.2 | 1.46836743899548 | 1.16186528780145 | 1.85572540856442 | 0.00130033028886708 |
| AL391807.1 | 0.38387849614715 | 0.176041942548759 | 0.837088580543136 | 0.0160830193893629 |
| ATP13A4-AS1 | 0.748623201412206 | 0.613004069556357 | 0.914246292195513 | 0.0045231551724713 |
| AL731567.1 | 0.802390641621055 | 0.654329468998991 | 0.983954983329722 | 0.034397487090848 |
| LRP4-AS1 | 1.53926522222334 | 1.02705401184398 | 2.3069258257337 | 0.0366830452546376 |
| AC055855.2 | 2.47828973180903 | 1.23327939073262 | 4.98015294907465 | 0.010808757722504 |
| AC007128.1 | 1.24192886732143 | 1.03346749016694 | 1.49243911991575 | 0.0208268948704935 |
| AL355102.4 | 1.47018314528579 | 1.0011685480188 | 2.15891568403706 | 0.0493076840445843 |
| AF131215.5 | 0.750789734821058 | 0.589868585285936 | 0.955611537846911 | 0.0198659266816302 |
| LINC00543 | 1.28855110031226 | 1.06626147368511 | 1.55718271652218 | 0.00868946752890312 |
| AC068228.2 | 1.38478477956313 | 1.10511959451484 | 1.73522295254532 | 0.00467852814198504 |
| LINC02320 | 1.37027890589388 | 1.08480516417303 | 1.73087697399477 | 0.00822008643476439 |
| AC107021.2 | 1.31325167174825 | 1.01105656617625 | 1.70576999452366 | 0.0411142672691868 |
| AC092071.1 | 0.872567022090753 | 0.769798654944648 | 0.989054999186859 | 0.0329988755683264 |
| LINC01705 | 1.27392221092739 | 1.05291343013222 | 1.54132120747129 | 0.01276206287385 |
| AC024060.2 | 0.774950297259981 | 0.607391647827128 | 0.988732665935928 | 0.0402548503884161 |
| AC017100.1 | 1.27152645512414 | 1.04354184551189 | 1.54931930428481 | 0.0171854537160321 |
| AC026355.2 | 0.719554174544453 | 0.595350436174773 | 0.869669657808655 | 0.00066310970512819 |
| AL161431.1 | 1.20837689889694 | 1.08442009682364 | 1.34650283046649 | 0.000608945582181544 |
| AC018647.1 | 0.375405975710417 | 0.16099237164053 | 0.875380896392801 | 0.0233245497349285 |
| AC024361.1 | 0.763221752743526 | 0.601944696510121 | 0.96770923847006 | 0.0256828761862788 |
| AC105020.6 | 0.451216153315395 | 0.240200410328281 | 0.847608947605409 | 0.0133629490302452 |
| AC010275.1 | 1.26966099422369 | 1.06587120052151 | 1.51241448259823 | 0.0074818327208163 |
| AC106038.1 | 4.58337503713214 | 1.21998667012323 | 17.2193084116928 | 0.0241707844181759 |
| AC104072.1 | 1.4130094967666 | 1.04764382878414 | 1.9057964005475 | 0.0235201046604501 |
| AC026310.2 | 0.205976293594797 | 0.0461858491552352 | 0.918598105243259 | 0.0383334729625392 |
| AL117379.1 | 0.790476073321683 | 0.626498342339263 | 0.997372826496157 | 0.0474634506511748 |
| LINC02544 | 1.19337605492358 | 1.01363517013753 | 1.40498914246607 | 0.0337901328162736 |
| ARRDC1-AS1 | 0.671758869179143 | 0.471402580683936 | 0.957270911979583 | 0.027692572392635 |
| AC090948.1 | 0.587985398275087 | 0.428449745931909 | 0.806925040491581 | 0.00100789988396103 |
| LINC00621 | 1.89591122342543 | 1.17846099249288 | 3.0501470901526 | 0.00836841268664479 |
| LINC01781 | 0.779215440350319 | 0.623364044667513 | 0.974032281255802 | 0.0284482226794988 |
| KTN1-AS1 | 1.43649460679113 | 1.06236544928902 | 1.94237939187592 | 0.0186239953327427 |
| LINC01031 | 0.379257570235503 | 0.159671686254366 | 0.900825362060734 | 0.0280495726822727 |
| SLC2A1-AS1 | 1.46800991772468 | 1.06129731084678 | 2.03058379260243 | 0.0203740950885903 |
| AC039056.2 | 1.41754485870238 | 1.04157166139268 | 1.92923204510647 | 0.0264873630225787 |
| AP005137.2 | 1.44827398403185 | 1.16686235516444 | 1.79755351909344 | 0.000779751332910193 |
| AC105036.3 | 2.02328829255339 | 1.27822020185437 | 3.20265280492727 | 0.00263362509748013 |
| AC079949.1 | 1.31226608883776 | 1.11467150530373 | 1.54488769087564 | 0.00109946044845608 |
| AL606834.1 | 1.62334734176776 | 1.21706170047658 | 2.1652612936489 | 0.000978684062612339 |
| LINC01585 | 2.30299168154443 | 1.09968797912056 | 4.82297777730036 | 0.0269715383510012 |
| AL445231.1 | 1.610696182442 | 1.03072334021154 | 2.51701120069793 | 0.036364734821309 |
| AC123595.1 | 0.629052401238876 | 0.459254133650023 | 0.861629530385342 | 0.00387985915407917 |
| AL024497.1 | 1.61343117989517 | 1.28626911270631 | 2.02380679637162 | 3.51477219263645e-05 |
| LINC02313 | 1.1958024336413 | 1.01115515591205 | 1.41416819361681 | 0.0366539725915519 |
| AP000438.1 | 0.384935272253049 | 0.161964607691554 | 0.914861375805723 | 0.0306626430676977 |
| AC012178.1 | 0.283290804661673 | 0.0888594387309275 | 0.903153127591454 | 0.0329936286882876 |
| AC087521.1 | 0.541317542224595 | 0.336590597496712 | 0.870567044056954 | 0.0113499016412035 |
| FAM66C | 2.1876657543003 | 1.30770812910871 | 3.65974742070329 | 0.00286529083670204 |
| AC009065.4 | 0.671259675291714 | 0.495448620879196 | 0.909457676707516 | 0.010097520796214 |
| LINC01800 | 0.151816123054503 | 0.0395737284768521 | 0.582410000432015 | 0.00599599432486274 |
| AC138649.1 | 0.793077083270454 | 0.643494755546457 | 0.977430281424201 | 0.0297023529594622 |
| GRPEL2-AS1 | 0.558750463406025 | 0.334234761297741 | 0.934080222967393 | 0.0264143038437858 |
| C8orf34-AS1 | 0.897152685327899 | 0.805405574500271 | 0.999351092510707 | 0.0486373357671817 |
| FOCAD-AS1 | 0.451121210317793 | 0.243568081171359 | 0.835537831639826 | 0.011362554170775 |
| GSEC | 1.32100637274663 | 1.0720660765955 | 1.62775212734918 | 0.00897117587914961 |
| AC084819.1 | 1.80908576702104 | 1.25186087725214 | 2.61434107568081 | 0.00160098022847125 |
| LINC00941 | 1.3237022600398 | 1.12627211389515 | 1.55574097202374 | 0.000666695763390417 |
| LINC02198 | 0.772966078842904 | 0.602604247954053 | 0.991490785321066 | 0.0426382416544536 |
| AC046134.2 | 1.44753413581352 | 1.06708899067113 | 1.96361792939834 | 0.0174380444582234 |
| AC005562.1 | 1.82040905985848 | 1.0845916238657 | 3.05542572180622 | 0.0233719435747503 |
| AC027601.2 | 0.540520163882373 | 0.336213745202864 | 0.868977106772189 | 0.01109445651535 |
| MIR600HG | 0.743645785823967 | 0.580653006950372 | 0.952391614534445 | 0.0189565097246458 |
| AC024075.2 | 0.694020068639371 | 0.52235887782514 | 0.922093748419903 | 0.0117545639658157 |
| MIR22HG | 0.709035164429561 | 0.536078097783968 | 0.937794076041973 | 0.0159467313067741 |
| AL049871.1 | 0.423268017342976 | 0.180720835601823 | 0.991340118082359 | 0.0477041514599645 |
| NPAS2-AS1 | 2.16700620061353 | 1.11005237488235 | 4.23035523345934 | 0.0234591820908147 |
| AL360270.1 | 1.55473305179405 | 1.14772628870164 | 2.10607257682953 | 0.00437623710208483 |
| DLEU1 | 1.4458502197655 | 1.03372822932483 | 2.02227509967616 | 0.0312608189972166 |
| LINC00623 | 1.34739158310066 | 1.03672572376582 | 1.75115176231567 | 0.0257693552183677 |
| LINC01150 | 0.693665093871957 | 0.535771683823984 | 0.898090132390179 | 0.00550979786357547 |
| AC060234.2 | 0.420971944396758 | 0.197804897686028 | 0.895920070950321 | 0.0247575438150434 |
| AC034102.8 | 0.674528308055787 | 0.471067157829174 | 0.965867458188621 | 0.0315901465604933 |
| AC023509.2 | 0.499154167720409 | 0.26420668899875 | 0.943030186316868 | 0.0323002240679639 |
| EMX2OS | 1.30361782171785 | 1.01019606578396 | 1.68226692090863 | 0.0415563526079374 |
| LINC01638 | 1.4809555768006 | 1.05334884008249 | 2.08214917698589 | 0.0238865638039529 |
| AC005034.3 | 1.72665623543561 | 1.25001364533673 | 2.38504736847537 | 0.000919945154348912 |
| ZRANB2-AS1 | 0.492058249857329 | 0.24250971005006 | 0.998398460839684 | 0.0494832726683024 |
| FGF12-AS2 | 1.33532378480569 | 1.0225254376256 | 1.74380953730432 | 0.0337083171618166 |
| AC137932.3 | 0.728955441073606 | 0.537666788072506 | 0.988299904064668 | 0.0417755604047277 |
| LINC02518 | 1.51109329582529 | 1.14821390787927 | 1.98865641063828 | 0.00321567723758488 |
| GCC2-AS1 | 1.5552136025112 | 1.03043580660073 | 2.34724893481216 | 0.0354901492484392 |
| AC108451.2 | 1.2041350997626 | 1.02545620789063 | 1.41394759456654 | 0.0234087000989377 |
| AC093159.1 | 0.65593272069263 | 0.434824827707108 | 0.98947370678899 | 0.0443874537793642 |
| AL357518.1 | 0.196447552791344 | 0.0606658888582689 | 0.636134106398209 | 0.00663773813792759 |
| AC005277.2 | 0.600293827497012 | 0.376061611688055 | 0.958227769416484 | 0.032452623019735 |
| AC091891.1 | 0.606483593449156 | 0.403829218364965 | 0.910836394187253 | 0.0159501814259097 |
| LINC00892 | 0.680201582032888 | 0.46983976485547 | 0.984748901239487 | 0.0412135627553918 |
| ABCA9-AS1 | 1.75691266016547 | 1.25374818804722 | 2.46201121156354 | 0.00106215898326819 |
| AC107959.1 | 0.509169330608159 | 0.310330492909018 | 0.835410677183981 | 0.00754440268924263 |
| AC005264.1 | 0.346625426252458 | 0.126104951715258 | 0.952771358225422 | 0.0400004869489068 |
| AC004704.1 | 1.27970893080912 | 1.08279044234317 | 1.51243941907053 | 0.00381608408090624 |
| AL359962.1 | 1.33815769108666 | 1.05754547970847 | 1.69322836754784 | 0.0152693376410329 |
| AC138625.1 | 1.49652292877169 | 1.02198036016828 | 2.1914128329928 | 0.038293984059839 |
| AC010998.1 | 0.61474189648921 | 0.398857270860167 | 0.947475768673248 | 0.0274954151359823 |
| LINC01559 | 1.15989192557482 | 1.0094174499902 | 1.33279772310924 | 0.0364226614112661 |
| AL031600.2 | 0.488073209860818 | 0.263961880629104 | 0.902461588832827 | 0.022183172878104 |
| AL365181.2 | 1.15760635334714 | 1.02487726187778 | 1.30752483166073 | 0.0185006820613167 |
| AC009226.1 | 1.67358570030072 | 1.1398299054889 | 2.45728690110974 | 0.00859327928117393 |
| AL162632.3 | 2.80333032724764 | 1.18500424165644 | 6.63175763209191 | 0.018958619309176 |
| AC105020.5 | 0.588113790757107 | 0.388847303156387 | 0.889495254489625 | 0.0119132928722638 |
| AC012213.1 | 1.26953811455513 | 1.0615533931467 | 1.51827221759485 | 0.00894084135587947 |
| AC022167.3 | 0.564118540798543 | 0.319782177832608 | 0.995145289926871 | 0.0480678882354061 |
| AL590428.1 | 1.93021228417084 | 1.13915719628362 | 3.27059292090573 | 0.0145171102949913 |
| AC096920.1 | 0.512078290353947 | 0.279510514060001 | 0.938154961124402 | 0.0302629525256453 |
| AC087752.3 | 0.633976643399663 | 0.469142802985739 | 0.856725035145688 | 0.00301169759059547 |
| PLUT | 1.60236839330936 | 1.24553428992345 | 2.06143218107198 | 0.000244252900759884 |
| AC135012.3 | 0.114622450656825 | 0.0187580256682697 | 0.700409863325894 | 0.0189989283241535 |
| LINC02448 | 1.53422447731551 | 1.07135135636052 | 2.19708010151804 | 0.0194845421807119 |
| AP000346.1 | 0.476973697184059 | 0.255233836673593 | 0.891354809262125 | 0.0203151944707534 |
| C20orf197 | 0.717053210273974 | 0.585162017168309 | 0.878671703355489 | 0.00134046010182534 |
| CTBP1-DT | 1.62214203489653 | 1.13486179447398 | 2.31864778089382 | 0.00795295316605087 |
| LINC00707 | 1.43729175269463 | 1.20654703743029 | 1.71216497846929 | 4.84688477064113e-05 |
| AC018645.3 | 1.29543472679029 | 1.02095074077271 | 1.64371410329173 | 0.0331193480157266 |
| AL049555.1 | 1.20322187073398 | 1.06181975846096 | 1.36345444570648 | 0.00372736207598432 |
| AC010536.1 | 0.597325968610821 | 0.374926824006559 | 0.951647868146704 | 0.0301183330801793 |
| AC026356.1 | 1.33664092307147 | 1.02929628756705 | 1.73575770048909 | 0.0295129823437642 |
| AC084880.3 | 0.274690671688572 | 0.0972540475848858 | 0.775854239350396 | 0.0147268595144745 |
| AC021739.2 | 0.676817001063352 | 0.459788960738787 | 0.996285887752387 | 0.0478354851779791 |
| FLG-AS1 | 1.74874084356609 | 1.0184700346928 | 3.00263575145699 | 0.0427322579250973 |
| LINC02728 | 0.506964974830986 | 0.279471746246491 | 0.919640318412364 | 0.0253737030802873 |
| AL024497.2 | 1.42229806458813 | 1.16235140354164 | 1.74037883755923 | 0.000624054860968729 |
| MIR223HG | 0.686576347137185 | 0.54175644971888 | 0.870108848160174 | 0.00186396353457151 |
| FOXO6-AS1 | 0.761037412872447 | 0.592426918903758 | 0.977636102126003 | 0.0326010950876035 |
| MAP3K20-AS1 | 1.60727979161612 | 1.12067943419336 | 2.30516260914255 | 0.00990236432795524 |
| CRIM1-DT | 1.23212057001043 | 1.01727099269257 | 1.49234678856278 | 0.0327514953438433 |
| LINC01711 | 1.37083046532101 | 1.07758010728594 | 1.7438853519533 | 0.0102175901858406 |
| LINC01338 | 1.71908769644688 | 1.23034132869427 | 2.40198588729144 | 0.00150065083331942 |
| LINC02718 | 0.501143615765944 | 0.252854014353594 | 0.993240800487192 | 0.047771487616917 |
| LINC00862 | 1.34003318669221 | 1.10661035470303 | 1.62269305885755 | 0.00272341017891716 |
| C2-AS1 | 0.671125361308153 | 0.478164730710514 | 0.941954146057003 | 0.0211276434211226 |
| ZEB2-AS1 | 0.401110934362071 | 0.165571331477604 | 0.971726084636679 | 0.0430221583863678 |
| COL4A2-AS1 | 0.578919216423684 | 0.345062342781576 | 0.971266399117797 | 0.0384152666443087 |
| LANCL1-AS1 | 0.744629345291653 | 0.555445925324144 | 0.998248140079342 | 0.0486451934424154 |
| TM4SF19-AS1 | 1.49570944391383 | 1.12947450553528 | 1.98069697868282 | 0.0049596643296152 |
| AC127537.1 | 0.588403274213225 | 0.347375330829542 | 0.996669545526058 | 0.0485672948855419 |
| UCKL1-AS1 | 0.727392738951484 | 0.541108063915022 | 0.97780874461785 | 0.0349761107104989 |
| FRMD6-AS1 | 2.21770057860774 | 1.4440215720237 | 3.40590192808863 | 0.000274240073526633 |
| CASC19 | 1.14961637387624 | 1.00332853829986 | 1.31723334544419 | 0.0446627936797417 |
| AC133785.1 | 1.38442800681299 | 1.07140102016219 | 1.78891084662031 | 0.0128707734743972 |
| LINC00857 | 1.47471111228152 | 1.16779387168508 | 1.86229172580645 | 0.00110298460064798 |
| AP000695.1 | 1.36169425870132 | 1.08976411215545 | 1.70147946101169 | 0.00660221712260455 |
| SEMA6A-AS2 | 2.64570708532339 | 1.04581760117509 | 6.69310401112524 | 0.0399208886153941 |
| LINC02147 | 0.109326570489482 | 0.0231616766141537 | 0.516037729655885 | 0.00518148129598915 |
| AL353152.1 | 0.535467072857953 | 0.315285457648747 | 0.909413926837368 | 0.0208142829745964 |
| AC007686.2 | 0.0854688134725254 | 0.0163329595952061 | 0.447250116172785 | 0.00358101027045089 |
| CADM3-AS1 | 0.382084793483402 | 0.184989704435459 | 0.789172510204148 | 0.00932926639908327 |
| TARID | 1.59119614386137 | 1.23051349812938 | 2.05760048312211 | 0.000397756237337912 |
| AC114763.1 | 1.6354540153737 | 1.06934545297605 | 2.50125890464872 | 0.0232528858583993 |
| AL049836.1 | 1.36326561722463 | 1.1316344783982 | 1.64230869470988 | 0.00110817990200653 |
| LINC01312 | 2.94454935574121 | 1.98526164367134 | 4.36736937724837 | 7.89659833681886e-08 |
| LINC01614 | 1.14504850261398 | 1.00490119784429 | 1.30474127819845 | 0.0420159435998734 |
| MHENCR | 0.769070929893547 | 0.635648418245501 | 0.93049880756391 | 0.00691424920800982 |
| AL157931.1 | 1.38099870406875 | 1.13957635529321 | 1.67356703373234 | 0.000992367671864042 |

**Table S4.** **Results of multivariate Cox regression.**

| **id** | **coef** | **HR** | **HR.95L** | **HR.95H** | **pvalue** |
| --- | --- | --- | --- | --- | --- |
| **AC018529.1** | -1.23255025100815 | 0.291548107935803 | 0.148436893663307 | 0.572635933986524 | 0.00034536601511875 |
| **AC025741.1** | 2.77627384340203 | 16.0590707131145 | 4.51289733705642 | 57.1459381650871 | 1.81256527346068e-05 |
| **AC010999.2** | -0.755763688557765 | 0.469651810027002 | 0.207406221801081 | 1.06348218846196 | 0.0699295505599207 |
| **LINC02880** | -0.963608325909229 | 0.381513773294536 | 0.0849146290263917 | 1.71410699054219 | 0.208754984499642 |
| **AC068228.2** | 0.450520603660357 | 1.5691288671198 | 1.16641189013302 | 2.11088846269208 | 0.00290889736530107 |
| **AC026355.2** | -0.293174537349467 | 0.745891943332623 | 0.591509735225787 | 0.94056742940359 | 0.013218999504823 |
| **AC024361.1** | -0.365617232310227 | 0.693768302484239 | 0.486434060817067 | 0.989475236835585 | 0.0435523898697605 |
| **AC026310.2** | -1.31320570094112 | 0.268956478901124 | 0.0444962012829454 | 1.6257025421767 | 0.152548400706217 |
| **AC105036.3** | 0.92627097436946 | 2.52507552802597 | 1.26695530347312 | 5.03254250939793 | 0.00847797669992962 |
| **AL606834.1** | 0.434614815485002 | 1.54436807770092 | 1.09362044102724 | 2.18089628718109 | 0.0135792838589517 |
| **LINC01585** | 1.64257045773309 | 5.16843770292896 | 2.02355838350777 | 13.2008784657609 | 0.000596448086861196 |
| **FAM66C** | 0.634082385554587 | 1.88529137689165 | 0.979100612870954 | 3.63019237150704 | 0.0578565353472798 |
| **LINC01800** | -1.08941744801224 | 0.336412414354901 | 0.0873833797860889 | 1.2951354457694 | 0.113204672171739 |
| **AC084819.1** | 0.59383588835849 | 1.81092160252396 | 1.18315647516477 | 2.77176951597314 | 0.00624943074994754 |
| **LINC00623** | 0.335311057635344 | 1.39837529289021 | 1.00688761412134 | 1.94207718154545 | 0.0454005800974854 |
| **LINC01150** | -0.348597163666179 | 0.705677345494721 | 0.48163328345518 | 1.03394124337094 | 0.0736635646885793 |
| **LINC01638** | 0.613719403998481 | 1.84728945271563 | 1.1888678692926 | 2.87035961712459 | 0.00634617252351765 |
| **AL031600.2** | -0.725701222728507 | 0.483985068606229 | 0.203850063901779 | 1.1490874329411 | 0.0999776246596399 |
| **AL365181.2** | 0.161352678439542 | 1.1750993279391 | 1.00901776752972 | 1.36851745822429 | 0.0379460249374305 |
| **AC009226.1** | 0.871232108022066 | 2.38985359805215 | 1.28822278471362 | 4.43355007215813 | 0.00572340148682478 |
| **LINC02448** | 0.587916309149962 | 1.80023337477364 | 1.1080430078412 | 2.92483250263274 | 0.0175825170824611 |
| **AP000346.1** | -0.671402962293118 | 0.51099117331023 | 0.235181753204659 | 1.11025611316768 | 0.0899248521651075 |
| **C20orf197** | -0.306030396231082 | 0.736364236418431 | 0.573692978892258 | 0.945161102935397 | 0.0162707306392266 |
| **AC084880.3** | -1.05826939029022 | 0.347055909238149 | 0.0998997462255219 | 1.20568678788442 | 0.095798394059228 |
| **LINC01711** | 0.445456271965488 | 1.56120236622078 | 1.07532421130335 | 2.26662136188598 | 0.0191943831241195 |
| **LINC00862** | 0.215303454700717 | 1.2402381959789 | 0.953156454145175 | 1.61378625311256 | 0.108976840142592 |
| **`FRMD6-AS1`** | 0.494099598336426 | 1.63902179650892 | 0.892123520405103 | 3.01123374508887 | 0.111354056617852 |
| **`SEMA6A-AS2`** | 1.12399514646074 | 3.07712323668961 | 0.980240933130638 | 9.65955113049053 | 0.054132935410412 |
| **AC007686.2** | -3.39908919261594 | 0.0334036804279731 | 0.00395885798721421 | 0.281850440136482 | 0.00178551288455134 |
| **LINC01312** | -0.626033108389434 | 0.53470873099403 | 0.265653892506305 | 1.07626289343553 | 0.0794230437222385 |
